# Artificial intelligence-powered discovery of small molecules inhibiting CTLA-4 in cancer

**DOI:** 10.1038/s44276-023-00035-5

**Published:** 2024-01-23

**Authors:** Navid Sobhani, Dana Rae Tardiel-Cyril, Dafei Chai, Daniele Generali, Jian-Rong Li, Jonathan Vazquez-Perez, Jing Ming Lim, Rachel Morris, Zaniqua N. Bullock, Aram Davtyan, Chao Cheng, William K. Decker, Yong Li

**Affiliations:** 1 Department of Medicine, Baylor College of Medicine, Houston, TX 77030, USA.; 2 Department of Cancer Biology, The University of Texas MD Anderson Cancer Center, Houston, TX 77054, USA.; 3 Department of Medical, Surgery and Health Sciences, University of Trieste, 34147 Trieste, Italy.; 4 Department of Pathology and Immunology, Baylor College of Medicine, Houston, TX 77030, USA.; 5 Atomwise Inc., 717 Market St, Suite 800, San Francisco, CA 94103, USA.; 6 Dan L. Duncan Comprehensive Cancer Center, Baylor College of Medicine, Houston, TX 77030, USA.; 7 Center for Cell and Gene Therapy, Baylor College of Medicine, Houston, TX 77030, USA.

## Abstract

**BACKGROUND/OBJECTIVES:**

Checkpoint inhibitors, which generate durable responses in many cancer patients, have revolutionized cancer immunotherapy. However, their therapeutic efficacy is limited, and immune-related adverse events are severe, especially for monoclonal antibody treatment directed against cytotoxic T-lymphocyte–associated protein 4 (CTLA-4), which plays a pivotal role in preventing autoimmunity and fostering anticancer immunity by interacting with the B7 proteins CD80 and CD86. Small molecules impairing the CTLA-4/CD80 interaction have been developed; however, they directly target CD80, not CTLA-4.

**SUBJECTS/METHODS:**

In this study, we performed artificial intelligence (AI)-powered virtual screening of approximately ten million compounds to identify those targeting CTLA-4. We validated the hits molecules with biochemical, biophysical, immunological, and experimental animal assays.

**RESULTS:**

The primary hits obtained from the virtual screening were successfully validated in vitro and in vivo. We then optimized lead compounds and obtained inhibitors (inhibitory concentration, 1 micromole) that disrupted the CTLA-4/CD80 interaction without degrading CTLA-4.

**CONCLUSIONS:**

Several compounds inhibited tumor development prophylactically and therapeutically in syngeneic and CTLA–4–humanized mice. Our findings support using AI-based frameworks to design small molecules targeting immune checkpoints for cancer therapy.

## INTRODUCTION

The immune system plays a pivotal role in the eradication of cancer. Unfortunately, however, cancer cells evolve mechanisms to suppress or evade the antitumor immune response. One method of immune suppression is the upregulation of immune checkpoint proteins [1], such as cytotoxic T-lymphocyte–associated protein 4 (CTLA-4), programmed cell death protein 1 (PD-1), and PD-1’s ligand, programmed death-ligand 1 (PD-L1). The upregulation of CTLA-4 in activated T cells is a phenomenon observed in tumor tissues (in which activated effector T cells are suppressed) and autoimmune diseases (in which T cells are hyperactivated). CTLA-4 and CD28 are homologous glycoproteins of the immunoglobulin superfamily, yet they have opposing effects on T-cell function. When CTLA-4 is expressed at high levels on the surface of T cells, it binds to B7 peripheral membrane proteins (CD80 and/or CD86) on activated, antigen-presenting cells [2–4]. CTLA-4 binds to B7 proteins with an affinity approximately 10 to 100 times higher than that of the prototypic costimulatory receptor CD28 [3, 5, 6], and subsequently, it inhibits CD28-mediated T-cell activation, cell proliferation, and cytokine production. Although the mechanisms by which CTLA-4 achieves its critical functions in immune homeostasis are highly debated, CTLA-4 indisputably safeguards against autoimmunity during the immune system’s beneficial antitumor and antipathogenic responses.

In the past decade, the inhibition of immune checkpoints has revolutionized cancer immunotherapy. The U.S. Food and Drug Administration (FDA) has approved several monoclonal antibodies for use against immune checkpoints, including CTLA-4, PD-1, PD-L1, and lymphocyte activation gene 3. After its encouraging results in late-stage melanoma patients, ipilimumab, an anti-CTLA-4 antibody, became the first immune checkpoint blockade therapy to receive FDA approval [7, 8]. This anticancer drug was also approved for the treatment of renal cell carcinoma [9], colorectal cancer [10], hepatocellular carcinoma [11], non-small cell lung cancer [12], and malignant pleural mesothelioma [13]. However, although anti-CTLA-4 therapy with ipilimumab improves patients’ overall survival, it has limitations. First, patients treated with this therapy have lower response rates than patients treated with anti-PD-1 antibodies [14]. Second, anti-CTLA-4 treatments cause more immune-related adverse events (irAEs) associated with significant morbidity and mortality (grade 3 and 4 irAEs) than do anti-PD-1 and anti-PD-L1 antibody treatments alone. Statistically, about 90% of melanoma patients receiving ipilimumab with an anti-PD-1 antibody experience such toxicities [15]. Hence, improving the safety and efficacy of anti-CTLA-4 agents is critical.

A recent report demonstrated that, in mice, the irAEs associated with anti-CTLA-4 antibody treatment were mediated by the lysosomal degradation of CTLA-4 and that the avoidance of degradation led to more effective tumor rejection with fewer irAEs [16]. Another study in mice showed that modifying tremelimumab into a new pH-sensitive antibody or generating a completely new antibody resistant to lysosomal degradation inhibited but did not degrade CTLA-4, resulting in dramatically attenuated irAEs. Thus, more efficient approaches to tempering CTLA-4 without degradation could substantially improve patient outcomes. Small chemical inhibitors generally do not degrade their targets and have favorable biosafety profiles [17, 18]. Another key aspect of CTLA-4 is its rapid and constitutive endocytosis from the plasma membrane; because of this endocytosis, approximately 90% of CTLA-4 is located inside nonactivated effector T cells [19] and, consequently, inaccessible to antibodies. If intracellular CTLA-4 suppresses antitumor immunity, anti-CTLA-4 compounds may elicit different immune responses than anti-CTLA-4 blocking antibodies.

Since the discovery that CTLA-4/CD80 interactions regulate the immune response, several teams have developed small compounds that disrupt such interactions [20–23]. These compounds block CD80’s interactions with both CD28 and CTLA-4, yet they do not bind CTLA-4 directly. RhuDex (AV1142742), for example, is a novel, orally bioavailable, small-molecule modulator of T cells that binds to CD80 on the surface of antigen-presenting cells and inhibits its interaction with CD28 (but not with CTLA-4) [24–26]. The development of RhuDex was discontinued after a failed phase II clinical trial for rheumatoid arthritis. EL 26, a small peptide derived from the CD80-binding domain (MYPPPY motifs), also targets CD80 but not CTLA-4 [27]. Currently, there are no reported efforts to develop small-molecule CTLA-4 inhibitors. Therefore, in this study, we used artificial intelligence (AI) to drive the discovery of anti-CTLA-4 small molecules.

AI is a problem-solving system with vast applications across various fields. It has evolved significantly since its development during Alan Turing’s investigations into whether machines could think [28]. Today, AI surpasses humans in specific tasks. Machine learning is a broad field encompassing different techniques and algorithms, including deep learning. Deep learning utilizes neural networks and differs from traditional AI and machine learning because it involves learning directly from data without explicit programming. It can also leverage datasets to inform its own algorithms. Deep learning allows computing devices to process undistributed data like facial recognition data, text, and images to determine the distinguishing features that separate different categories of data. A generative AI model, which is a type of deep-learning model, can learn from raw data and generate statistically predictable outputs [29].

There are several approaches to traditional drug development, including high-throughput screening (HTS), fragment screening, the tissue-based physiological approach, virtual screening, the hit-to-lead method, focused screening of similar compounds, and structural-aided drug design. HTS involves screening compound libraries against drug targets and is commonly used by major, multinational pharmaceutical companies for large-scale screenings against a drug target [30, 31]. Fragment screening is a method that can help find minuscule molecular-weight compound libraries and that uses protein-structure generation to aid compound development [32]. Advantageously, because this method enhances fragment-fitting in the chemical space of interaction with the targeted molecule or protein of interest, it yields compounds with enhanced potency. The physiological- or tissue-based physiological approach looks for compounds that single out one specific molecular component of the target that will produce the final desired *in vivo* effect. Disadvantages to this approach are the low throughput as the primary focus is on mimicking the tissue’s complexity [33]. Current virtual screens use docking models of known protein crystal structures and AI technology to explore virtual compound libraries, optimizing resource usage. This method enables the exploration of a broader chemical space, yielding more potential hit compounds. The top leads can then undergo efficient, focused screening approaches that are affordable, fast, require little storage space, and can target previously unexplored regions of the targeted proteins [34]. The hit-to-lead method involves soaking small compounds into protein targets with known crystal structures to look for compounds that can be used as building blocks of larger molecules. The focused screening of similar compounds is a convenient approach, but it may lack the novelty needed for patents. Finally, structural-aided drug design uses crystal structures to design molecules. This approach usually exploits the specific knowledge of an already known compound-to-protein of interest docking region. Novel modifications are made to the compound to increase its potency or selectivity [33].

Historically, traditional HTS has been the most common method of drug development, but it takes around 6 months for the initial step and an additional 18 months for subsequent cycles of engineering. HTS produces good candidates in 50% of cases. AI screening, on the other hand, can complete the first screening in less than 3 months and predict optimal chemical modifications in 6 months using machine-learning models. Thus, AI enables faster drug discovery, and it also reduces the number of candidates for rigorous assessment. Ultimately, it produces a suitable candidate with 90% confidence. Biopharmaceutical companies are increasingly using AI to accelerate their drug development process [33].

In this study, we screened a library of approximately 10 million compounds using an AI algorithm based on deep convolutional neural networks trained to recognize a putative binding pocket on CTLA-4. Using molecular and cellular assays to validate the hits from the AI screening, we identified several lead compounds that successfully bind to CTLA-4 and inhibit its interaction with CD80. We then obtained optimized compounds to reduce tumorigenesis in CTLA-4–humanized mice. Our findings support the use of small molecules as alternatives to antibodies targeting CTLA-4 in cancer therapy.

## RESULTS

### AI screening

There were 20 structures of human CTLA-4, its extracellular domain (ECD), or CTLA-4 complexed with CD80/CD86 or anti-CTLA-4 antibodies in the RCSB protein databank. Using the ICM Pocket Finder method [35], we identified a binding pocket near CTLA-4’s interface with CD80 and CD86 using 1AH1, the nuclear magnetic resonance spectroscopy-determined structure of the ECD of human CTLA-4 [36]. This putative binding site, which consists of residues from the peptide sequences of ^60^YASPGKATEV and ^134^LMYPPPYY (positions determined using the full length of CTLA-4), is at the edge of the CTLA-4 binding interface with CD80/CD86. Thus, a small molecule bound to this pocket can weaken CTLA-4’s binding to CD80/CD86 through steric or allosteric effects. Using the AtomNet algorithm [37], we screened this pocket against approximately 10 million commercially available compounds from the online drug discovery platform MCule.com (https://mcule.com/). We then selected 30,000 top-ranking compounds and clustered them using a Tanimoto similarity cutoff of 0.35, the Butina clustering algorithm [38], and extended connectivity fingerprints with up to four bonds. Drug-like properties were also used to filter the results and exclude likely false positives, such as aggregators, autofluorescent molecules, and pan-assay interference compounds [39]. Finally, 72 compounds were successfully synthesized for testing (purity, >95%) (Supplementary Table S1).

### Amplified luminescent proximity homogeneous (Alpha) LISA assay

**Fig. 1:**
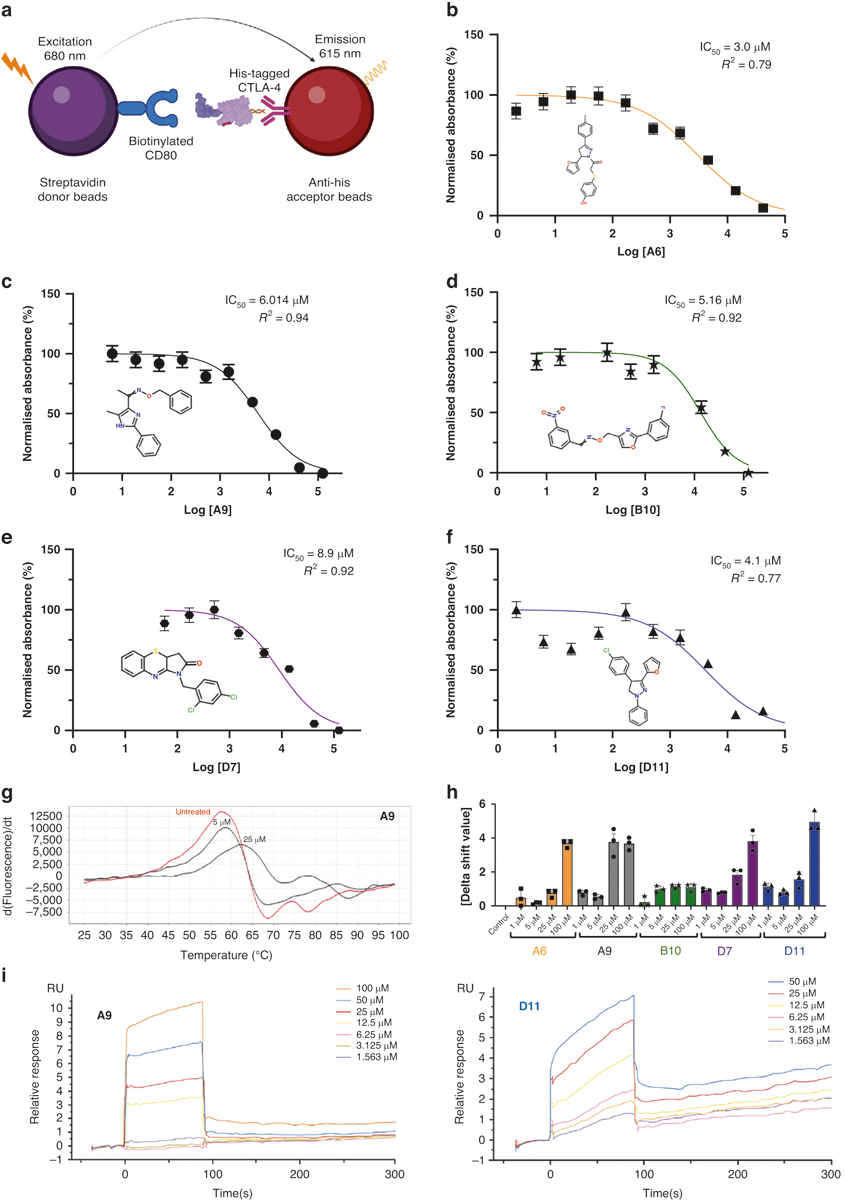
AlphaLISA, protein thermal shift, and surface plasmon resonance of anti-CTLA-4 lead compounds. **a** The AlphaLISA method. The method consisted of 2 components: (1) streptavidin donor beads containing biotinylated CD80, and (2) anti-His acceptor beads containing His-tagged CTLA-4 antibodies. CD80 and CTLA-4 disrupt the 2-component system, reducing luminescence emission at 615 nm. **b**–**f** IC_50_ of the top 5 leading compounds. The values were measured as a reduction in 615-nm luminescence (color-coded panels). The lead compounds were (**b**) A6, (**c**) A9, (**d**) B10, (**e**) D7, and (**f**) D11 (*n* = 3). **g** PTS dose-response curves. The absolute value of the delta thermal shift indicates binding between CTLA-4 purified protein and lead compounds. The dose-dependent (5-μM and 25-μM) PTS changes in melting temperatures for the representative A9 lead compound is represented. **h** Summarized PTS data for the top 5 lead compounds’ dose-response results (5 μM, 25 μM, 50 μM, and 100 μM). **i** SPR dose-response curves for D11 and A9 are illustrated. The values are the means of 3 experiments done in triplicate. AlphaLISA amplified luminescent proximity homogeneous LISA, CTLA-4 cytotoxic T-lymphocyte–associated protein 4, d delta, dT delta temperature, IC_50_ half-maximal inhibitory concentration, PTS protein thermal shift, RU relative unit, SPR surface plasmon resonance.

We first evaluated the inhibitory impacts of the compounds against the interaction between CTLA-4 and CD80 using an amplified luminescent proximity homogeneous (Alpha)LISA assay. In this assay, a biotinylated CD80 binds to streptavidin-coated alpha donor beads, while anti-His AlphaLISA acceptor beads capture His-tagged CTLA-4. Upon binding of CD80 to CTLA-4, the donor and acceptor beads draw near, thus excitation of the donor bead leads to the release of singlet oxygen molecules. Consequently, an energy transfer cascade occurs in the acceptor beads, culminating in an intense peak of light emission at 615 nm (Fig. 1a). The 72 compounds were screened, and the 5 that inhibited CTLA-4 most substantially (half-maximal inhibitory concentration [IC_50_] values, 3.0 to 8.9 μM) were selected for a dose-response assay (Fig. 1b–f). Unlike ipilimumab, these 5 compounds did not reduce membrane expression levels of CTLA-4 in Jurkat cells overexpressing exogenous CTLA-4 after 45 minutes of coincubation (Supplementary Fig. S1a). After 24 h of coincubation, only 1 compound (B10) moderately reduced the membrane expression levels of CTLA-4 (Supplementary Fig. S1b). The compounds’ inhibitory activity against CTLA-4 was confirmed through a luciferase reporter assay involving 2 genetically engineered cell lines. First, we developed a CTLA-4 effector cell line using Jurkat T cells expressing human CTLA-4 and a luciferase reporter driven by a native promoter responsive to T-cell receptor (TCR)/CD28 activation. We also managed to establish an adjacent antigen-presenting cell line utilizing Raji cells, which are capable of expressing constitutively active engineered proteins on their surfaces without requiring antigens for effectivity. The Raji cells will also be able to naturally produce endogenous levels of both CD80 as well as CD86 molecules within the target APCs themselves as they transclude from one host to another. In vitro co-culturing experiments successfully demonstrated that such interactions can lead to competitive mechanisms employed by antibodies targeting CTLA-4 or even other immunomodulators toward other similarly accessed sites, thus, hindering a potential pathway’s induction exclusively based upon alternative ligands despite upstream effects. Some levels of CTLA-4 on the membrane of the Jurkat cells may be pronounced due to selective localization placed upon them along a relative time scale over prolonged periods of observation, which can then consequently lead to variations and subtypes. The addition of ipilimumab or 1 of the 4 compounds (A9, B10, D7, or D11) blocked the interaction of CTLA-4 with CD80 and CD86 and resulted in promoter-mediated luminescence (Supplementary Fig. S1c, d).

### Biophysical analyses

Differential scanning fluorimetry (DSF) and surface plasmon resonance (SPR) assays were used to assess the physical interaction between the top 5 CTLA-4–inhibiting compounds and abatacept, a recombinant protein that comprises the human CTLA-4 ECD fused to the human immunoglobulin G1 crystallizable fragment (Fc). When a small-molecule ligand preferentially binds to the native form of a protein, the ligand stabilizes the protein, forming a complex with a melting temperature higher than that of the unbound protein. We performed the DSF assay with abatacept and 7 compounds at concentrations of 1 to 100 μM and found 5 compounds (A6, A9, B10, D7, and D11) that significantly increased the melting temperature of abatacept (Fig. 1g, h). SPR is based on the resonant oscillation of conduction electrons at the interface between material with negative and positive permittivity; the electrons are stimulated by incident light. In the SPR analysis, abatacept was immobilized on a CM5 sensor chip surface with an immobilization level of about 15,000 relative units. Affinity analysis was carried out using a Biacore T200 instrument. Analyte D11, at concentrations of 100, 50, 25, 12.5, 6.25, 3.125, 1.563, and 0 μM, was injected onto the sensor surface for interaction with abatacept. A steady-state fitting method or a 2:2 binding model was used to measure the binding affinity and/or kinetics. Immobilized CTLA-4 on CM5 chips was found to bind D11 and A9 with dissociation constants (K_D_s) of 45.0 μM and 41.6 μM, respectively (Fig. 1i). D7 and B10 bound to abatacept with a lower affinity, and A6 did not bind to abatacept at all (Table 1, Supplementary Fig. S2a–c).

### Ex vivo validation

**Fig. 2:**
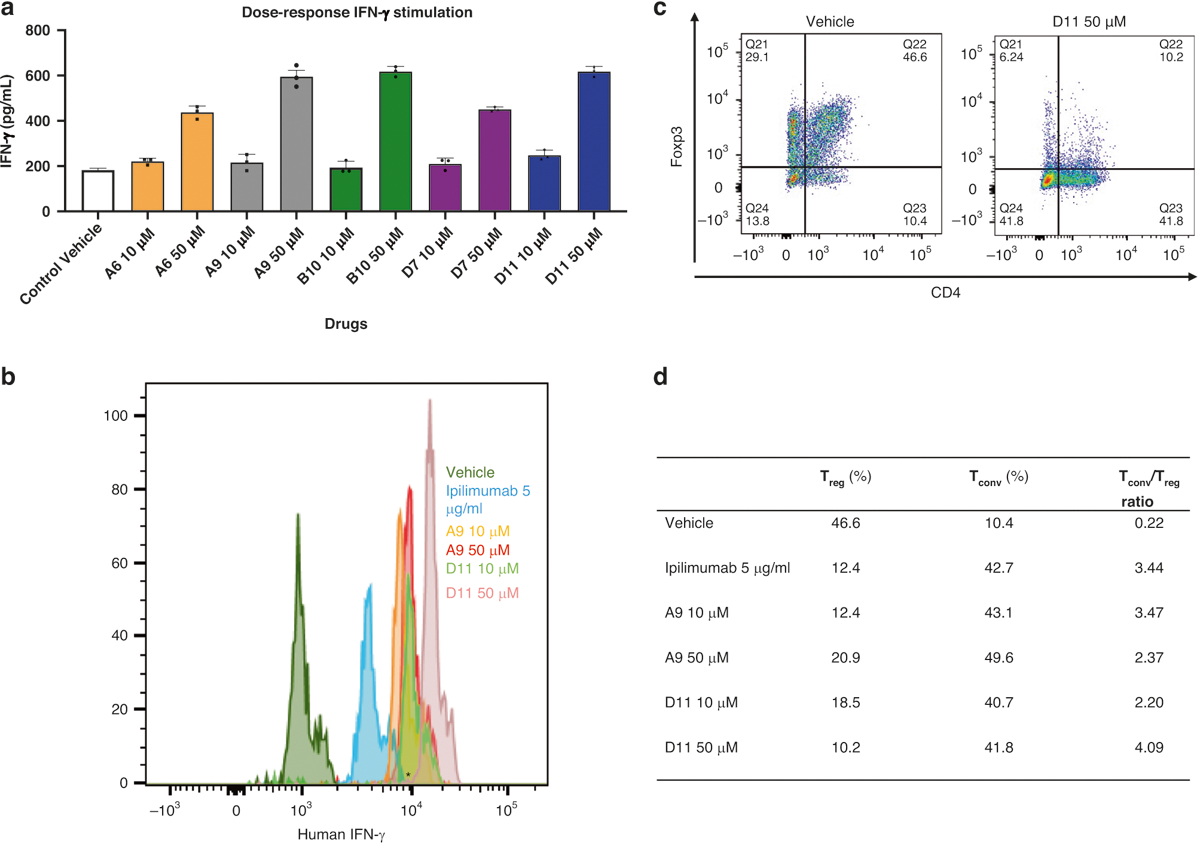
IFN-ϒ production in T cells and T conventional /T regulatory cells treated with CTLA-4 inhibitors. **a** Mouse IFN-ϒ. Tumor-infiltrating lymphocytes from C57BL/6 mice and MC38 cells were cocultured with the top 5 compounds at 2 doses (10 μM, 50 μM). The IFN- ϒ value was measured with AlphaLISA. **b** Human IFN-ϒ stimulation. Dendritic cells and allogenic T-cells from human peripheral blood mononuclear cells were cocultured with the compounds at 2 doses (10 μM and 50 μM), and IFN-ϒ–positive cells were detected with flow cytometry. **c** The T_Conv_:T_reg_ ratio in representative populations. The T_Conv_:T_reg_ ratio was also determined with flow cytometry. **d** T_Conv_ and T_reg_ individual percentages and ratios. The table summarizes the percentages of T_conv_ and T_reg_ cells in cocultured dendritic cells and peripheral blood mononuclear cells treated with A9 and D11 (10 μM and 50 μM) compared to controls (*n* = 3). AlphaLISA amplified luminescent proximity homogeneous LISA, CTLA-4 cytotoxic T-lymphocyte–associated protein 4, Foxp3 forkhead box p3, IFN-γ interferon γ, T_conv_ T conventional, T_reg_, T regulatory.

We examined the ability of these 5 anti-CTLA-4 compounds to stimulate interferon-γ (IFN-γ) production in mouse tumor-infiltrating lymphocytes (TILs). MC38 tumors grown in C57BL/6 mice were extracted to obtain CD45^+^ TILs. Upon incubating the TILs with MC38 tumor cells for 2 days, we found that each of the 5 compounds markedly increased IFN-γ release at 50-μM doses but not at 10-μM doses (Fig. 2a). Ipilimumab also substantially stimulated IFN-γ production in mouse TILs at 3-μg/ml doses but not at 1-μg/ml doses.

CD80 and CD86 on dendritic cells (DCs) interact with CD28 or CTLA-4 on T cells to regulate T-cell activation [40, 41]. We cultured human DCs with allogeneic peripheral blood mononuclear cells (PBMCs) for 6 days in the presence or absence of A9, D11, or ipilimumab and then determined IFN-γ production and the ratio of CD8^+^ T-conventional (T_conv_) cells to CD4^+^/CD25^+^/forkhead box p3 (Foxp3)^+^ regulatory T (T_reg_) cells using flow cytometry. In this analysis, there were more IFN-γ^+^ T_conv_ cells in cocultured cells treated with D11 at 50 μM than in those treated with A9 at 50 μM or with ipilimumab at 5 μg/mL (Fig. 2b, c). In addition, the T_conv_:T_reg_ ratio increased more significantly in cells treated with D11 at 50 μM than in those treated with A9 at 50 μM or ipilimumab at 5 μg/mL (Fig. 2d and Supplementary Fig. S3a, b). These data indicate that D11 induces the skewing of T helper type 1 cell numbers in the presence of tumor cells or DCs.

### MC38 Syngeneic Tumors Treated by Anti-CTLA-4 Compounds

**Fig. 3:**
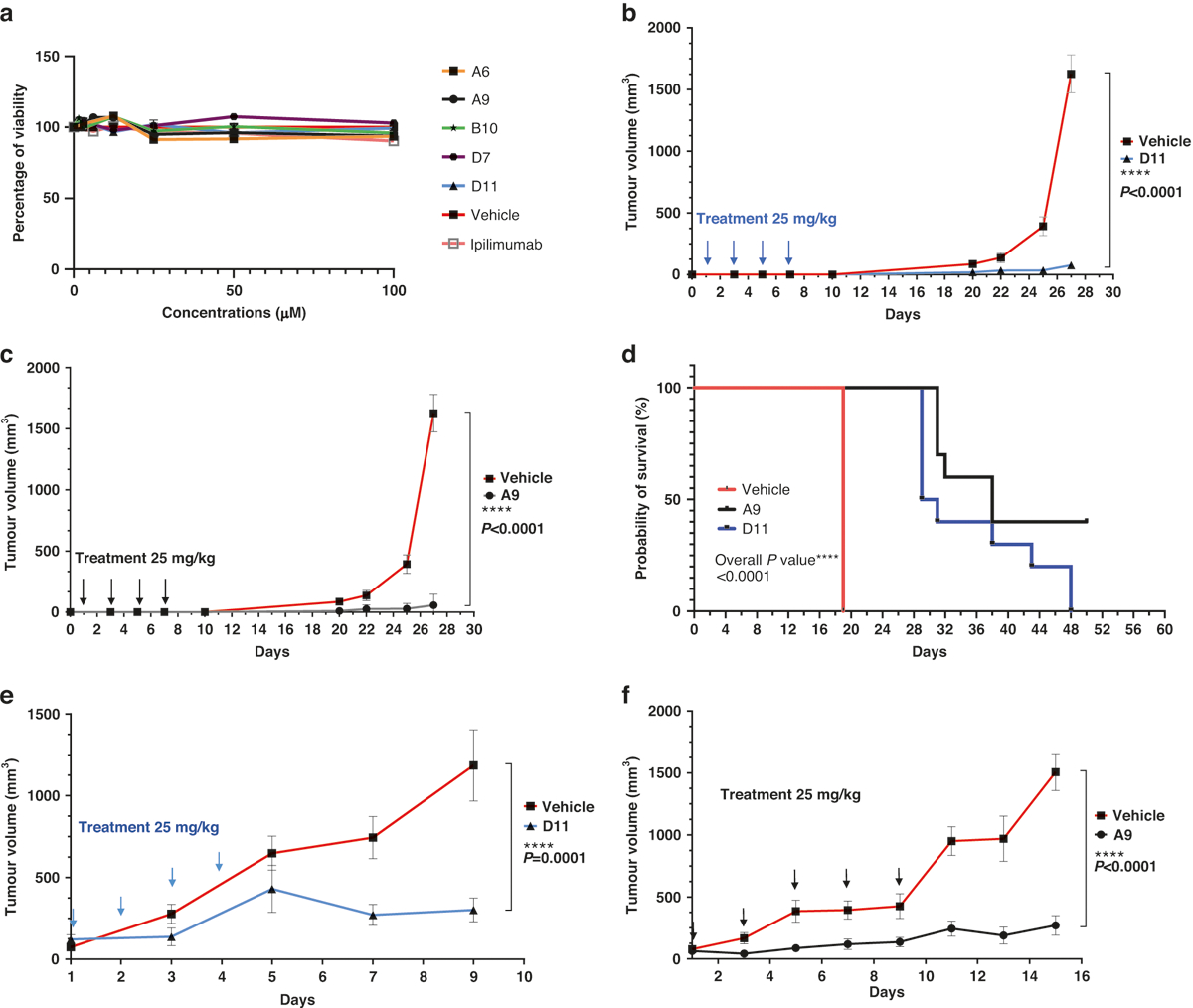
Tumor inhibition in C57BL/6 mice. **a** In vitro cell viability assay. The compounds did not affect the viability of the MC38 cells directly. The MC38 cells were treated with the compounds for 48 h. The highest concentration for ipilimumab was 3 μg/mL (*n* = 4). **b**, **c** Tumor prevention. D11 and A9 prevented MC38 tumor growth. Mice were intraperitoneally injected with the compound D11 (*n* = 10 mice) or A9 (*n* = 10 mice) or the vehicle control (phosphate-buffered saline; *n* = 5 mice) before the tumors were established. **d** Kaplan-Meier survival curves. **e**, **f** Inhibition of MC38 tumors. Mice treated with D11 or A9 had significantly longer survival than mice treated with the vehicle. Treatments (*n* = 5 for D11, *n* = 7 for A9, and *n* = 7 for the vehicle) were started after the tumors were established, and the tumor volumes reached approximately 100 mm^3^. Error bars represent the standard error of the mean.

We treated MC38 cells cultured in petri dishes with the 5 anti-CTLA-4 compounds (100 μM) for 48 h and found that treatment did not significantly affect the cells’ viability (Fig. 3a). We next subcutaneously inoculated C57BL/6 mice with 200,000 MC38 cells before treating them with 4 200-μg doses (the equivalent of 25 mg/kg) of A9 or D11 per animal via intraperitoneal injection every other day. In this prophylactic setting, in which treatments started 1 day after tumor cell inoculation, A9 and D11 drastically inhibited tumor growth and prolonged animal survival (Fig. 3b–d). The numbers of tumor-infiltrating T_reg_ cells were reduced by A9 and D11 treatment (Supplementary Fig. S3c, d). Four of 10 mice in the A9 group completely rejected the tumors and were rechallenged with 200,000 MC38 cells. Tumor growth was slower in the rechallenged mice than in the control group (Supplementary Fig. S4a), suggesting that the A9-treated mice had limited-memory immunity. No animals treated with D11 exhibited a complete response (Supplementary Fig. S4b). In the therapeutic setting, in which treatments started when tumor volumes reached approximately 100mm^3^, both A9 and D11 significantly reduced tumor growth (Fig. 3e, f and Supplementary Fig. S4c, d).

**Fig. 4:**
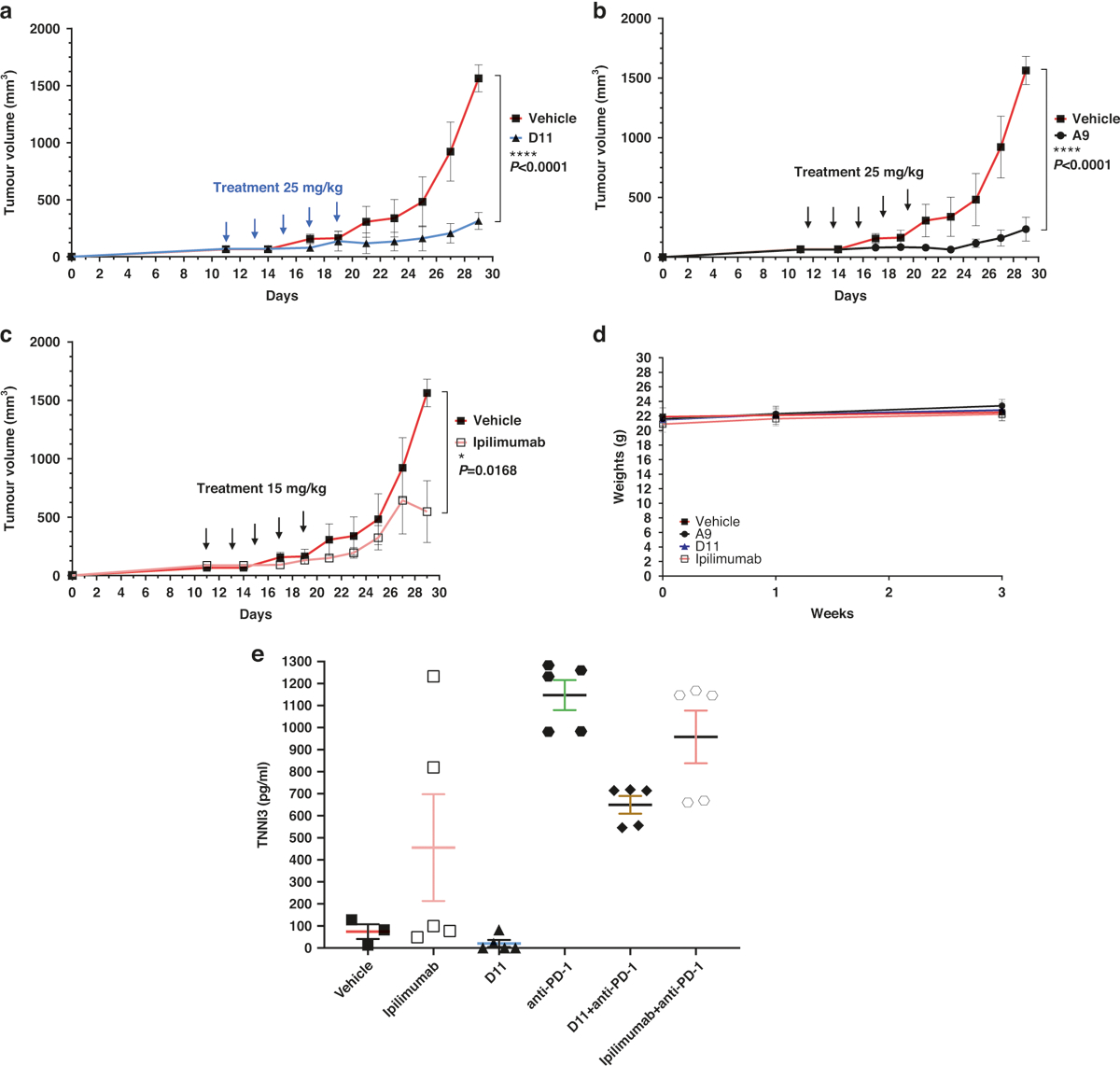
Tumor inhibition in hCTLA-4 mice. **a**–**c** Tumor inhibition. D11, A9, and ipilimumab inhibited tumors in established syngeneic mouse models expressing transgenic hCTLA-4 protein. Treatments (*n* = 5 for D11, *n* = 5 for A9, *n* = 5 for ipilimumab, and *n* = 3 for the phosphate-buffered saline vehicle) were started after the tumors were established, and the tumor volumes reached approximately 100 mm^3^. **d** Mouse body weights after the initiation of treatment. The respective treatments did not lead to significant weight loss. **e** Cardiac troponin type III (TNNI3) toxicity was not observed in mice treated with the compounds alone. Error bars represent the standard error of the mean. hCTLA-4 humanized CTLA-4, PD-1 programmed cell death protein-1.

Given that the ECDs of mouse and human CTLA-4 proteins are only 74% identical (although the intracellular domains are 100% identical), we next evaluated A9 and D11 using humanized CTLA-4 (hCTLA-4) mice [42]. In hCTLA-4 mice, the ECD of the mouse *Ctla-4* gene has been replaced with the human counterpart, yet the resulting protein still interacts effectively with mouse CD80 and CD86 [42]. In this model, the A9 and D11 regimens substantially inhibited the development of MC38 tumors (Fig. 4a–c). A9 and D11 also appeared to have superior tumor-inhibition activity in hCTLA-4 mice compared with non-humanized C57BL/6 mice (Supplementary Fig. S5). In addition, when we weighed the A9- or D11-treated hCTLA-4 mice that had been inoculated with MC38 cells, we found no significant weight loss in the treated compared with the control mice (Fig. 4d). Next, we measured the mice’s levels of serum cardiac troponin I type 3 (TNNI3), a marker associated with ipilimumab-induced irAEs [43]. We found elevated serum TNNI3 levels in mice treated with ipilimumab, anti-PD-1, or anti-PD-1 plus D11, but not in mice treated with D11 alone (Fig. 4e). It is unclear why D11 monotherapy appeared to have a protective effect in comparison with anti-PD-1 alone. We also treated 2 human cells lines, HCT116 and MDA-MB231, with D11 and 5-fluorouracil, an antimetabolite drug that is widely used for the treatment of colorectal cancer. Compared with 5-fluorouracil, D11 was significantly less toxic to the cancer cells (Supplementary Fig. S6). Thus, it is likely that D11 is weakly toxic to tumor cells and that its antitumor effects are largely due to its regulation of the immune response.

### Single-cell sequencing

**Fig. 5:**
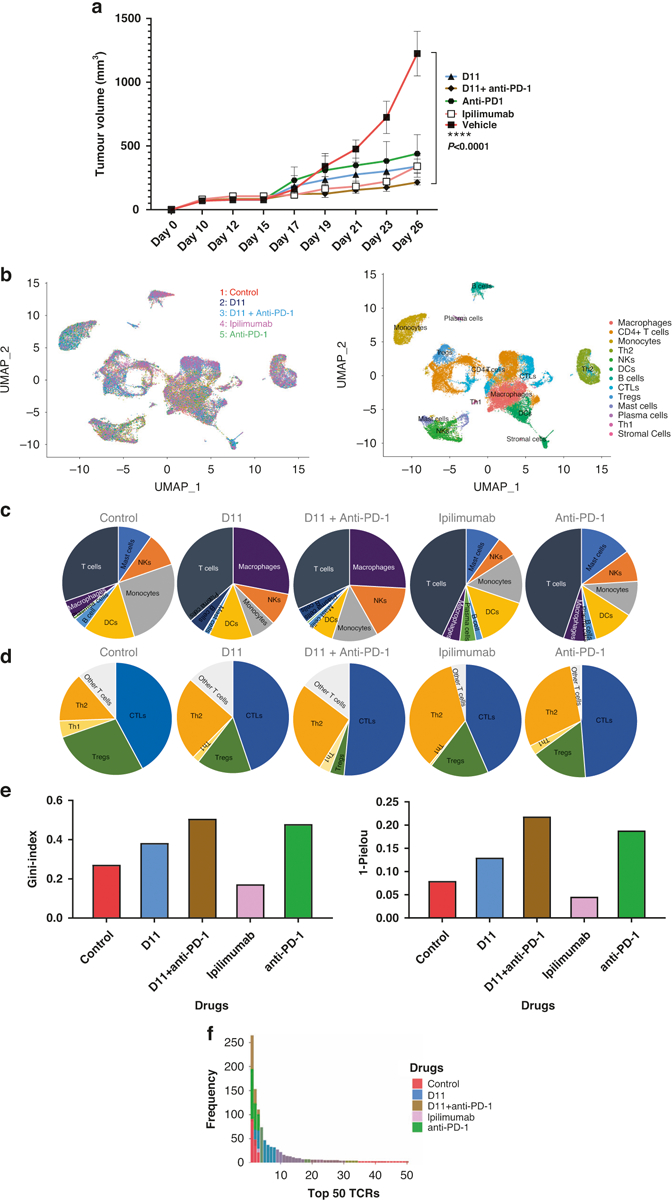
Single-cell sequencing. **a** Tumor inhibition in mice. D11, ipilimumab, anti-PD-1, and D11 plus anti-PD-1 inhibited tumors in established syngeneic mouse models expressing transgenic humanized CTLA-4 protein. Treatments (*n* = 5 for D11, *n* = 4 for anti-PD-1, *n* = 5 for D11 plus anti-PD-1, *n* = 5 for ipilimumab, and *n* = 4 for the phosphate-buffered saline vehicle) were started after the tumors were established, and the tumor volumes reached approximately 100 mm^3^. **b** Single-cell RNA sequencing integration and clustering. The data from the 5 different treatment groups were integrated using Seurat (V.4.0.3) in R to account for the batch effect for the downstream analyses. Thirteen immune-related clusters were identified. **c**, **d** Different immune and T-cell population numbers are presented in the charts for each treatment group. **e** Uneven distribution of T-cell receptors (TCRs) in therapies. D11, anti-PD-1, and the combination of these 2 treatments increased the uneven distribution of TCRs. Ipilimumab decreased the clonality of the TCR repertoire, whereas the other 3 treatments increased it. **f** The frequency of the top 50 TCRs increased with the use of D11 plus anti-PD-1. CTL cytotoxic T cell, CTLA-4 cytotoxic T-lymphocyte–associated protein 4, DC dendritic cell, NK natural killer, PD-1 programmed cell death protein-1, PD-L1 programmed death-ligand 1, and Treg T regulatory cell.

Using another cohort of hCTLA-4 mice inoculated with MC38 cells, we treated animals (*n* = 5 per group) with ipilimumab, anti-PD-1, anti-PD-1 plus D11, D11 alone, or vehicle in the same dosages as used previously. Some animals’ tumors were collected approximately 10 days after the last treatment dose, whereas for the remaining animals, the tumors were allowed to grow (Supplementary Fig. S7). We found that treatment with D11 plus anti-PD-1 had the best therapeutic efficacy (Fig. 5a). Single-cell suspensions from tumors were sorted to obtain CD45^+^ immune cells, which were then subjected to single-cell RNA sequencing (scRNAseq) and TCR sequencing. Next, the immune cell populations were compared across the samples (Fig. 5b, c). We found that all treatments increased the numbers of cytotoxic T lymphocytes (with anti-PD-1 treatment increasing them the most), whereas D11 plus anti-PD-1 treatment decreased the number of T_reg_ cells the most (Fig. 5d). An unexpected finding was the significant increase in tumor-infiltrating macrophages associated with D11 monotherapy or D11 plus anti-PD-1 treatment. The macrophage populations were only moderately affected by ipilimumab or anti-PD-1, suggesting that D11 was the underlying cause for the increase in tumor-infiltrating macrophages. Another interesting observation from the scRNAseq analysis was that the differential expression of membrane-type 1 matrix metalloproteinase, which is known for its role as a key regulator in cell migration in tissues, was associated with D11 treatment. This finding could lead to a possible new mechanism of action, besides macrophage recruitment, to drive the search for anticancer compounds.

We used the Gini and the 1-Pielou indexes to determine the clonotype distributions and the clonality of the TCR repertoire from the TCR sequencing data. The Gini index data showed a substantial and statistically significant shift toward decreased clonality (i.e., toward situations in which the relative abundance of various clonotypes is substantially different) in the TCRs from tumor-infiltrating T cells from ipilimumab-treated compared with control-treated tumors (Fig. 5e). D11, anti-PD-1, and D11 plus anti-PD-1 combination treatment increased the uneven distribution of TCRs. The 1-Pielou index also showed that ipilimumab decreased the clonality of the TCRs, whereas the other 3 treatments increased it (Fig. 5e). The TCR α and β chains possess 3 hypervariable regions termed complementarity-determining regions (CDR1, 2, and 3). CDR3 plays a dominant role in recognizing processed antigen peptides. Figure 5f shows the frequencies of the top 50 TCRs from each group, and Table 2 lists the sequences of the CDR3s of the top 5 TCR α and β clones. These data indicate that targeting CTLA-4 using ipilimumab (an antibody) and D11 (a small molecule) may have different impacts on tumor-infiltrating T cells and macrophages.

### Optimization of lead anti-CTLA-4 compounds

**Fig. 6:**
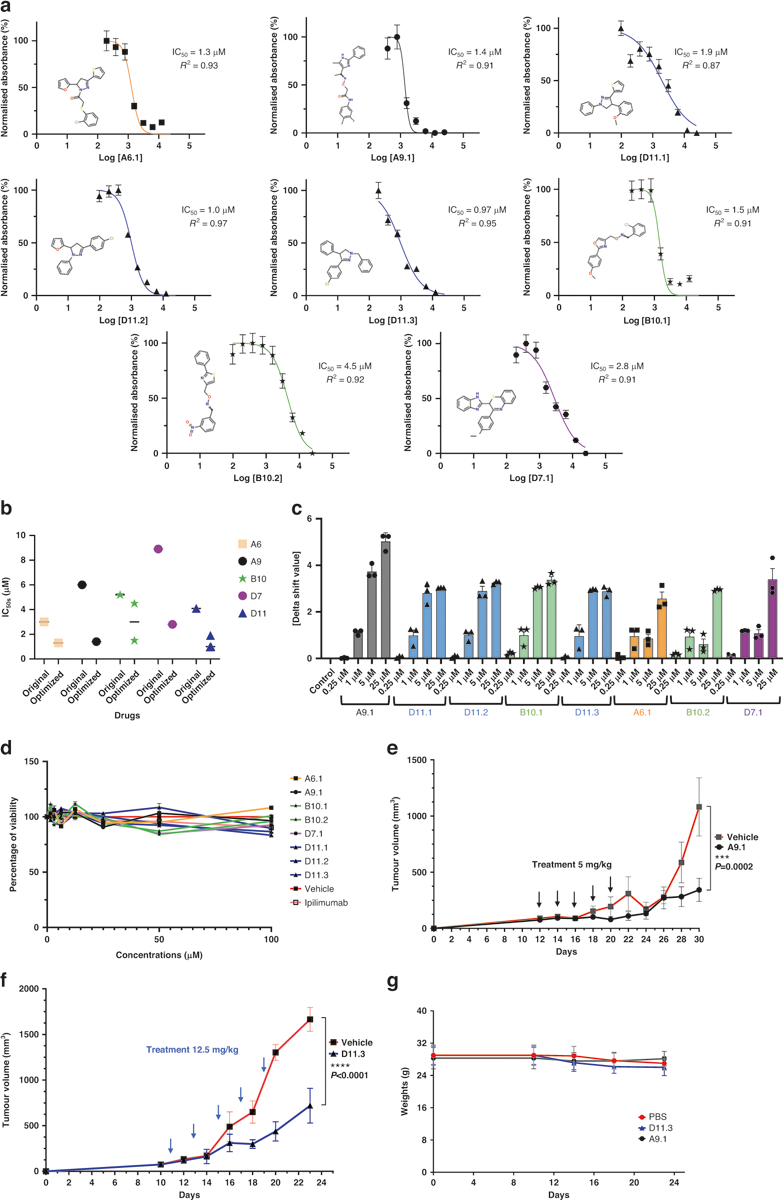
Optimization of CTLA-4-targeting compounds. **a** AlphaLISA. IC_50_ values were measured as the reduction in 615 nm luminescence (color-coded panels: the optimized lead compounds were A6.1, A9.1, D11.1, D11.2, D11.3, B10.1, B10.2, and D7.1). **b** IC_50_ comparison between optimized and parental compounds. **c** PTS. Optimized dose-response results (0.25 μM, 1 μM, 5 μM, and 25 μM) are illustrated. **d** In vitro cell viability assay. The compounds did not affect MC38 viability directly. MC38 cells were treated with compounds for 48 h. The highest concentration for ipilimumab was 3 μg/ml (*n* = 4). **e** A9.1 inhibited tumors in established syngeneic mouse models expressing transgenic humanized CTLA-4 protein. Treatments (*n* = 11 for A9.1, *n* = 10 for the phosphate-buffered saline vehicle) were started after the tumors were established and the tumor volumes reached approximately 100 mm^3^. **f** D11.3 inhibited tumors in established syngeneic mouse models expressing transgenic humanized CTLA-4 protein. Treatments (*n* = 5 for D11.3, *n* = 5 for the vehicle) were started after the tumors were established, and the tumor volumes reached approximately 100 mm^3^. **g** The respective treatments did not lead to significant weight loss in the mice. Error bars represent the standard error of the mean. AlphaLISA amplified luminescent proximity homogeneous LISA, CTLA-4 cytotoxic T-lymphocyte–associated protein 4, IC_50_ half-maximal inhibitory concentration, PTS protein thermal shift.

We next investigated analogs of the 5 anti-CTLA-4 compounds for possible refinement. We acquired 19 analogs of A9, 18 of D11, 16 of A6, 12 of D7, and 18 of B10 (Supplementary Table S1). We screened the analogs using the AlphaLISA assay and, for each compound, found 1 or more optimized analogs with an improved IC_50_ value (A6.1, A9.1, D11.1, D11.2, D11.3, B10.1, B10.2, and D7.1; Fig. 6a, b). Like the primary hits, the optimized compounds did not reduce membrane expression levels of CTLA-4 in Jurkat cells overexpressing exogenous CTLA-4 after 45 min or 24 h minutes of coincubation, when compared to ipilimumab (Supplementary Fig. S8a, b). We performed a DSF assay and found that the optimized analogs significantly increased the abatacept melting temperature at a lower dose (1–25 μM; Fig. 6c) than that of their parental compounds (Fig. 2b). Similarly, the optimized compounds did not directly kill MC38 tumor cells in vitro (Fig. 6d). When we treated hCTLA-4 mice inoculated with MC38 cells therapeutically, we delivered the A9.1 and D11.3 compounds at doses of approximately 5 mg/kg or 12.5 mg/kg per animal per injection for 5 doses. Even at these lower dosages, A9.1 and D11.3 markedly reduced tumor growth (Fig. 6e, f and Supplementary Fig. S8c, d) without affecting the body weight of the animals (Fig. 6g).

## DISCUSSION

The field of cancer immunotherapy has advanced rapidly since the first approvals of monoclonal antibodies targeting CTLA-4 and PD-1. Ipilimumab was approved by the FDA in 2011, yet the second anti-CTLA-4 monoclonal antibody, tremelimumab, was not approved until 2022 for the treatment of unresectable hepatocellular carcinoma, and then only in combination with the anti-PD-L1 antibody duralimumab. In less than 10 years, 7 anti-PD-1 or anti-PD-L1 antibodies have been approved by the FDA. Except in the treatment of melanoma, ipilimumab is given in combination with nivolumab, an anti-PD-1 antibody. Cancer regimens with anti-CTLA-4 monotherapy have largely been replaced by the combination of anti-CTLA-4 and anti-PD-1/PD-L1 antibodies. The major goals of anti-CTLA-4 antibody development are to find antibodies with higher affinities for this protein, longer half-lives, fewer irAEs, modifiable doses and treatment schedules, and increased risk-to-benefit ratios. It has been demonstrated that tremelimumab, when engineered into a new, pH-sensitive antibody resistant to degradation, inhibits but does not degrade CTLA-4 and dramatically attenuates irAEs in mice [16]. A handful of novel antibodies targeting CTLA-4 are under development, and these antibodies differ from ipilimumab in regard to their epitopes, half-lives, isotypes, Fc receptor-related functionality, pH sensitivity, and/or binding affinity.

Among the strengths of AI in the discovery of small molecules are the reduced times and costs of physical testing. AI performs high-fidelity molecular similarity testing entirely *in silico*. When combined with systems such as AlphaFold, AI can predict the 3-dimensional structures of target proteins from their amino acid sequences. In addition, the strength of some AI systems is their ability to bypass the physical-chemical testing of drugs [44]. Several online tools, including LimTox, pkCSM, admetSAR, and Toxtree, can be used to predict toxicity [45]. Additionally, AI can be used to predict solubility [46], dissociation constants, [45, 47] and bioactivity [48], and instead of screening large libraries for candidates, AI can generate new drugs from scratch [49]. Also, the AI ranking of drug efficiency is incredibly powerful. Once the promising drugs have been discovered, AI outperforms other technologies in ranking the top drugs in terms of their physical and chemical properties, such as the power of the drug to inhibit its target [50]. Finally, AI has an unprecedented ability to predict which synthesis pathway should be implemented to produce hypothetical drugs, which then leads to suggested chemical modifications to further enhance the drug-manufacturing process [49]. For these reasons, we used AI in this study to identify anti-CTLA-4 drugs, and we believe that it can also be used to help speed up the discovery of other small-molecule inhibitors of cancer.

In this study, we discovered and optimized small molecules targeting CTLA-4 using deep convolutional neural network-based AI screening and experimental validation. We found several optimized compounds which bound to CTLA-4 at low micromolar concentrations, as measured by AlphaLISA and SPR. Two compounds, A9 and D11, elicited an immune response and inhibited tumor development in MC38 murine syngeneic mouse models at doses of 25 mg/kg. We evaluated A9, D11, and their analogs using a humanized mouse line, in which the ECD of mouse CTLA-4 was replaced by the human version. A9 and D11 inhibited tumor development in hCTLA-4 mice at doses of 25 mg/kg, and their analogs (A9.1 and D11.3) did so at even lower doses (5–12.5 mg/kg). We note that, in a phase II study, an anti-PD-1 compound, INCB086550, inhibited tumor development in PD-1–humanized mice at doses of 2 to 200 mg/kg [51]. These data indicate that anti-CTLA-4 small molecules effectively inhibit tumors at dosages comparable to those at which reported anti-PD-1 compounds inhibit tumors. We also found that D11 increases the clonality of TCRs better than ipilimumab. This intriguing finding requires more follow-up experimentation to better understand its significance.

Interestingly, D11 therapy significantly increased the overall macrophage population.

Among myelomonocytic cells, macrophages possess a dual nature in the context of cancer in that they display both beneficial and detrimental effects that can be attributed to their ability to adapt to different environmental signals [52–54]. Macrophages exhibit the potential to eliminate cancer cells through various mechanisms such as phagocytosis, antibody-mediated cytotoxi-city, and the direct killing of tumor cells. Additionally, they can induce damage to tumor blood vessels and promote tumor necrosis [54]. Furthermore, macrophages can activate both innate and adaptive immune responses, thereby enhancing the body’s defense against tumors [52–55]. On the other hand, macrophages can play a role in cancer progression and metastasis in established tumors. They contribute to these processes through several mechanisms, including supporting the survival and growth of cancer cells, promoting the formation of new blood vessels (angiogenesis), and suppressing the activities of innate and adaptive immune cells [56].

Considering the aforementioned contrasting roles of macrophages in cancer, activating them has emerged as a potential therapeutic strategy. By appropriately activating macrophages, it is possible to harness their tumor-killing capabilities and exploit their interactions with other components of the immune system. This approach holds promise for developing effective cancer therapies that leverage the multifaceted functions of macrophages while minimizing their tumor-promoting effects. In our opinion, our lead compound D11 properly activates macrophages to promote the T-cell–mediated cytotoxic death of cancer cells, and in some cases, this activation may lead to prophylactic and therapeutic tumor necrosis by activating an immune response against cancer cells. A review by Mantovani et al. [56] provides a more thorough discussion of the dual role of macrophages in cancer.

The study had several limitations. First, even after optimization, the IC_50_ or K_D_ values of the compounds binding to CTLA-4 only reached the 1-μM range, the starting point in medicinal chemistry for small-molecule inhibitors. The K_D_ values from SPR were larger than the values for IC_50_ from AlphaLISA. We note that the assays measured the biochemical, cellular, and antitumor activities of the compounds differently (Table 3). AlphaLISA measures the interaction between human CTLA-4 and human CD80, whereas DSF and SPR depend on abatacept (the hCTLA-4-ECD-Fc). It is possible that the compounds bound the CTLA-4-CD80 complex better than abatacept; if so, it is likely because we prioritized the CTLA-4-CD80 complex in the first step of experimental validations. This may also explain why A6 disrupted the CTLA-4/CD80 interaction but did not bind CTLA-4. Second, none of the compounds obeyed the rule of 3 [57], although they complied with “the Lipinski Rule of 5” (RO5, including the logarithm of the partition coefficient between octanol and water [logP] ≤5, molecular weight <500 g/mol, and ≤5 and ≤10 hydrogen-bond donors and acceptors, respectively) [58, 59]. The RO5 has been reduced to the rule of 3 for defining lead-like compounds: logP ≤3, molecular weight <300 g/mol, and ≤ 3 hydrogen-bond donors and ≤3 hydrogen-bond acceptors. We note that the RO5 is highly influential in drug development, but only about 50% of orally administered new chemicals obey it [60]. Third, antibodies have superior specificities and half-lives compared with compounds, mitigating the absolute need for small molecules to block immune checkpoints. However, compared with antibodies, small molecules potentially have the following advantages: (1) oral delivery, (2) lower manufacturing costs, (3) higher stability at ambient temperatures, (4) greater distribution within tumor tissues (small molecules can cross the blood-brain barrier to treat brain tumors), and (5) mechanistic differences. Finally, the study of the toxicities of the discovered anti-CTLA-4 compounds was limited, as the compounds are not yet ready for human studies. Once we obtain the final candidate drugs for clinical trials, we will initiate a complete absorption, distribution, metabolism, excretion, and toxicity study.

Although there are algorithms available that may not be as effective as AtomNet in predicting the binding of small molecules to CTLA-4, it could be worthwhile to explore these alternatives to compare their screening results. However, this would be outside the scope of this study, as our research focused on validating the discovery of CTLA-4–inhibiting drugs using the AtomNet method. Our primary focus was on validating the newly discovered drugs through rigorous laboratory experiments conducted both in vitro and in vivo. While we acknowledge that there are various AI-based tools available for predicting physicochemical properties, we did not explore them extensively in our study. It is important to note that each pharmaceutical company may have its own proprietary AI drug discovery method, which further adds to the complexity of comparing different approaches. Unfortunately, we are not aware of any publicly available methods that can achieve screening results comparable to those of AtomNet in terms of scale. For a more comprehensive review of AI drug discovery methods, we recommend referring the review by Paul et al. [44], which provides valuable insights into the current landscape of AI-based drug discovery. Like AtomNet, these methods are not accessible to the wider scientific community.

In conclusion, our AI-driven drug discovery procedure generated several compounds that bind to CTLA-4 without degrading CTLA-4 protein on the cell surface. These compounds impaired the CTLA-4/CD80 interaction and inhibited tumor growth in syngeneic and hCTLA-4 mice. In mice, 1 compound, D11, had low toxicity alone or in combination with anti-PD-1 antibodies. D11 and ipilimumab appear to employ different mechanisms of tumor inhibition, although this observation requires further study and characterization. Given that the combination of inhibitors against both CTLA-4 and PD-1 provides superior outcomes than either agent alone [61], anti-CTLA-4 small molecules represent a compelling alternative to anti-CTLA-4 antibodies and may make immune checkpoint blockades more accessible and affordable while minimizing side effects.

## MATERIALS AND METHODS

### Virtual high-throughput screening and optimization with the AtomNet technology

The AtomNet algorithm is a proprietary technology of Atomwise. It employs deep convolutional neural networks analogous to the ones used for computer vision, pattern recognition, and image processing techniques but tailored for structure-based drug design and discovery. Unlike more traditional approaches rooted in classical mechanics and quantum chemistry, in which explicit terms like “van der Waals”, “electrostatics”, “H-bonding”, and “hydrophobic interactions” are parameterized (often with human intervention) and used for molecular modeling, the AtomNet technology systematically learns optimal model parameters for predicting the binding of small molecules to proteins, and it does so in a robust and efficient manner [37, 62–66]. The AtomNet technology has been highly successful as a means of discovering small compounds across different branches of medicine [62–64, 67–70].

To target CTLA-4, we screened around 10 million commercially available compounds from Mcule.com. Seventy-two diverse, drug-like molecules were selected and procured for use in experimental validations. For hit compound optimization, we performed analog-by-catalog screens by searching a chemically purchasable space for additional bioactive compounds chemically analogous to the hits. The space contains the 1000 nearest neighbors of the hits from the Mcule library, as determined using molecular fingerprint similarity [71]. Additional analogs were identified using RDKit or FTrees substructure searches [72]. We used an AtomNet regression model trained to predict quantitative bioactivity (e.g., IC_50_ or K_D_) to score and rank the analogs. A set of compounds per hit, determined using the analog space of an initial hit, were then obtained based on the similarity to the original family of compounds from the initial screening and the top testing scores from the AtomNet model. Around 12 to 19 compounds were successfully synthesized with over 95% purity.

### CTLA-4/CD80 AlphaLISA

The efficacy of anti-CTLA-4 compounds in binding to and inhibiting the interaction between CTLA-4 and CD80 was first evaluated using the CTLA4-CD80 AlphaLISA Binding Kit (PerkinElmer, AL3046). In this assay, a biotinylated CD80 binds to streptavidin-coated alpha donor beads, while His-tagged CTLA-4 is captured by anti-His AlphaLISA acceptor beads. When CD80 binds to CTLA-4, the donor beads and acceptor beads come into close proximity. The excitation of the donor beads causes the release of singlet oxygen molecules, triggering a cascade of energy transfer in the acceptor beads and resulting in a sharp peak of light emission at 615 nm. The compounds or ipilimumab were incubated with the reaction mixture for 90 min at room temperature before AlphaLISA luminescence was measured on a BMG (CLARIOstar) alpha-enabled microplate reader.

### DSF

DSF is an assay based on protein thermal stability upon ligand binding. Abatacept is a fusion protein composed of the Fc region of the immunoglobulin G_1_ fused to the extracellular domain of CTLA-4. Serial dilutions of the compounds (50 μM, 5 μM, and 1 μM), the carboxyrhodamine reference dye, and 0.1 μg/mL of abatacept were incubated in a 20-μl reaction. Thermal shift assays were performed using the 96 Real-Time PCR System QuantStudio 7 Pro (ThermoFisher) melting curve program with a temperature increment of 0.2 °C and a temperature range of 25° to 95 °C. The melting curve program was set on a continuous mode with the following 2 ramp rates: (1) 1.6° C/s at 25 °C for 2 min and (2) 0.05° C/s at 99° C for 2 min. Protein Thermal Shift software (ThermoScientific) was used to estimate the midpoint temperatures of the protein-unfolding transitions on the basis of the positive derivative of fluorescence decrease as the DNA becomes single-stranded derivative of Relative Fluorescence Unit / derivative Temperature (d(RFU)/dT) curves.

### SPR

Biophysical binding affinity measurements of anti-CTLA-4 compounds to abatacept using an SPR experiment were performed using a Biacore T200 or 8 K instrument at Creative Biolabs (Shirley, NY). Abatacept was directly immobilized on the CM5 chip using an amine coupling kit. Fitting of the sensorgram data was used to calculate the K_D_s from the rate of association (K_on_) and dissociation (K_off_) binding parameters.

### Mouse and human cell responses to treatment

The ability of compounds to be immunostimulatory was measured by coculturing mouse-derived TILs with MC38 tumors grown in C57BL/6 mice. Tumors were digested using a Tumor Dissociation Kit (Miltenyi Biotec, 130-095-929). After cell isolation, TILs were extracted with a MagniSort Mouse CD45 isolation kit protocol (Invitrogen, 8802-6865-74). TILs were cocultured with MC38 cells for 48 h, and the expression levels of IFN-γ were detected with the AlphaLISA Mouse IFN-γ Detection Kit (PerkinElmer, AL501C). Human DCs and T cells from different patients were prepared and treated as described [73]. For MC38 and human cancer cells treated with compounds, the CellTiter-Glo Luminescent Cell Viability Assay (Promega, G7570) was used to measure the viability.

### Flow cytometry

Flow cytometry was used to measure subcellular populations in tumors. All flow cytometry analyses were performed using either an LSR II flow cytometer (BD Biosciences) or a Northern Lights-3000 flow cytometer (Cytek) and analyzed with FlowJo (Treestar, Inc.). All flow analyses were representative of at least 3 independent experiments using anti-human CD proteins 4, 8, and 25 (CD4, 116003; CD8, 100707; and CD25, 102011); Foxp3 (126409); and IFN-γ (506506) flow antibodies (Biolegend). Antimouse and antihuman CTLA-4 antibodies were purchased from BioLegend (106313) and Invitrogen (46-1529-42).

### Experimental animals

C57BL/6 mice were purchased from the Charles River Laboratories. In hCTLA-4 mice, the exons encoding for the ECD and transmembrane domains of murine CTLA-4 were replaced with the human versions [42]. All mice were maintained at the Research Animal Facility of Baylor College of Medicine, and all studies were conducted under an animal protocol approved by the Baylor College of Medicine Institutional Animal Care and Use Committee. Mice were challenged with 0.5 million MC38 colorectal cancer cells passaged the previous day at 75% confluence. For the prophylactic model, ipilimumab or small-molecule treatment began 1 day after tumor cell inoculation. For the therapeutic model, ipilimumab or small-molecule treatment was initiated 7 to 17 days after the injection of tumor cells, depending upon when the tumors reached an average size of approximately 100 mm^3^. Tumor volumes were calculated with the formula volume = (width^2^ × length)/2. Measurements were recorded every 2 to 3 days. For the histologic analyses, the internal organs, including the livers, kidneys, lungs, colons, and salivary glands, were fixed in 10% paraformal-dehyde for at least 24 h, cut from paraffin blocks in 2- to 5-μM sections using a Microm HM 315 microtome, and mounted on SuperFrost Plus slides (Roth). Slides were stained with hematoxylin and eosin (Shandon Varistain [24–4] Automatic Stainer). For the histopathologic analysis of the internal organs harvested from mice that had been treated with therapeutic compounds or control antibodies, stained sections were prepared from the formalin-fixed tissue and were assessed in a blinded fashion.

### Single-cell RNA sequencing

Tumors from CTLA-4-humanized MC38 syngeneic mice (*n* = 3–5 mice) were extracted for single-cell suspension using a gentleMACS Octo Dissociator (Miltenyi Biotec). The cells were stained with antimouse CD45 antibody (PE-Cy7, Clone 30-F11, Cat. 103114, Lot B354212) and sorted with a BD FACSAria II Cell Sorting Flow Cytometer. Library preparation of CD45^+^ sorted cells for scRNAseq was performed using a 10X Genomics Chromium Single Cell 5′ Library & Gel Bead Reagent Kit. For TCR sequencing, a Chromium Single Cell V(D)J Enrichment Kit was used. The acquired scRNAseq reads from the 10X Genomics platform were aligned to a reference genome (mm10) using Cell Ranger (V.7.1, 10X Genomics) default parameters. The reads and cells were further filtered with Seurat (V.4.0.3) [74] in R (V.4.1.0). Usually, measurements across datasets cannot be compared because of distinct factors that could influence cellular identities. Such factors, which include the sensitivity and accuracy of the methods used, can cause transcript abundance bias and lead to false negatives [75]. Therefore, we employed an unsupervised strategy to anchor datasets together to facilitate integration and comparison across single-cell-sequenced samples [76]. This method allowed accurate cross-comparisons among samples because only specific subsets of cells with the same molecular features are compared to each other. First, to overcome the batch effect, we used a diagonalized canonical correlation analysis [77] to reduce the dimensionality in the 5 treatment datasets of hCTLA-4 mice. Second, L2-normalization was applied to the canonical correlation vectors used to identify mutual nearest neighbors [78]. Third, anchors (cell pairs) were identified by searching for mutual nearest neighbors in this shared low-dimensional representation. These anchors encode for the cellular relationships across datasets; subsequent integration analyses depend on these relationships. The anchoring was successful, as we recovered matching cell states even with significant dataset differences. The canonical correlation analysis could identify effectively shared biological markers and conserved patterns of gene correlations. The Gini index, commonly used as a measure of income inequality in economics, was used to assess the inequality of clonotype distributions within a repertoire [79]. The clonality of the TCR repertoire was calculated as a 1-Pielou index value using the formula 1+ ∑_(i=1 to n)_ (p_i_*ln(p_i_))/ln(n), where p_i_ is the frequency of clone i for a sample of n unique clones; this metric was normalized to the number of unique clones and ranges from 0 to 1. The 1-Pielou index is inversely proportional to diversity, i.e., a higher clonality index indicates a more uneven repertoire structure generated by the increase of selected clones.

## Supplementary Material

Raw Data

## Figures and Tables

**Table 1.** Surface plasmon resonance showed the binding of most anti-CTLA-4 compounds to purified CTLA-4 protein.

Immobilization Level (RUs)	Compound	Compound Concentration (μM)	K_D_ (μM)	R_max_ (RUS)	Chi^2^ (RUs^2^)
9476.7	A9	100–1.563	4.16 × 10^5^	15.7	0.27
9476.7	D7	100–1.563	4.80 × 10^4^	75.5	0.011
9476.7	D11	100–1.563	4.50 × 10^5^	11.3	0.95
10354.7	A6	100–0.781	No binding		
1034.7	B10	100–0.781	2.13 × 10^4^	32.82	0.87

Chi^2^; CTLA-4, cytotoxic T-lymphocyte–associated protein 4; K_D_, dissociation constant; R_max_, maximal response when all ligand is occupied; RUs, relative units. *K*_*D*_ concentration values evaluate binding between pure CTLA-4 protein and CTLA-4 inhibitors. Binding values were measured for the top hit compounds using the surface plasmon resonance method. CTLA-4 is immobilized onto the CM5 sensor chip. The steady-state affinity model or 1:1 binding model was used to measure the binding affinity and/or kinetics.

**Table 2.** The CDR3 regions of the top 5 TCRs from each group.

Sample	% of total TCRs	TCR β	TCR *α*
	7.7%	CASSHDWG DNYAEQFF	CAMREMDYNQGKLIF, CAMREMDYNQGKLIF, CAVKGFASALTF
	4.1%	CASSQGLGEDTQYF	CAAKDYSNNRLTL
PBS	1.8%	CASSQEMGTGGREGTQYF	CAVSMSGSFNKLTF
	1.4%	CASSQTQGADTQYF	CAARDYSNNRLTL
	0.9%	CASSFPGSSYEQYF	CAASSNNNNAPRF
	11.3%	CASRTGDNYAEQFF	CAVSDGSGYNKLTF
	7.2%	CASARTGGYEQYF, CASSEGTGGTPNSDYTF	CALSDQGGSAKLIF
Anti-PD-1	5.9%	CASSLELGGLEQYF	CAVTPDYSNNRLTL
	4.3%	CAWSQQGRAEQFF, CGARDVGLGVPEQFF	CAAEEESNNRIFF, CVLGLNNAGAKLTF
	2.7%	CASSLAGGSQNTLYF	CAVSAKNNAGAKLTF
	4.8%	CASSQEGDGERLFF	CAVSMTNTNKVVF, CAVSTENSGGSNAKLTF
	1.9%	CASSPGTGGYEQYF	CASNTGYQNFYF
Ipilimumab	1.6%	CASSQDIGWGVYAEQFF	CALVADSNYQLIW
	1.3%	CASSLELGGPEQYF	CAASDYSNNRLTL
	0.6%	CASSPQGAGTGQLYF	CARGSNNRIFF, CAVSAG DYAQGLTF
	5.7%	CASSQEGDGNTLYF	CALGENAPRF
	4.3%	CASSLGDRDTQYF, CASSPGTGGGTGQLYF	CAGTNYNQGKLIF
D11	4.2%	CASSLAWGGGANERLFF, CASSLEGGRKEQYF	CATDTDYSNNRLTL
	3.0%	CASSLVGGRDTQYF	CAVSMGNTNKVVF
	3.0%	CASGEPENTLYF	CAMREASSGSWQLIF
	15.3%	CASRGTGGAWAEQFF	CALVDLTTASLGKLQF
	8.8%	CASSPTGGGDTQYF	CAVTKDYSNNRLTL
D11+anti-PD-1	6.4%	CASSAPGGTETLYF	CALSDYSNNRLTL
	3.4%	CASSSGANSDYTF	CAMRELASSSFSKLVF
	2.3%	CASSLQANQDTQYF	CAARANYGNEKITF

The TCR frequencies are summarized and TCR β and TCR α sequences are shown.

*anti-PD-1* anti-programmed cell death protein-1, *CDR3* complementarity-determining region 3, *PBS* phosphate-buffered saline, *TCR* T-cell receptor.

**Table 3.** Summary of the validation assays for compounds targeting CTLA-4.

Variable	D11	A9	B10	D7	A6	Target Molecules
AlphaLISA (μM)	4.1	6	5.2	8.9	3.1	Human CTLA-4/CD80 (B7-1)
DSF	Y	Y	Y	Y	Y	Abatacept alone
SPR (μM)	22.5	20.8	106.5	240	No binding	Abatacept alone
INF-γ stimulation *ex vivo* (MC38)	Y	Y	Y	Y	Y	Mouse CTLA-4/CD80
T_conv_: T_reg_ decrease (human cells)	Y	Y	NA	NA	NA	Human CTLA-4/CD80
MC38 models	Y	Y	NA	NA	NA	Mouse or human CTLA-4/mouse CD80
RO5 violations	0	0	0	0	0	
RO3 violations	2	4	5	3	5	

The 5 top compounds were tested with in vitro and in vivo assays. The Lipinski rule of 5 was also summarized.

*AlphaLISA* Amplified Luminescent Proximity Homogeneous LISA, *CTLA-4* cytotoxic T-lymphocyte–associated protein 4, *DSF* differential scanning fluorimetry, *IFN-*γ interferon γ, *NA* not applicable, *RO3* rule of 3, *RO5* Lipinski Rule of 5, *SPR* surface plasmon resonance, *Y* yes.

## Data Availability

Single cell-sequencing data is readily available from GEO. To review GEO accession GSE228560, go to https://www.ncbi.nlm.nih.gov/geo/query/acc.cgi?acc=GSE228560 and enter the token cpataggavvirxub into the box.
