# Supplementary material for: Artificial intelligence-powered discovery of small molecules inhibiting CTLA-4 in cancer
Source: BJC Rep. Author manuscript; Available in PMC 2024 Feb 4. (PMC10838660; doi:10.1038/s44276-023-00035-5)
Supplement: Raw Data [file NIHMS1961340-supplement-Raw_Data.zip › RAWData/Figure 1/Figure 1i/B10 and A6 Creative Biolabs-CBLX073021-2A-SPR-Project report.pdf]

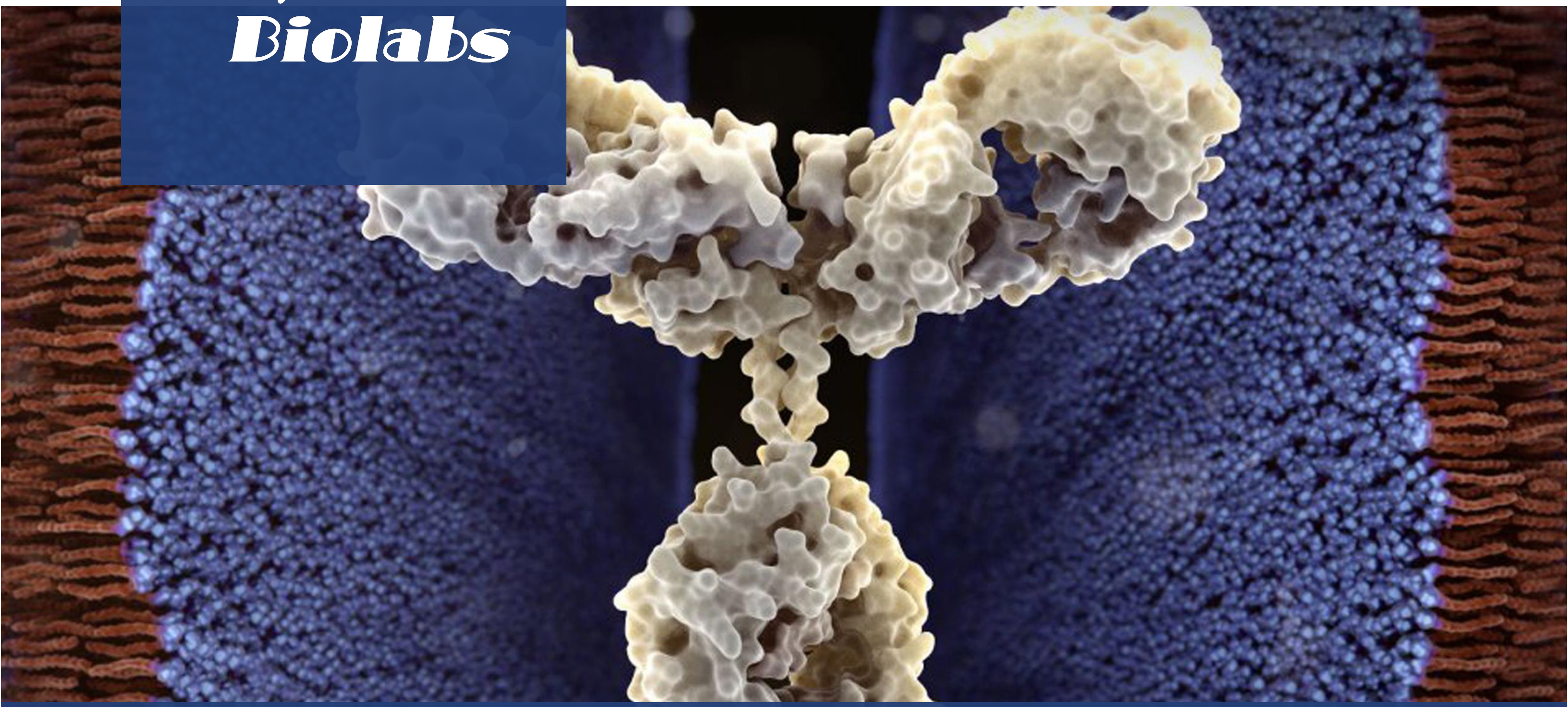

Project Report

Project ID: CBLX073021-2A-SPR

Date: Oct. 6, 2021

Xi Song, Ph.D.

Creative Biolabs Inc

Tel: 1-631-871-5806, 1-866-588-6325

Email: xsong@creative-biolabs.com

45-1 Ramsey Road, Shirley, NY 11967, USA

Contents

Cover Letter........1
Summary........2
Materials........3
Sample information........3
Equipment........3
Buffer information........3
Figures........4
Figure 1. Determination of the level of CTLA-4 immobilization onto the CM5 sensor chip........4
Figure 2. Binding and fitting curves between A6 with CTLA-4........5
Figure 3. Binding and fitting curves between B10 with CTLA-4........6
Table 1. Result Summary........7
Protocol........8
Terms and Conditions........9

Cover Letter

Dear Navid,

We have finished the binding affinity detection of A6, and B10 to CTLA-4 protein, respectively, using our surface plasmon resonance (SPR) platform.

Attached please find the detailed report.

Best regards,

Xi

Xi Song, Ph.D.
Creative Biolabs
45-1 Ramsey Road
Shirley, NY 11967, USA
Tel: 1-631-871-5806, 1-866-588-6325
Fax: 1-631-207-8356
Email: xsong@creative-biolabs.com
Web: www.creative-biolabs.com

Summary

The client provided us two molecules (analytes) and one CTLA-4 protein (ligand). We were contracted to measure the binding affinity of two molecules to CTLA-4 protein, respectively, by our SPR platform.

The CTLA-4 protein was immobilized onto the CM5 sensor chip surface with an immobilization level of about 10,000 RU. Then, the analyte (A6 or B10) at the concentration of 100, 50, 25, 12.5, 6.25, 3.125, 1.563, 0.781, and 0 μM, was injected into the sensor surface for interaction with the CTLA-4 protein, respectively. The steady-state affinity model or 1:1 binding model was used to measure the binding affinity and/or kinetics.

A6 binding to CTLA-4 protein

The fitting curves for A6 binding to CTLA-4 were shown in Figure 2. The A6 did not bind to the CTLA-4 protein.

B10 binding to CTLA-4 protein

The fitting curves for B10 binding to CTLA-4 were shown in Figure 3. Using the Steady-state affinity model, immobilized CTLA-4 on CM5 Chip can bind B10 with an affinity constant of 2.13×10^{-4} M.

Materials

Sample information

Name	Vendor	M.W.	Concentration	Buffer
A6	Client	392.471 Da	3.947 mg/mL	DMSO
B10	Client	341.292 Da	3.409 mg/mL	DMSO
CTLA-4	Client	43 kDa	1 mg/mL	PBS

Equipment

Name	Supplier	Cat. No.	Lot. No.
Biacore T200	Cytiva	N/A	N/A
Series S Sensor Chip CM5	Cytiva	BR100530	10305573
Amine Coupling Kit	Cytiva	BR100050	31765

Buffer information

Name	Detail
Immobilization Buffer	10 mM Sodium Acetate, pH 4.5
Immobilized Ligand	30 μg/mL CTLA-4 in Immobilization Buffer, 10 μL/min, immobilized about 15,000 RU
Running Buffer	1×PBS with 0.05%Tween-20, pH7.4, 5% DMSO
Association and dissociation flow rate	30 μL/min, Association 60 s, dissociation 90 s

Other regular reagents: purchased locally.

Figures

Figure 1. Determination of the level of CTLA-4 immobilization onto the CM5 sensor chip

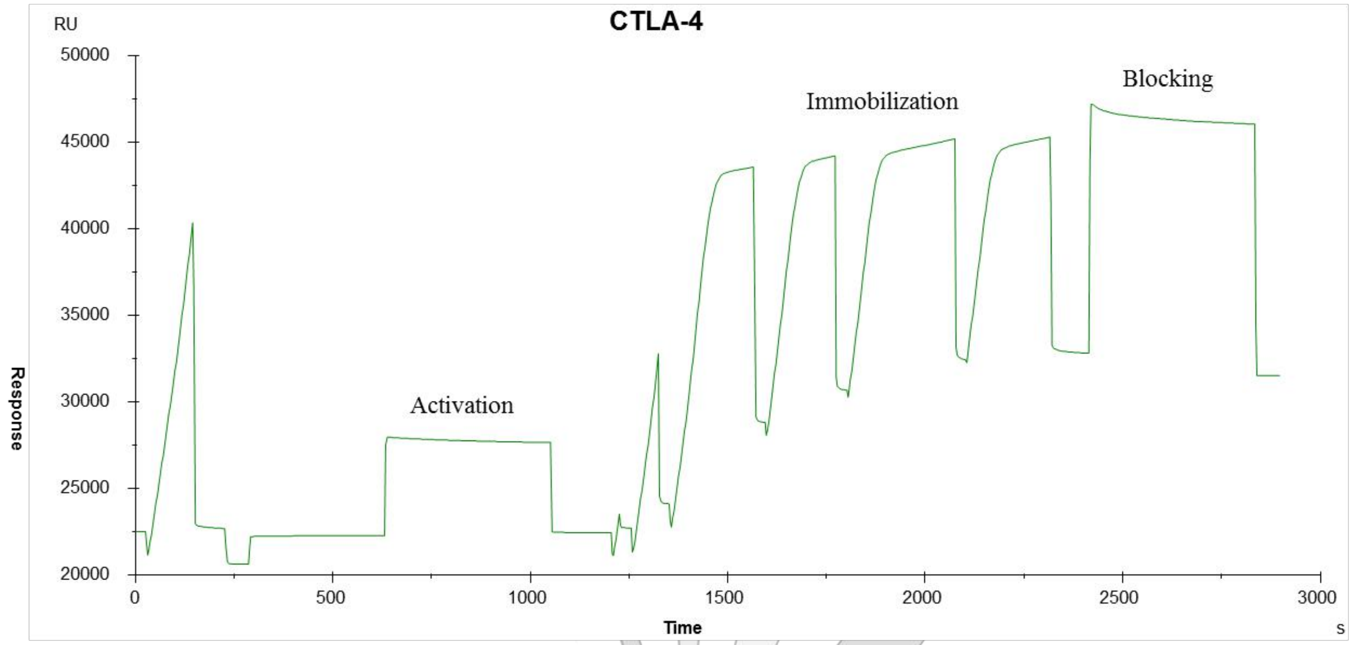

Figure 2. Binding and fitting curves between A6 with CTLA-4

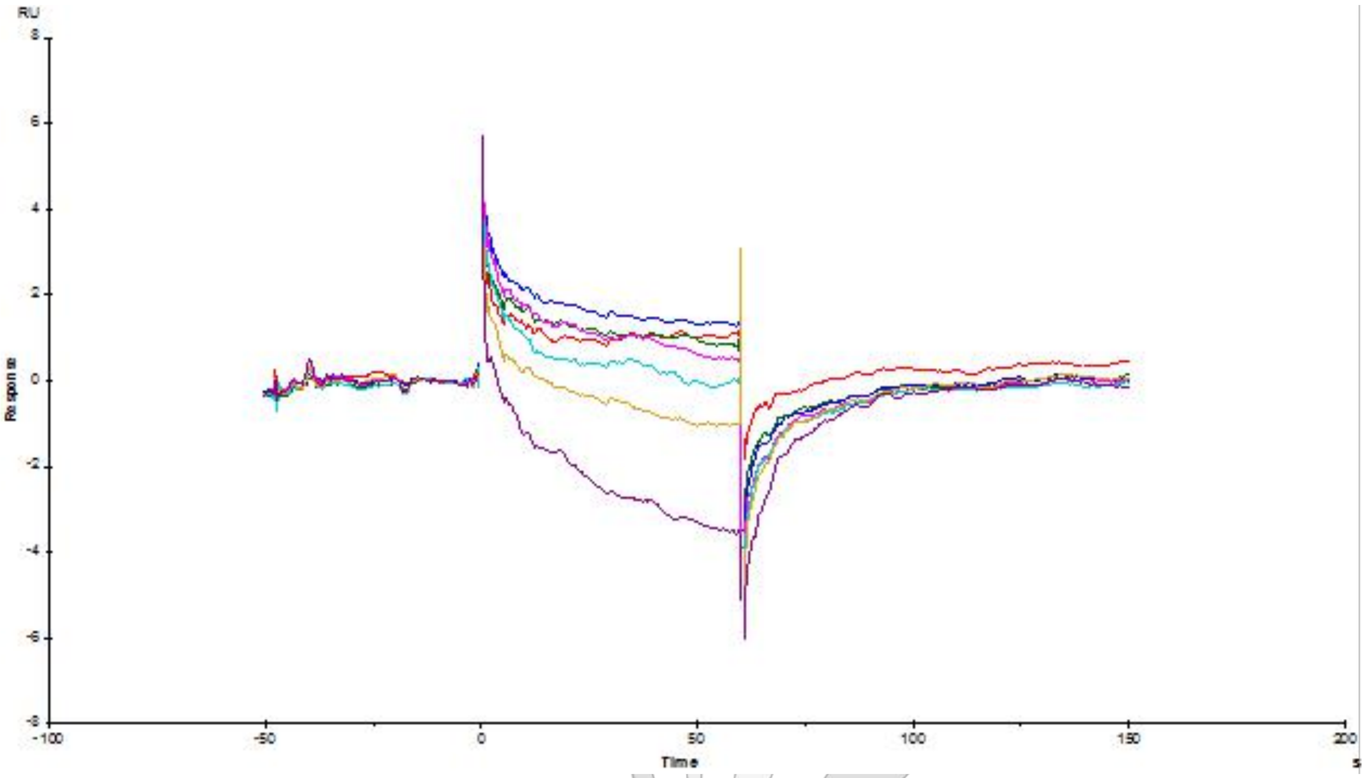

The A6 did not bind to the CTLA-4 protein.

Figure 3. Binding and fitting curves between B10 with CTLA-4

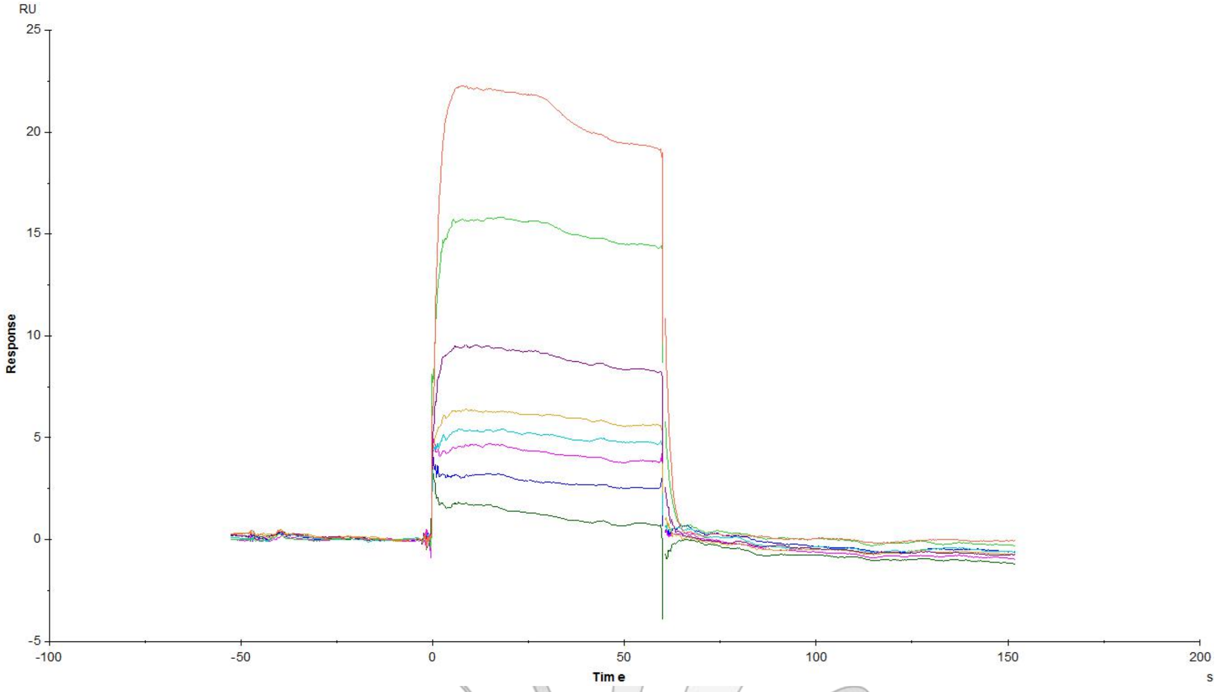

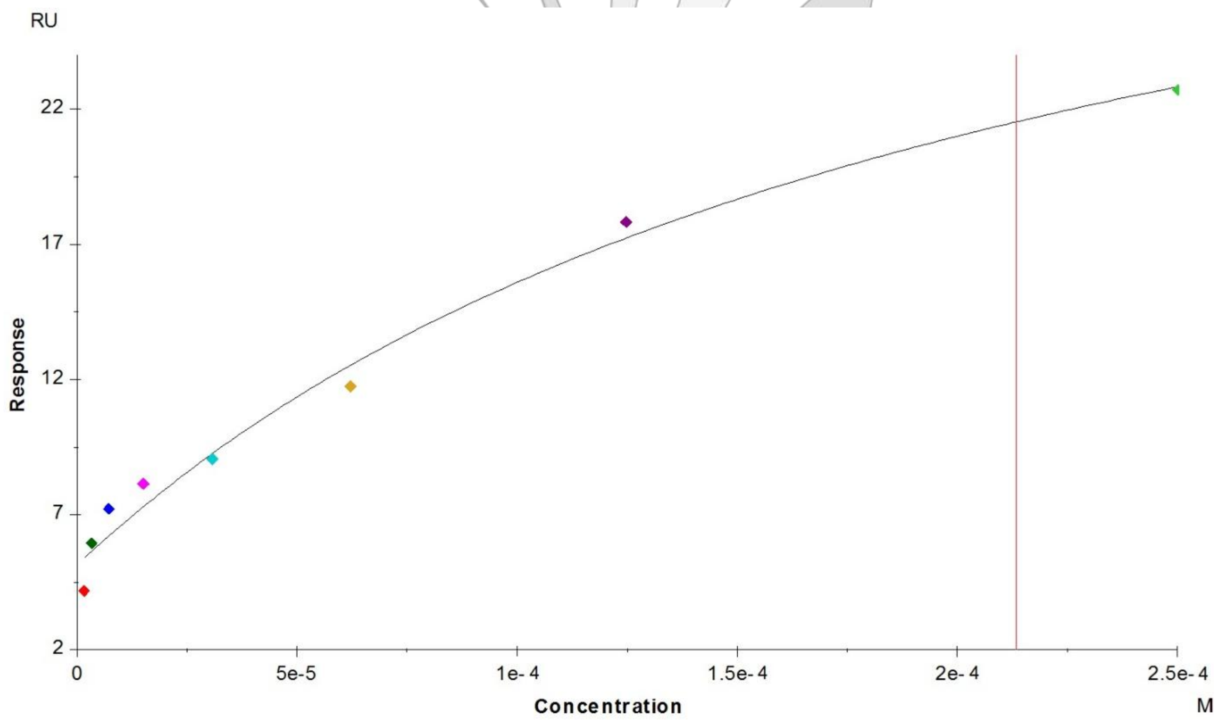

Steady State Affinity

Table 1. Result Summary

Method	Ligand	Immobilized Level (RU)	Analyte	Analyte Conc.	KD (M)	Rmax (RU)	Chi^2 (RU^2)	Fit method
CM5	CTLA-4	10354.7	A6	100-0.781 μM	No binding			
CM5	CTLA-4	10354.7	B10	100-0.781 μM	2.13E-04	32.82	0.87	Steady State Affinity

45-1 Ramsey Road, Shirley, NY 11967, USA
Tel: 1-631-381-2994
Email: info@creative-biolabs.com

Protocol

Ligand Immobilization

Affinity analysis was carried out using a Biacore T200 instrument. CTLA-4 protein was directly immobilized on the CM5 chip using an amine coupling kit. Before immobilization, the CM5 sensor surface was activated using a mixture of 400 mM 1-ethyl-3-(3-dimethylaminopropyl) carbodiimide (EDC) and 100 mM N-hydroxysuccinimide (NHS). Then, 30 μg/ml of CTLA-4 in immobilization buffer (10 mM NaAc (pH 4.5)) was then injected into Fc2 sample channel at a flow rate of 10 μl/min. The amount of ligand immobilized was about 10,000 RU. The chip was deactivated by 1 M Ethanolamine hydrochloride-NaOH at a flow rate of 10 μl/min for 420 s. The reference Fc1 channel underwent similar procedures but without injecting the ligand.

Interaction & Analysis

The analyte (A6 or B10) was serially diluted with the running buffer to give a concentration of 100, 50, 25, 12.5, 6.25, 3.125, 1.563, 0.781, and 0 μM, respectively. Different concentrations of analytes were then injected into the cell over both channels at a flow rate of 30 μl/min, with a contact time of 60 s, followed by a dissociation time of 90 s. Data analysis was performed on the Biacore T200 computer and with the Biacore T200 evaluation software, using the steady-state affinity model or 1:1 binding model.

Creative Biolabs®
45-1 Ramsey Road, Shirley, NY 11967, USA
Tel: 1-631-381-2994
Email: info@creative-biolabs.com

Terms and Conditions

FOR PROVISION OF SERVICES

1) **Project Lifecycle Management Process.** For each project, one senior project manager will talk with Client first. Based on the full discussion, this manager will provide a quote with tailored protocol. After that, we may revise the quote according to the Client's new specifications. A quote from Creative Biolabs Inc [CBL hereafter] and accepted by the Client will serve as a services agreement. After that, the project is passed on to an experienced project manager and his team. Bi-weekly reports or monthly reports are generated by the project manager and sent to the Client. Usually the protocol indicated in the approved quote is strictly followed; in case there is an issue, our project manager will talk with the Client and try to have a resolution. Sometime, an interim report is sent to a Client. Upon completion of a project, a final report with technical details, e.g. DNA Sequencing data, Protein SDS-Page Image… is generated by the assigned project manager. The final report together with any material products are sent to the Client.
2) **Standard of Performance.** All Services will be performed using due care in accordance with (a) the Services agreement [the quote] and (b) generally prevailing industry standards applicable to such Services. A good faith effort to start and complete all Services on time will be made, and Client will be notified if CBL determines that there are likely to be substantial delays. CBL expressly guarantees to perform all its procedures with professional diligence, and strives to perform quality work acceptable to all its Clients. Should quote proposal and study scope not align with Client's expectations, it is the Client's responsibility to notify CBL *in writing* of any changes to this proposal prior to initiation of the project. CBL is only responsible for performing services as outlined in this quote, unless other agreed upon in writing by CBL and Client.
3) **Risk Share**. CBL indicates the risk [also the non-refundable fees] in the approved quote clearly. Due to the business nature of custom services, CBL pays greater attention on continuous communication with Clients. CBL writes down everything in the quote, including protocol, options and costs at each step of a project. The next step is almost always determined by the achievement of the first step. CBL communicates with Clients in a timely manner to decide which option should be taken as the next step. It is at CBL's discretion when a project should be stopped due to a failure and what non-refundable fees should be charged.
4) **Limited Warranty.** The Services Agreement is a contract for services only. CBL's sole warranty with respect to the Services is that CBL will perform all Services in accordance with the standard of performance set forth above. Client shall notify CBL in writing of any claim for a breach of such warranty by CBL within one month after delivery by CBL of the last-to-deliver Deliverable relating to such Services. The sole remedy of Client for breach of such warranty shall be to require CBL to re-perform the Services (or such portion thereof as may reasonably be

required to be re-performed),and, in such event CBL shall diligently pursue the re-performance of the Services or portions thereof until completion, or, if CBL cannot re-perform the Services (or such portion) in accordance with this limited warranty, then it shall refund amounts paid by the Client for the applicable Service giving rise to the breach of warranty. CBL makes no expressed or implied warranties for the results that are obtained and unless there is an instrument malfunction, a technician error, or some fault directly attributable to CBL. CBL will not be held liable for results obtained with Client's samples.

5) **Payments.** Usually a non-refundable upfront payment is required to commence a project. Invoices will be sent to Client before initiation of each Phase of a project. Invoice terms are net 30 days. The invoiced fee should be paid at the initiation of each Phase. CBL shall invoice for all services and/or products provided which may increase or decrease the total estimated costs as outlined in this quote. Additional services required to complete this project will incur additional charges. Occasionally, invoices may be sent to Client upon completion of the Services and delivery to Client of the applicable Deliverable(s). If Client defaults in any payment when due, CBL, at its option and without prejudice to its other lawful remedies, may delay performance, defer delivery, charge interest on undisputed amounts owed, and/or terminate the Services Agreement [the quote]. All payments due hereunder shall be made in the currency specified by CBL in writing. Pricing and turnaround times are estimates based on information provided by Client and are subject to change. Payment in full is required from oversea projects unless previous arrangements are made with CBL.

6) **Intellectual Property.** Except as set forth otherwise in the quote, Client shall be the exclusive owner of, and CBL hereby assigns to Client, all Client Materials, Data, inventions, improvements, designs, programs, formulas, know-how and writings supplied by Client and/or generated or discovered as a direct result of CBL's performance of the Services, whether or not copyrightable or patentable (collectively, the "Inventions"). CBL waives all the rights to the intellectual property involved in this project.

7) **Confidentiality.** During performance of the Services and for 3 years thereafter, CBL will treat all information related to this project as proprietary and confidential and will not disclose the same to any person except its employees, consultants, and subcontractors to whom it is necessary to disclose the information for purposes of providing the Services; CBL may disclose the information to its employees, consultants, or subcontractors, provided that such employees, consultants, or subcontractors are subject to a written agreement that includes confidentiality terms at least as restrictive as those specified herein. Any employee, consultant, or subcontractor who is given access to the information shall be informed by CBL of these terms and conditions.

Contact Us

45-1 Ramsey Road Shirley, NY 11967, USA

Tel: 1-631-381-2994

Fax: 1-631-207-8356

Email: info@creative-biolabs.com

http://www.creative-biolabs.com

Creative Biolabs®
