# Supplementary material for: Artificial intelligence-powered discovery of small molecules inhibiting CTLA-4 in cancer
Source: BJC Rep. Author manuscript; Available in PMC 2024 Feb 4. (PMC10838660; doi:10.1038/s44276-023-00035-5)
Supplement: Raw Data [file NIHMS1961340-supplement-Raw_Data.zip › RAWData/Figure 2/2b-d/Figure 2b-d.pptx]

## Slide 1
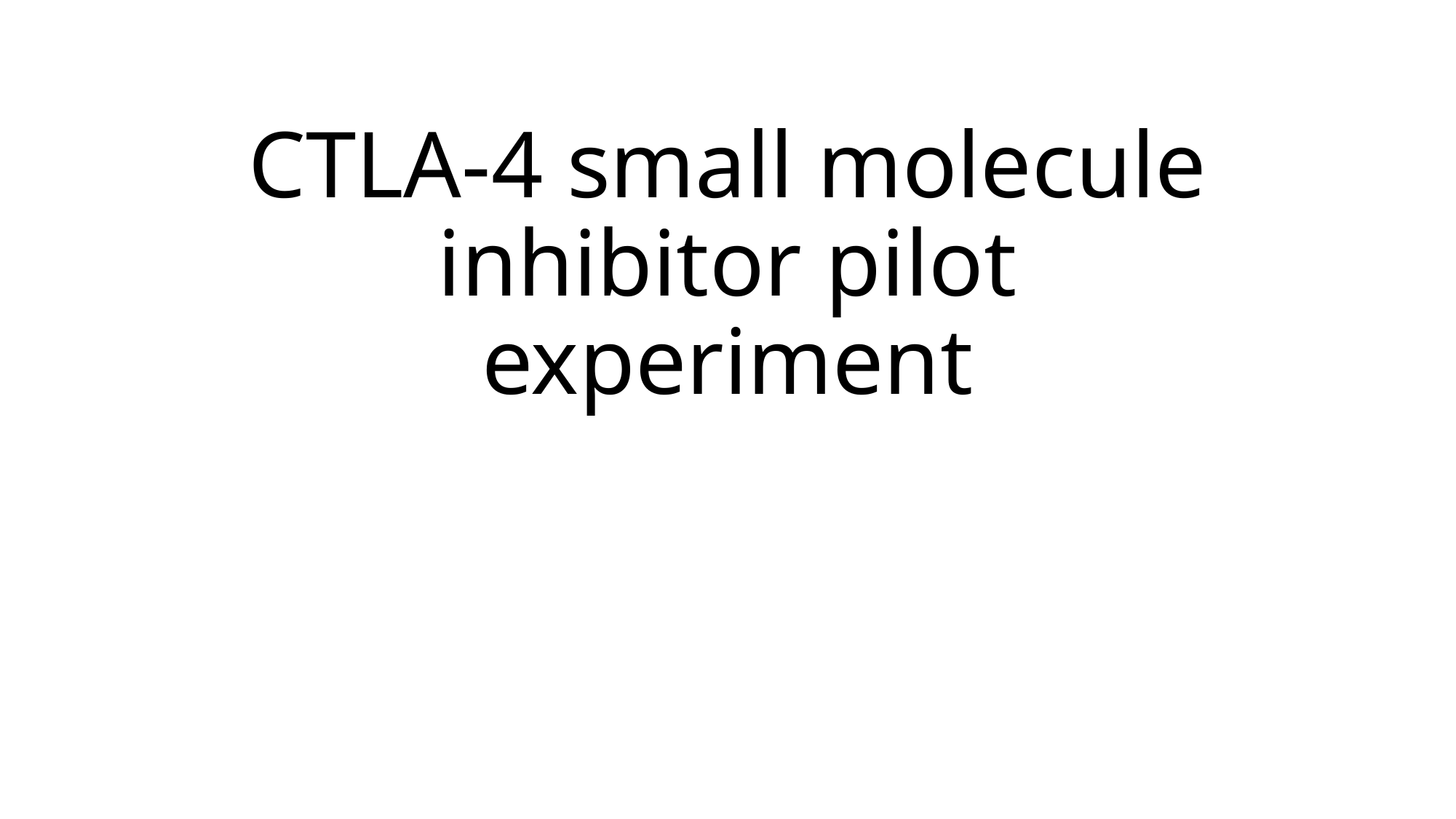

# CTLA-4 small molecule inhibitor pilot experiment

## Slide 2
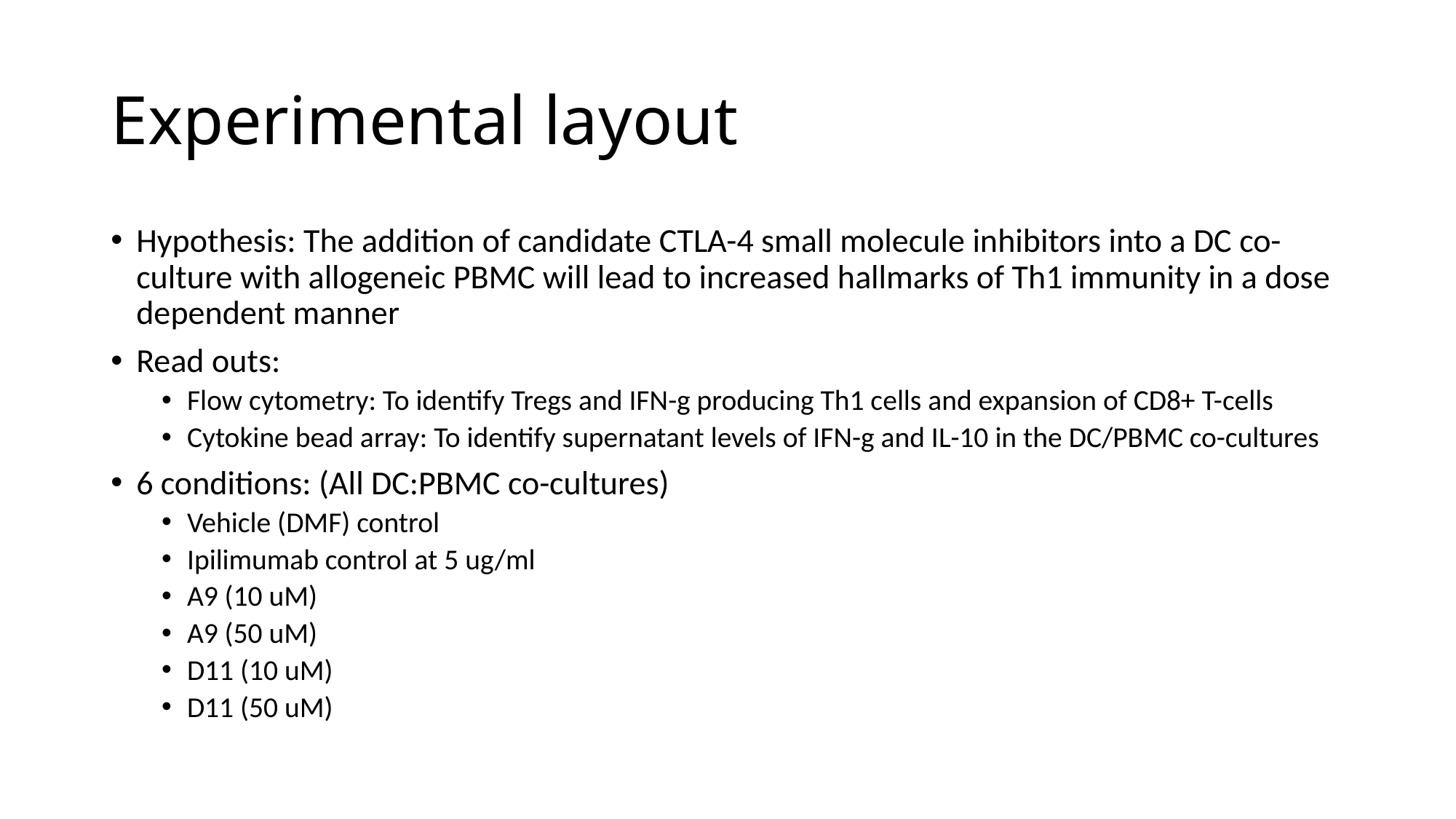

# Experimental layout
Hypothesis: The addition of candidate CTLA-4 small molecule inhibitors into a DC co-culture with allogeneic PBMC will lead to increased hallmarks of Th1 immunity in a dose dependent manner
Read outs:
Flow cytometry: To identify Tregs and IFN-g producing Th1 cells and expansion of CD8+ T-cells
Cytokine bead array: To identify supernatant levels of IFN-g and IL-10 in the DC/PBMC co-cultures
6 conditions: (All DC:PBMC co-cultures)
Vehicle (DMF) control
Ipilimumab control at 5 ug/ml
A9 (10 uM)
A9 (50 uM)
D11 (10 uM)
D11 (50 uM)

## Slide 3
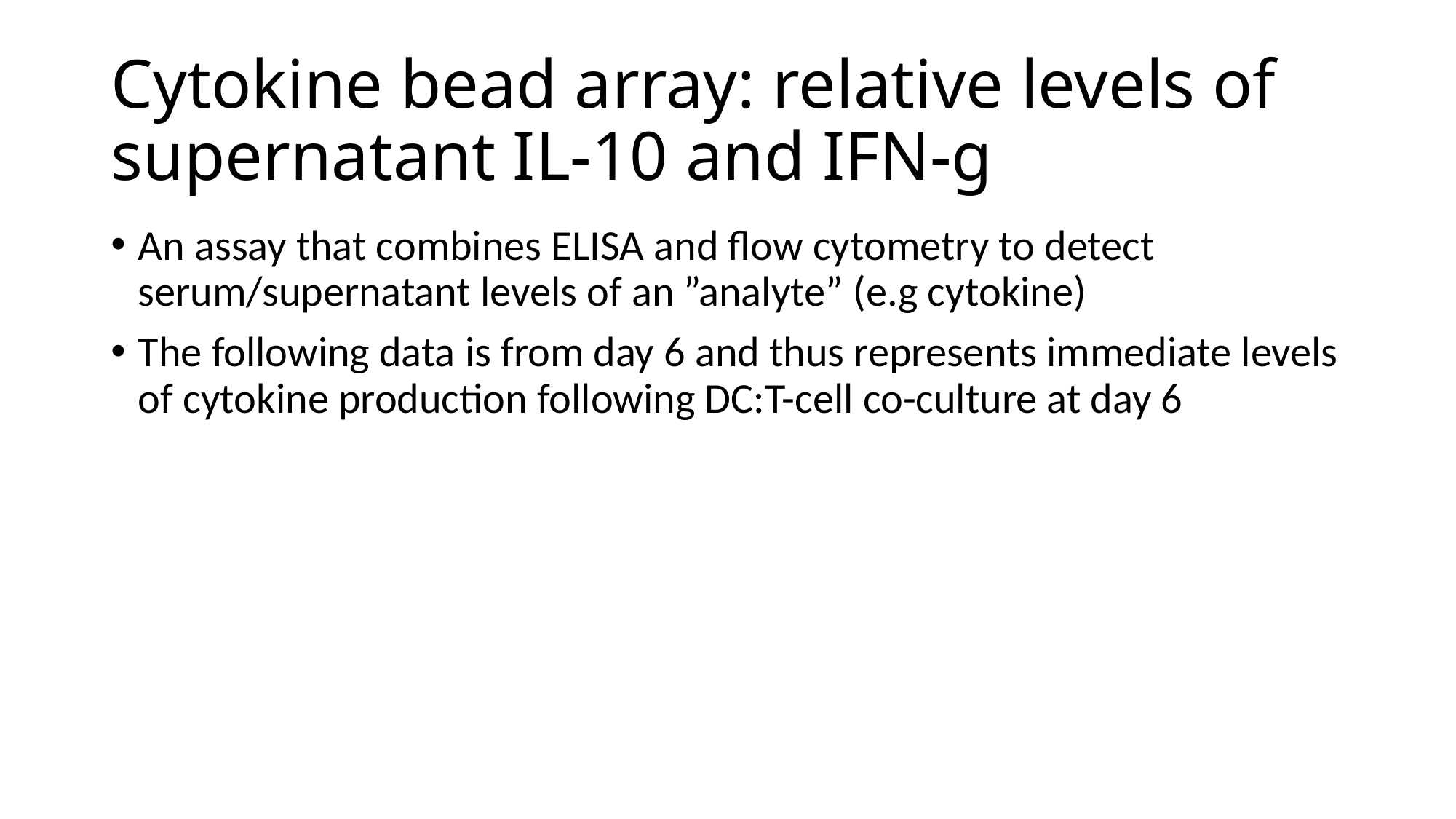

# Cytokine bead array: relative levels of supernatant IL-10 and IFN-g
An assay that combines ELISA and flow cytometry to detect serum/supernatant levels of an ”analyte” (e.g cytokine)
The following data is from day 6 and thus represents immediate levels of cytokine production following DC:T-cell co-culture at day 6

## Slide 4
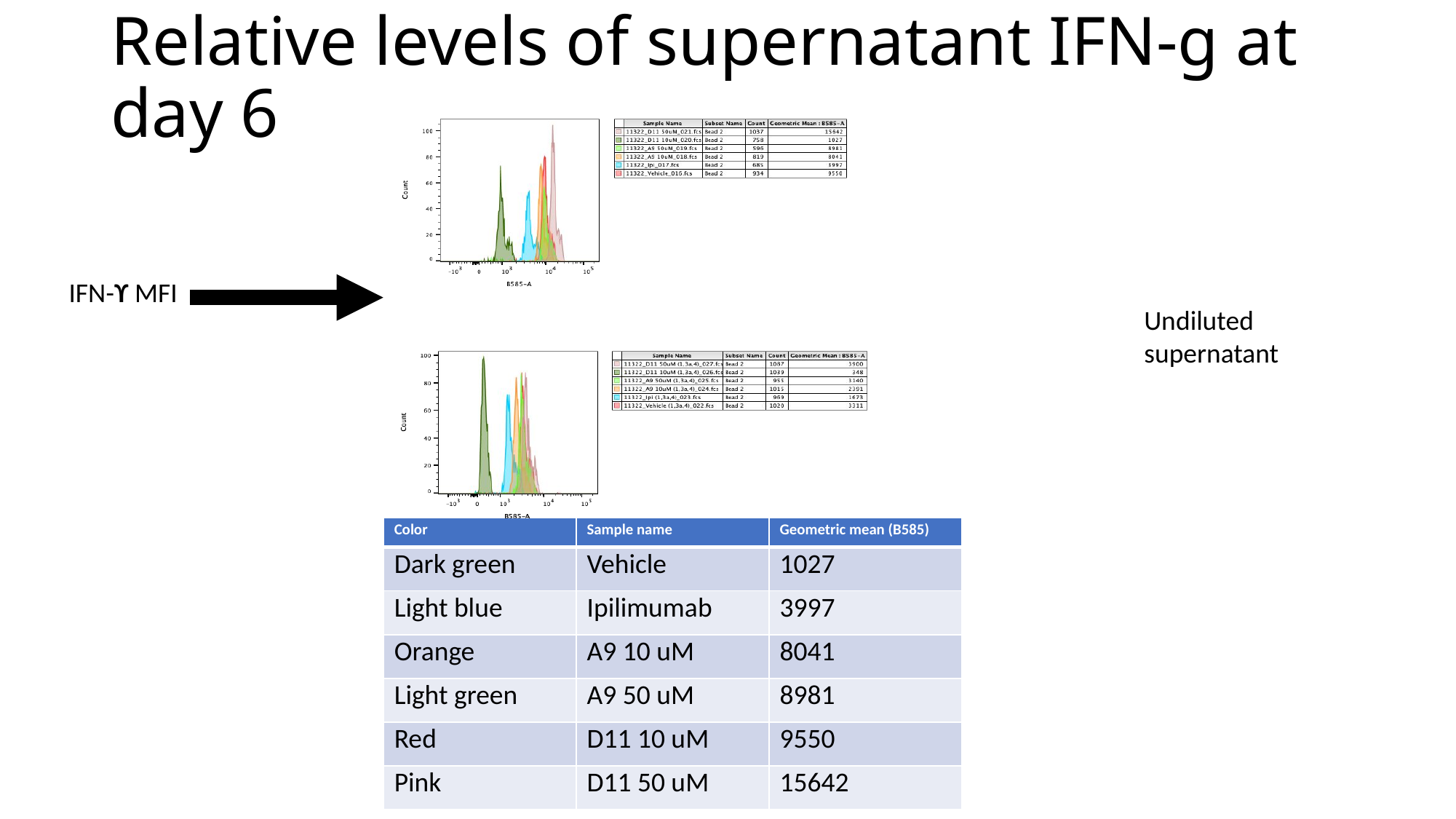

# Relative levels of supernatant IFN-g at day 6
IFN-ϒ MFI
Undiluted supernatant
| Color | Sample name | Geometric mean (B585) |
| --- | --- | --- |
| Dark green | Vehicle | 1027 |
| Light blue | Ipilimumab | 3997 |
| Orange | A9 10 uM | 8041 |
| Light green | A9 50 uM | 8981 |
| Red | D11 10 uM | 9550 |
| Pink | D11 50 uM | 15642 |

## Slide 5
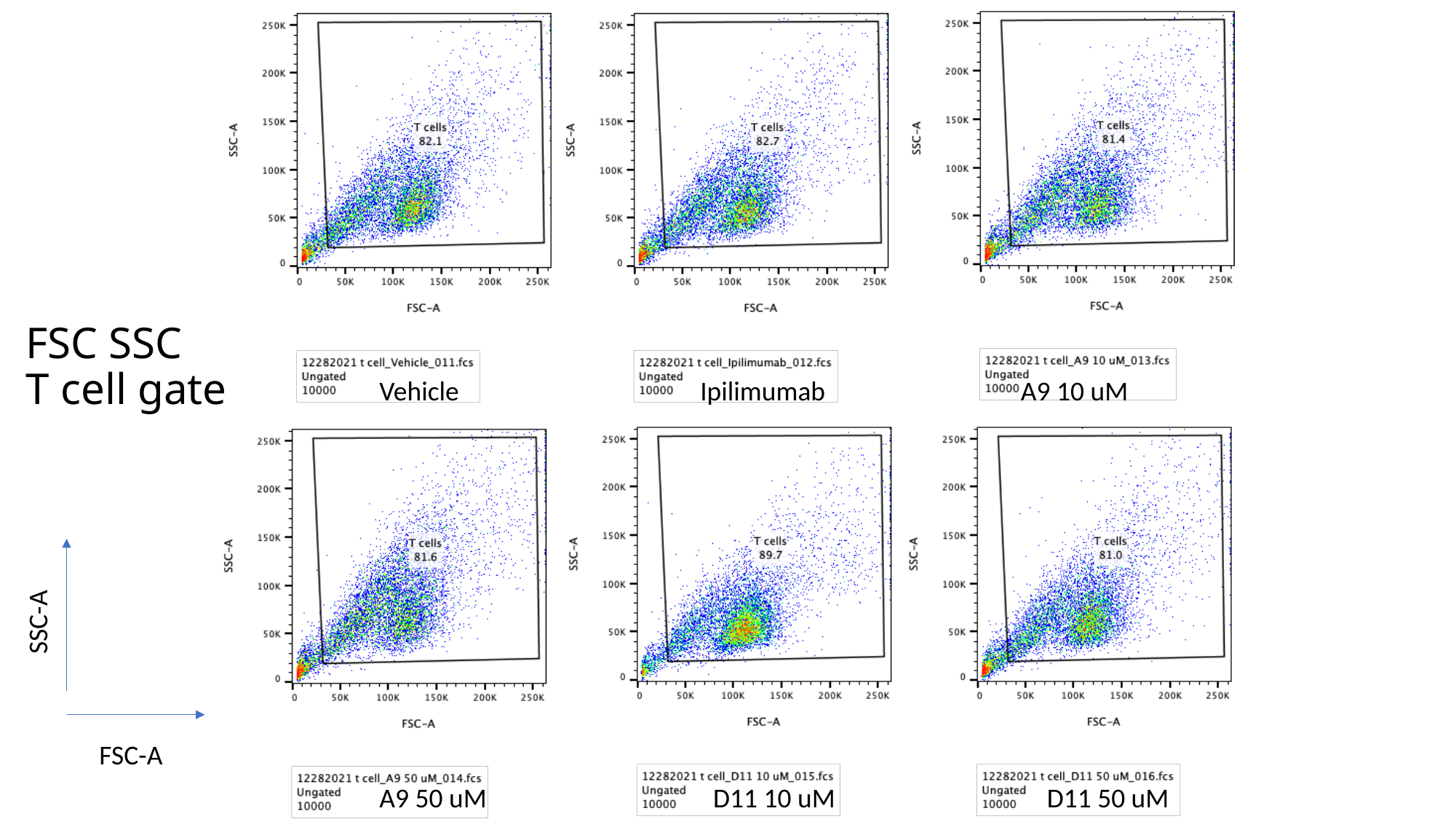

# FSC SSC T cell gate
Vehicle
Ipilimumab
A9 10 uM
SSC-A
FSC-A
A9 50 uM
D11 10 uM
D11 50 uM

## Slide 6
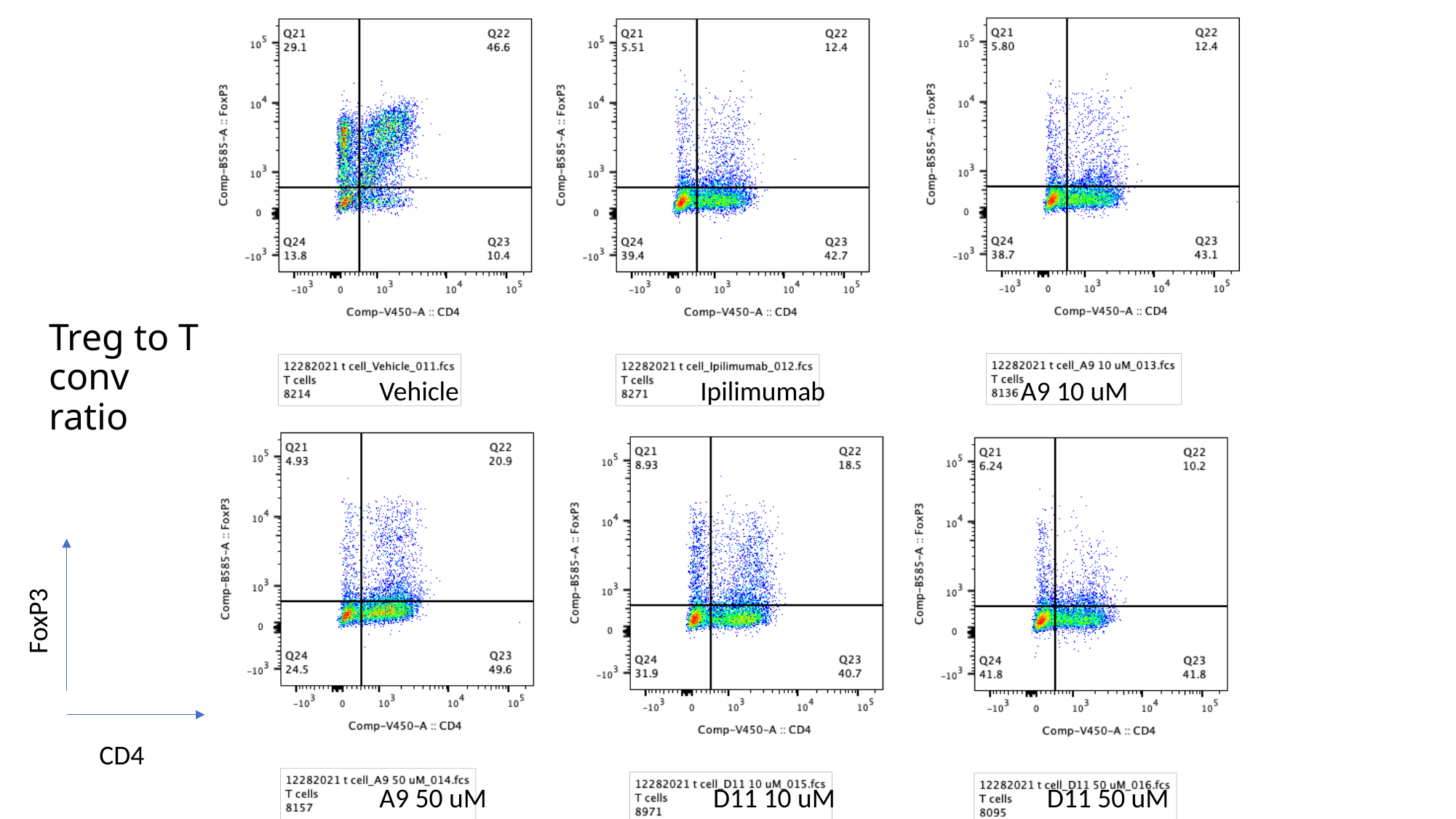

# Treg to T conv ratio
Vehicle
Ipilimumab
A9 10 uM
FoxP3
CD4
A9 50 uM
D11 10 uM
D11 50 uM

## Slide 7
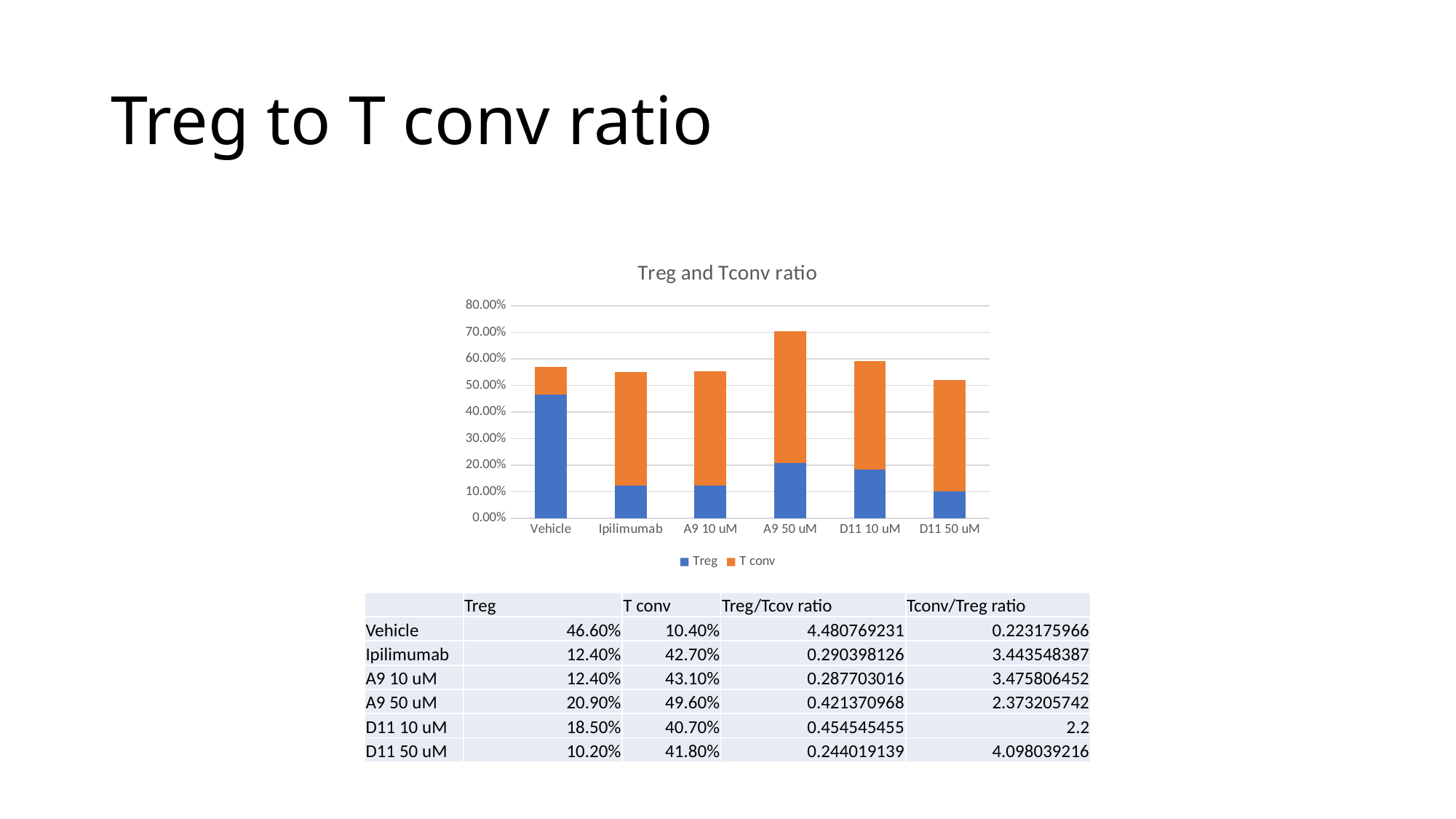

# Treg to T conv ratio
### Chart: Treg and Tconv ratio
| Category | Treg | T conv |
|---|---|---|
| Vehicle | 0.466 | 0.104 |
| Ipilimumab | 0.124 | 0.427 |
| A9 10 uM | 0.124 | 0.431 |
| A9 50 uM | 0.209 | 0.496 |
| D11 10 uM | 0.185 | 0.40700000000000003 |
| D11 50 uM | 0.102 | 0.418 || | Treg | T conv | Treg/Tcov ratio | Tconv/Treg ratio |
| --- | --- | --- | --- | --- |
| Vehicle | 46.60% | 10.40% | 4.480769231 | 0.223175966 |
| Ipilimumab | 12.40% | 42.70% | 0.290398126 | 3.443548387 |
| A9 10 uM | 12.40% | 43.10% | 0.287703016 | 3.475806452 |
| A9 50 uM | 20.90% | 49.60% | 0.421370968 | 2.373205742 |
| D11 10 uM | 18.50% | 40.70% | 0.454545455 | 2.2 |
| D11 50 uM | 10.20% | 41.80% | 0.244019139 | 4.098039216 |

## Slide 8
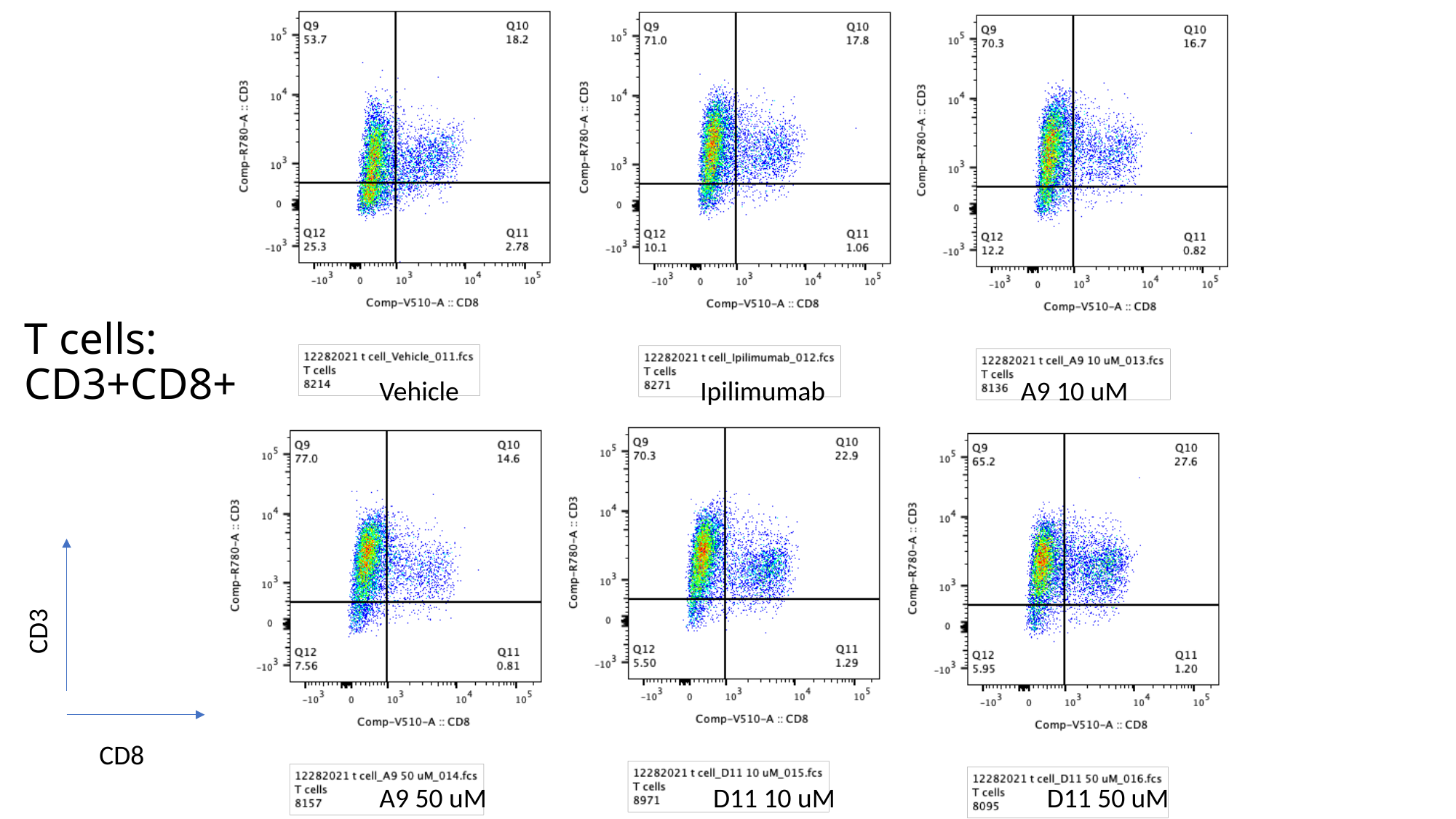

# T cells: CD3+CD8+
Vehicle
Ipilimumab
A9 10 uM
CD3
CD8
A9 50 uM
D11 10 uM
D11 50 uM

## Slide 9
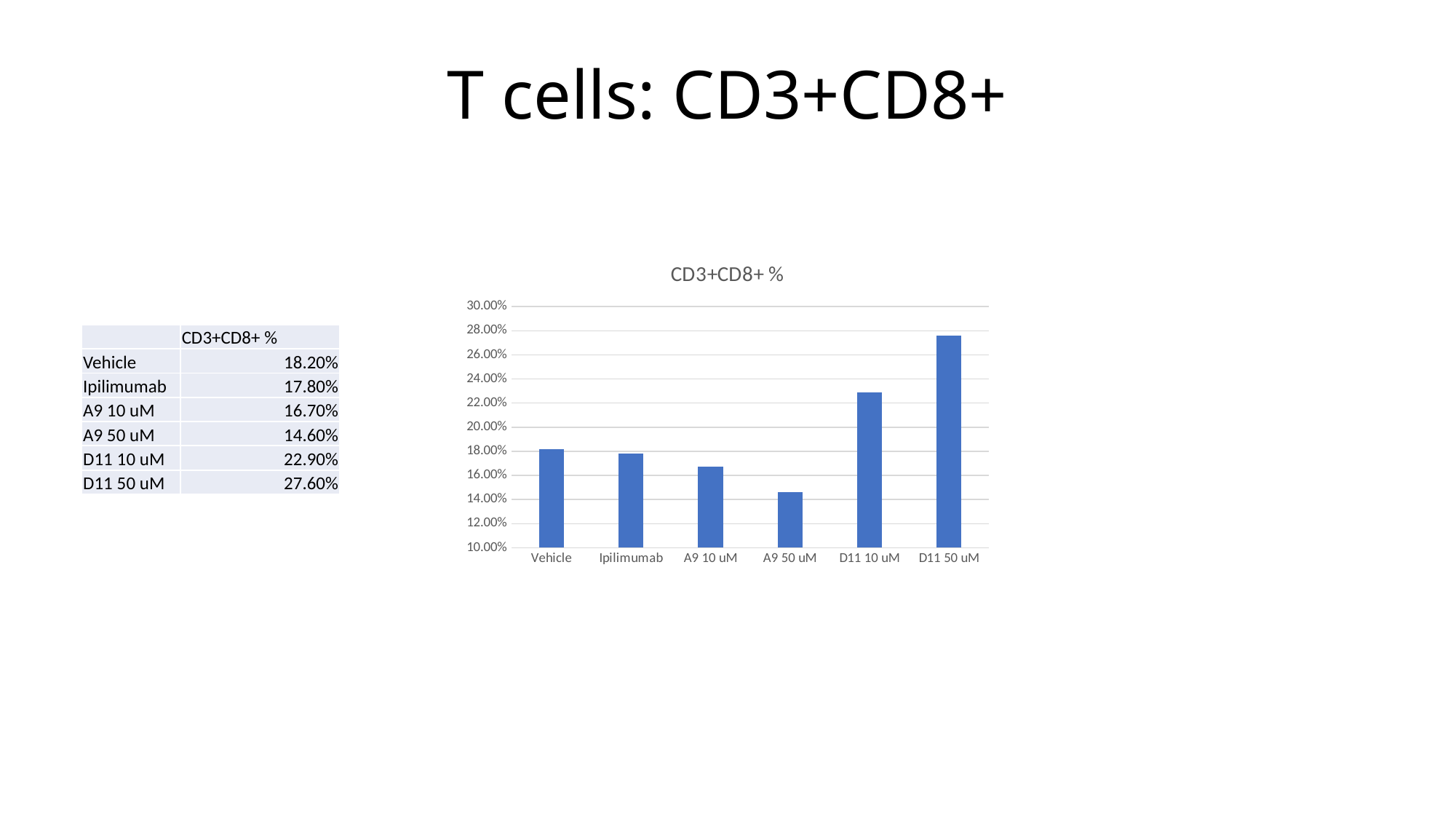

# T cells: CD3+CD8+
### Chart:
| Category | CD3+CD8+ % |
|---|---|
| Vehicle | 0.182 |
| Ipilimumab | 0.178 |
| A9 10 uM | 0.167 |
| A9 50 uM | 0.146 |
| D11 10 uM | 0.229 |
| D11 50 uM | 0.276 || | CD3+CD8+ % |
| --- | --- |
| Vehicle | 18.20% |
| Ipilimumab | 17.80% |
| A9 10 uM | 16.70% |
| A9 50 uM | 14.60% |
| D11 10 uM | 22.90% |
| D11 50 uM | 27.60% |

## Slide 10
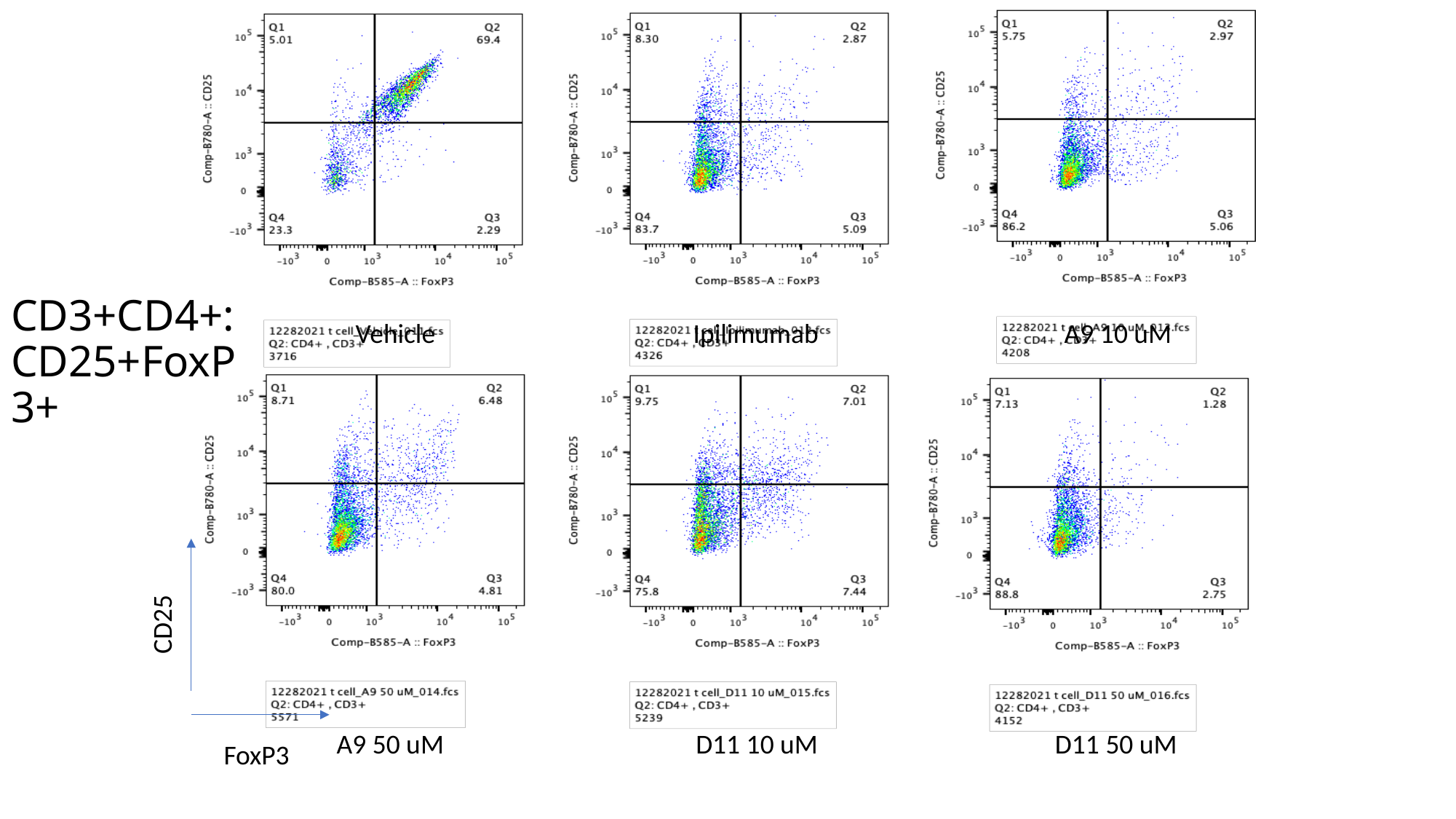

# CD3+CD4+: CD25+FoxP3+
Vehicle
Ipilimumab
A9 10 uM
CD25
A9 50 uM
D11 10 uM
D11 50 uM
FoxP3

## Slide 11
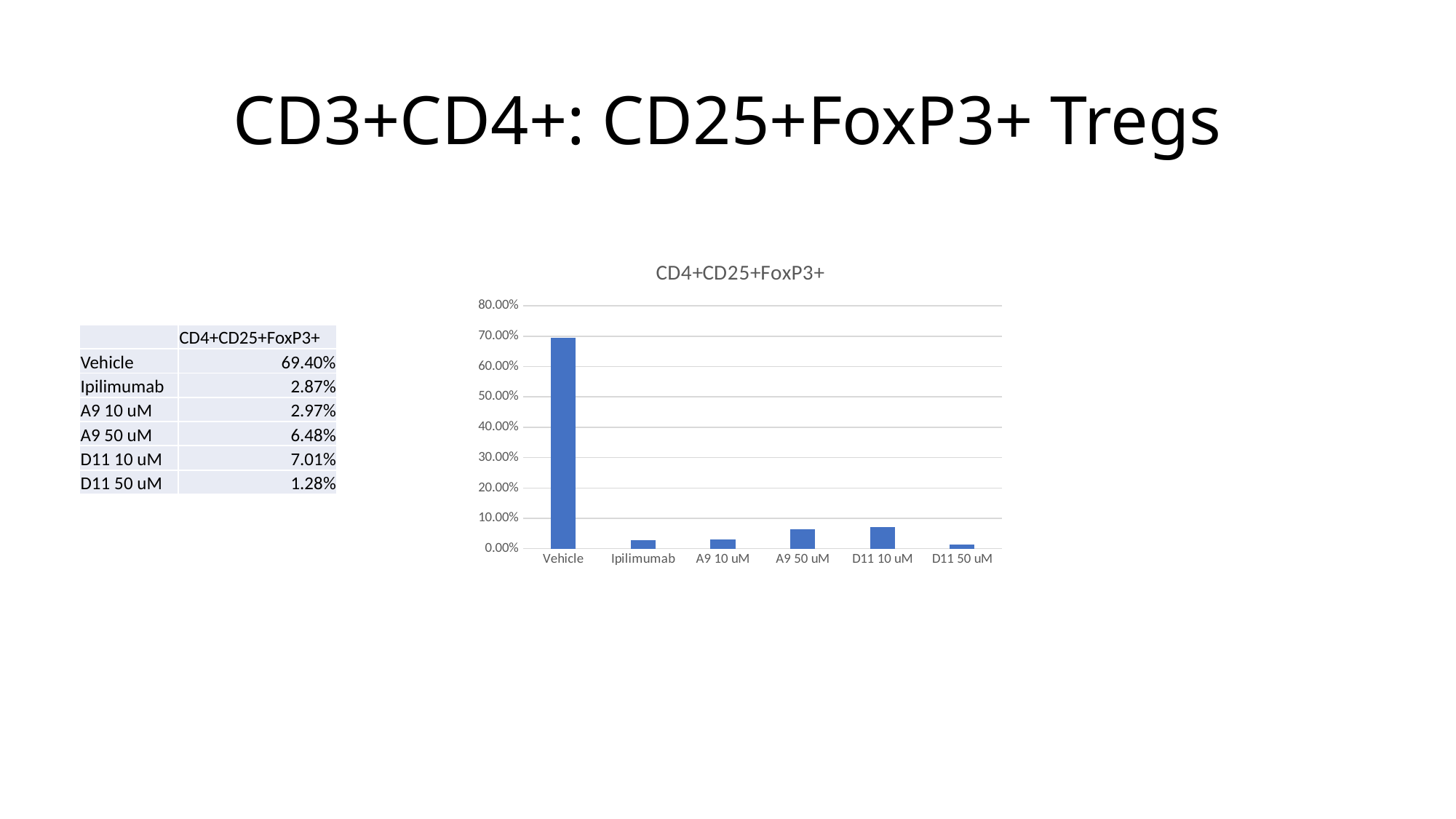

# CD3+CD4+: CD25+FoxP3+ Tregs
### Chart:
| Category | CD4+CD25+FoxP3+ |
|---|---|
| Vehicle | 0.694 |
| Ipilimumab | 0.0287 |
| A9 10 uM | 0.0297 |
| A9 50 uM | 0.0648 |
| D11 10 uM | 0.0701 |
| D11 50 uM | 0.0128 || | CD4+CD25+FoxP3+ |
| --- | --- |
| Vehicle | 69.40% |
| Ipilimumab | 2.87% |
| A9 10 uM | 2.97% |
| A9 50 uM | 6.48% |
| D11 10 uM | 7.01% |
| D11 50 uM | 1.28% |
