# Supplementary material for: Artificial intelligence-powered discovery of small molecules inhibiting CTLA-4 in cancer
Source: BJC Rep. Author manuscript; Available in PMC 2024 Feb 4. (PMC10838660; doi:10.1038/s44276-023-00035-5)
Supplement: Raw Data [file NIHMS1961340-supplement-Raw_Data.zip › RAWData/Figure 5/Figure 5c/Figure 5c.pptx]

## Slide 1
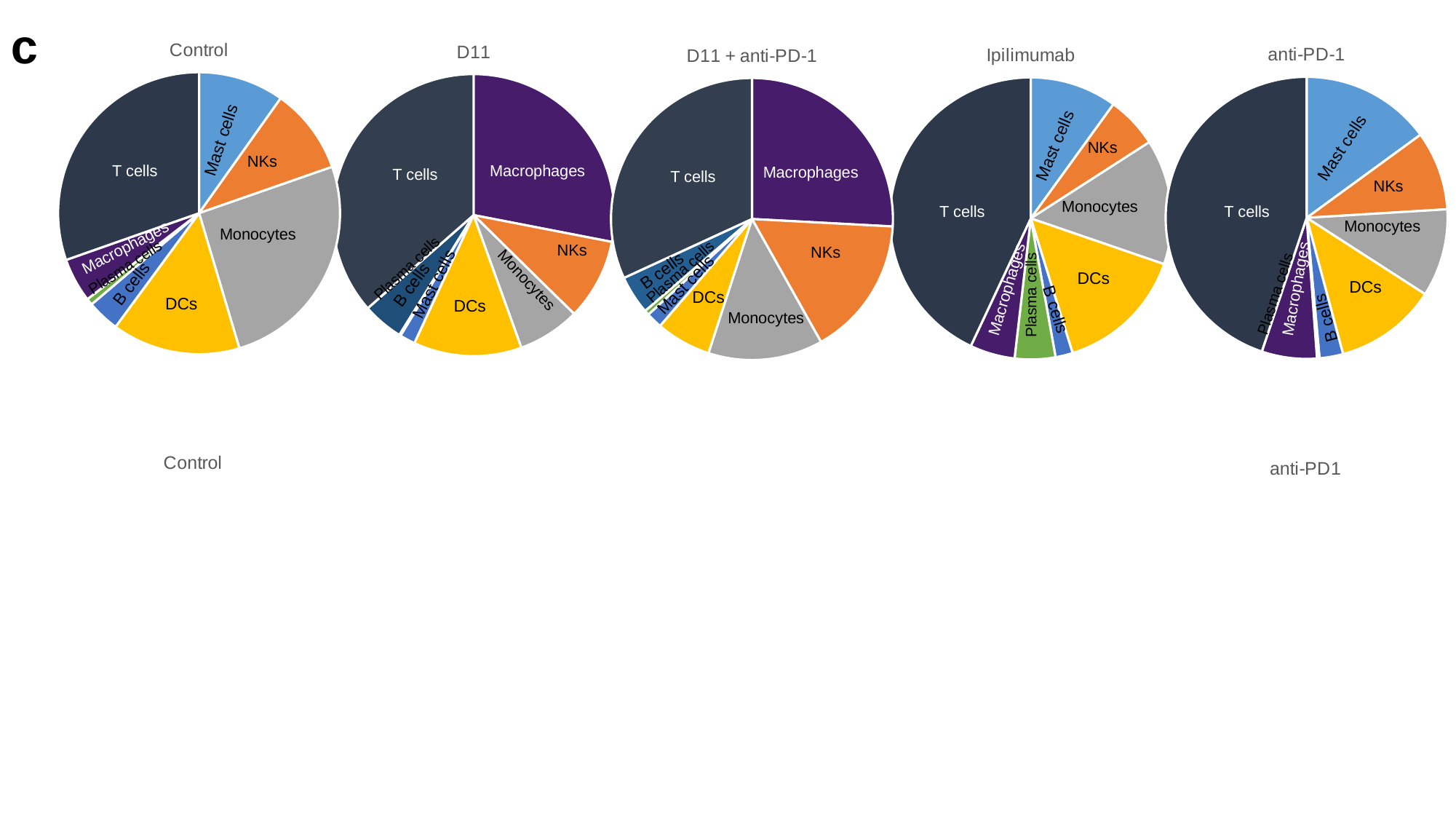

c
### Chart: Control
| Category | |
|---|---|
| Macrophages | 7.898172323759792 |
| NKs | 7.898172323759792 |
| Monocytes | 20.64295039164491 |
| DCs | 11.81462140992167 |
| mast cells | 2.9862924281984333 |
| plasma cells | 0.60378590078329 |
| B cells | 4.06331592689295 |
| T cells | 24.39621409921671 |
### Chart: D11
| Category | |
|---|---|
| Macrophages | 21.416735708367852 |
| NKs | 7.104391052195526 |
| Monocytes | 5.447390223695112 |
| DCs | 9.475973487986744 |
| mast cells | 1.356669428334714 |
| plasma cells | 0.12427506213753108 |
| B cells | 3.572908036454018 |
| T cells | 27.858326429163213 |
### Chart: anti-PD-1
| Category | |
|---|---|
| Macrophages | 10.849862078024431 |
| NKs | 6.567713122290818 |
| Monocytes | 7.3032969919873905 |
| DCs | 8.49862078024432 |
| mast cells | 1.9571785104426638 |
| plasma cells | 0.21016681991330616 |
| B cells | 4.584263759358991 |
| T cells | 32.51017995533955 |
### Chart: Ipilimumab
| Category | |
|---|---|
| Macrophages | 6.477516059957174 |
| NKs | 3.8410064239828694 |
| Monocytes | 9.314775160599572 |
| DCs | 9.68950749464668 |
| mast cells | 1.284796573875803 |
| plasma cells | 3.024625267665953 |
| B cells | 3.345824411134904 |
| T cells | 27.904175588865098 |
### Chart: D11 + anti-PD-1
| Category | |
|---|---|
| Macrophages | 20.65772290157901 |
| NKs | 12.786418140804939 |
| Monocytes | 10.506945268906566 |
| DCs | 5.045708179983379 |
| mast cells | 1.472159563101033 |
| plasma cells | 0.4392734180220824 |
| B cells | 3.549804107800071 |
| T cells | 25.465985990739647 |Mast cells
Mast cells
NKs
Mast cells
NKs
T cells
Macrophages
Macrophages
T cells
T cells
NKs
Monocytes
T cells
T cells
Monocytes
Monocytes
Macrophages
NKs
NKs
Plasma cells
Plasma cells
B cells
Plasma cells
DCs
Monocytes
B cells
Mast cells
Mast cells
B cells
DCs
Macrophages
Macrophages
Plasma cells
Plasma cells
DCs
DCs
DCs
B cells
Monocytes
B cells
### Chart: Control
| Category |
|---|
### Chart: anti-PD1
| Category |
|---|
