# Supplementary material for: Artificial intelligence-powered discovery of small molecules inhibiting CTLA-4 in cancer
Source: BJC Rep. Author manuscript; Available in PMC 2024 Feb 4. (PMC10838660; doi:10.1038/s44276-023-00035-5)
Supplement: Raw Data [file NIHMS1961340-supplement-Raw_Data.zip › RAWData/Figure 5/Figure 5d/Figure 5d.pptx]

## Slide 1
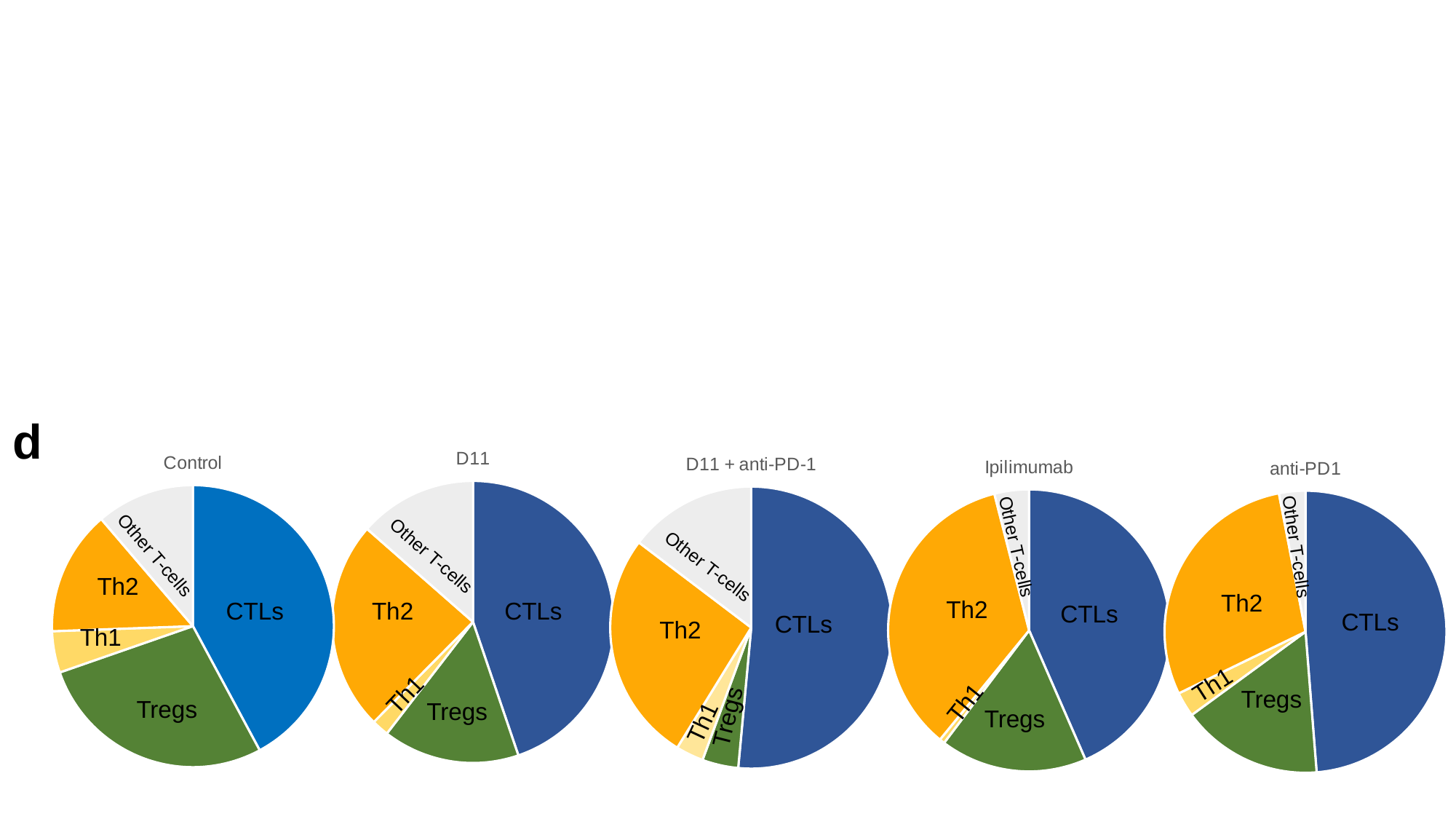

d
### Chart: D11
| Category | |
|---|---|
| CTLs | 14.42626346313173 |
| Tregs | 5.0539 |
| Th1 | 0.6317316 |
| Th2 | 7.746 |
| Other T-cells | 4.359983429991715 |
### Chart: Control
| Category | |
|---|---|
| CTLs | 11.586161879895561 |
| Tregs | 7.5718 |
| Th1 | 1.305483 |
| Th2 | 3.933 |
| Other T-cells | 3.100522193211488 |
### Chart: D11 + anti-PD-1
| Category | |
|---|---|
| CTLs | 15.362697376231745 |
| Tregs | 1.2347 |
| Th1 | 0.9497804 |
| Th2 | 7.919 |
| Other T-cells | 4.380861925679687 |
### Chart: Ipilimumab
| Category | |
|---|---|
| CTLs | 12.620449678800858 |
| Tregs | 4.8983 |
| Th1 | 0.1739829 |
| Th2 | 10.21 |
| Other T-cells | 1.1509635974304069 |
### Chart: anti-PD1
| Category | |
|---|---|
| CTLs | 18.20570077499015 |
| Tregs | 6.0554 |
| Th1 | 1.0639695 |
| Th2 | 10.9 |
| Other T-cells | 1.1296466570340207 |Other T-cells
Other T-cells
Other T-cells
Other T-cells
Other T-cells
Th2
Th2
Th2
CTLs
CTLs
Th2
CTLs
CTLs
CTLs
Th2
Th1
Th1
Th1
Tregs
Th1
Tregs
Tregs
Tregs
Tregs
Th1
