# Supplementary figures and images for: Artificial intelligence-powered discovery of small molecules inhibiting CTLA-4 in cancer
Source: BJC Rep. Author manuscript; Available in PMC 2024 Feb 4. (PMC10838660; doi:10.1038/s44276-023-00035-5)

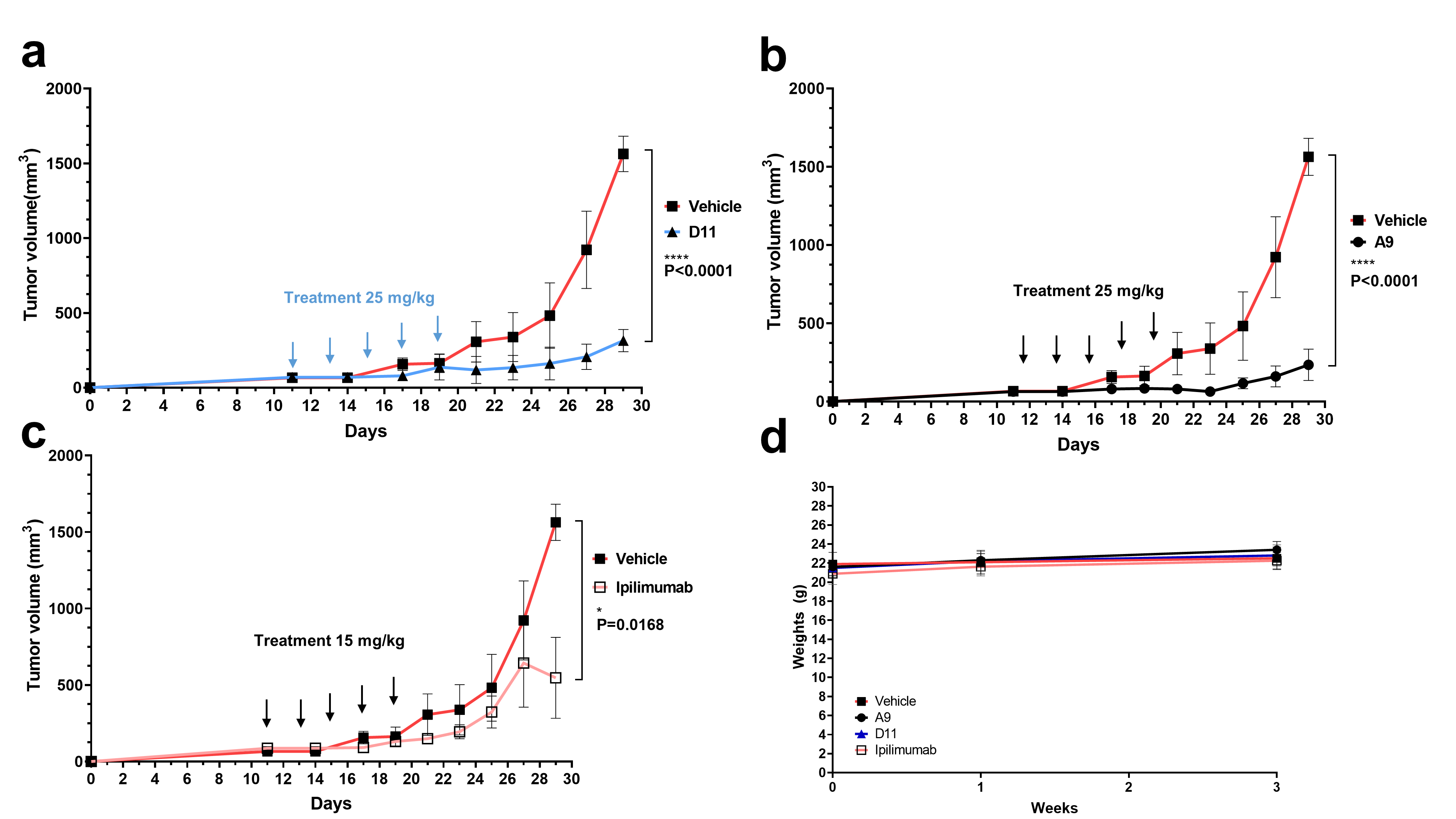

Supplement: Raw Data [file NIHMS1961340-supplement-Raw_Data.zip › RAWData/Figure 4/Figure 4.TIF]

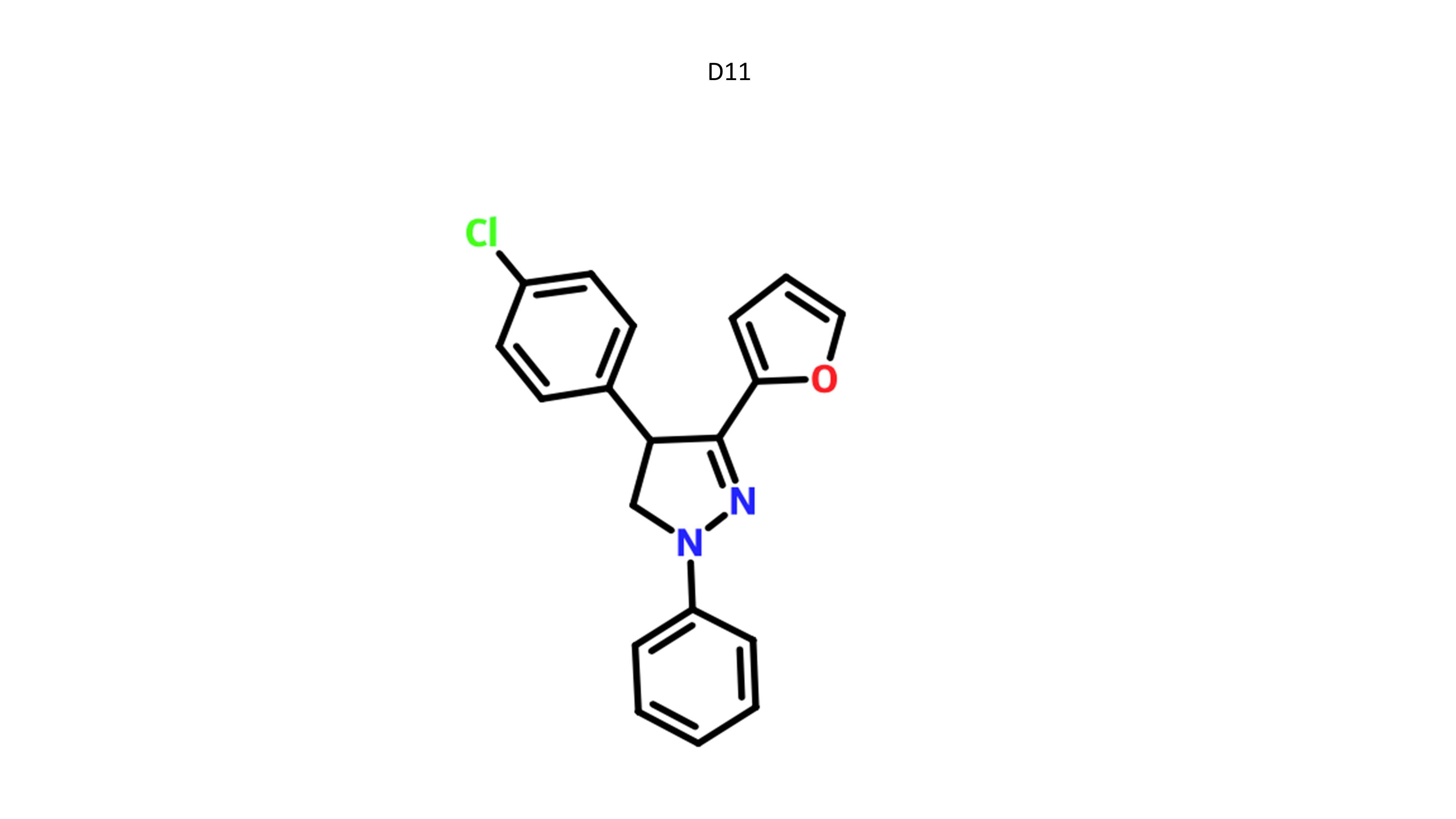

Supplement: Raw Data [file NIHMS1961340-supplement-Raw_Data.zip › RAWData/Figure 1/Figure 1f/Figure 1f chemical structure.TIF]

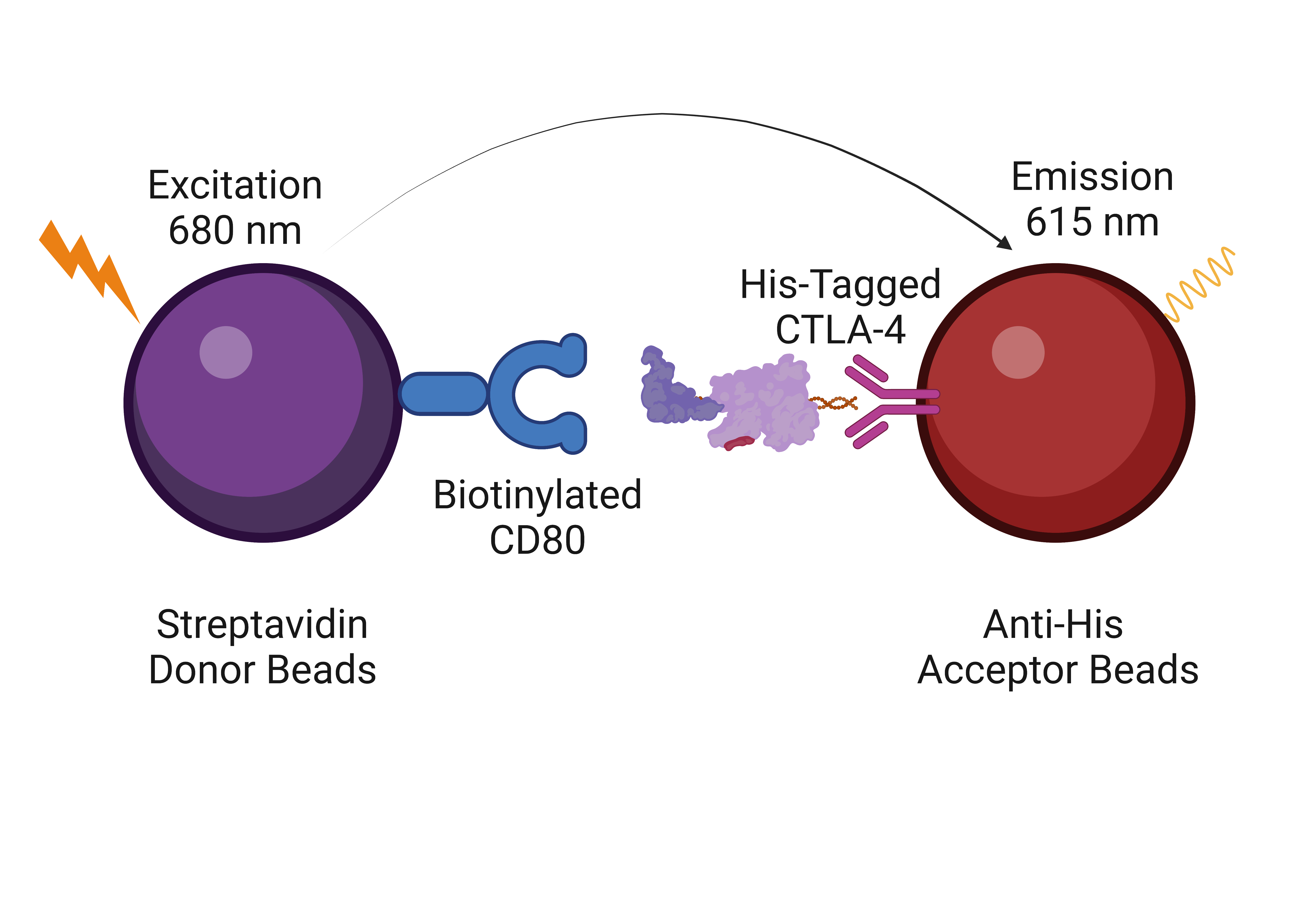

Supplement: Raw Data [file NIHMS1961340-supplement-Raw_Data.zip › RAWData/Figure 1/Figure 1a/Figure 1a.png]

## Slide 1
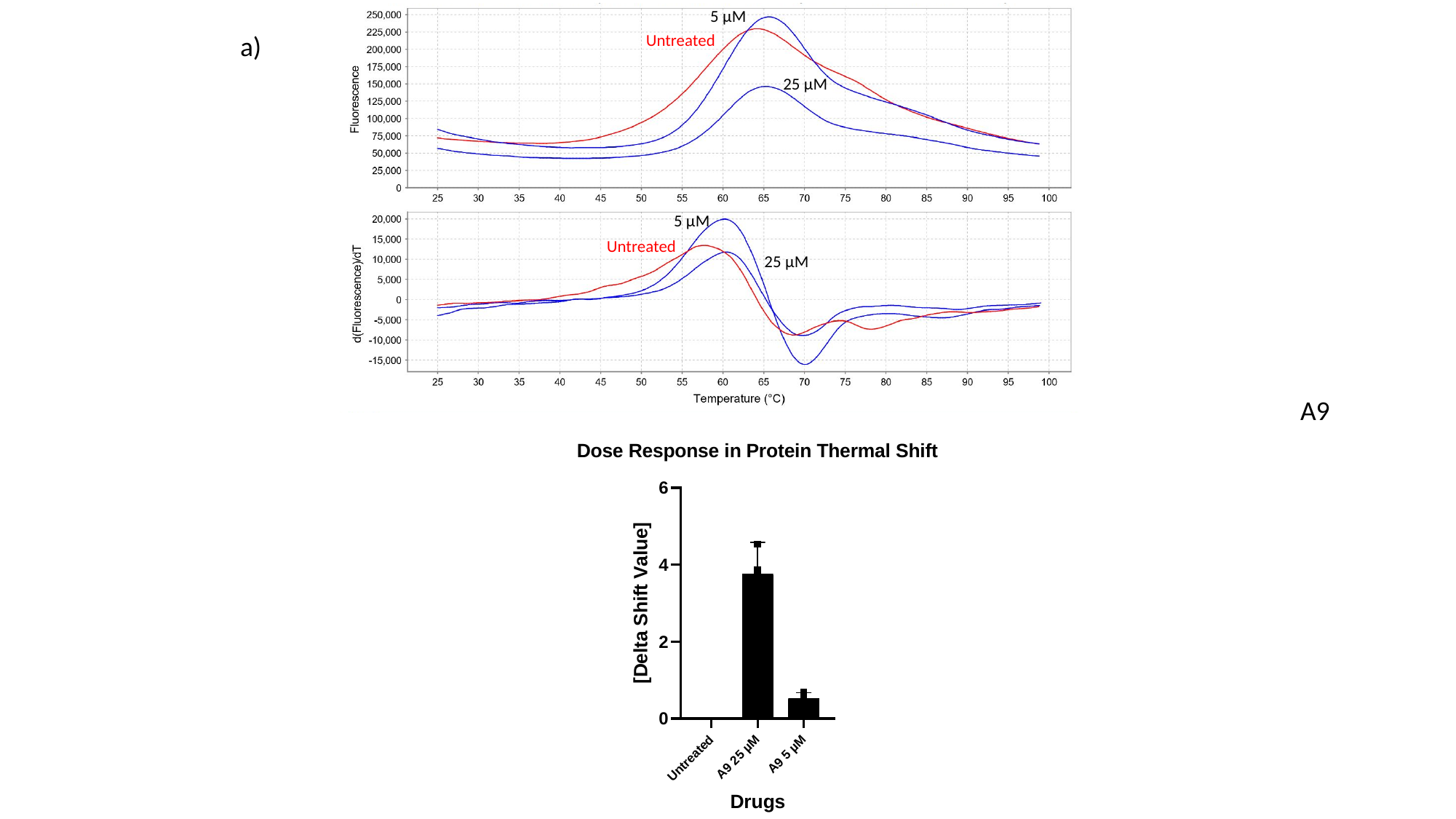

5 µM
a)
Untreated
25 µM
5 µM
Untreated
25 µM
A9

## Slide 2
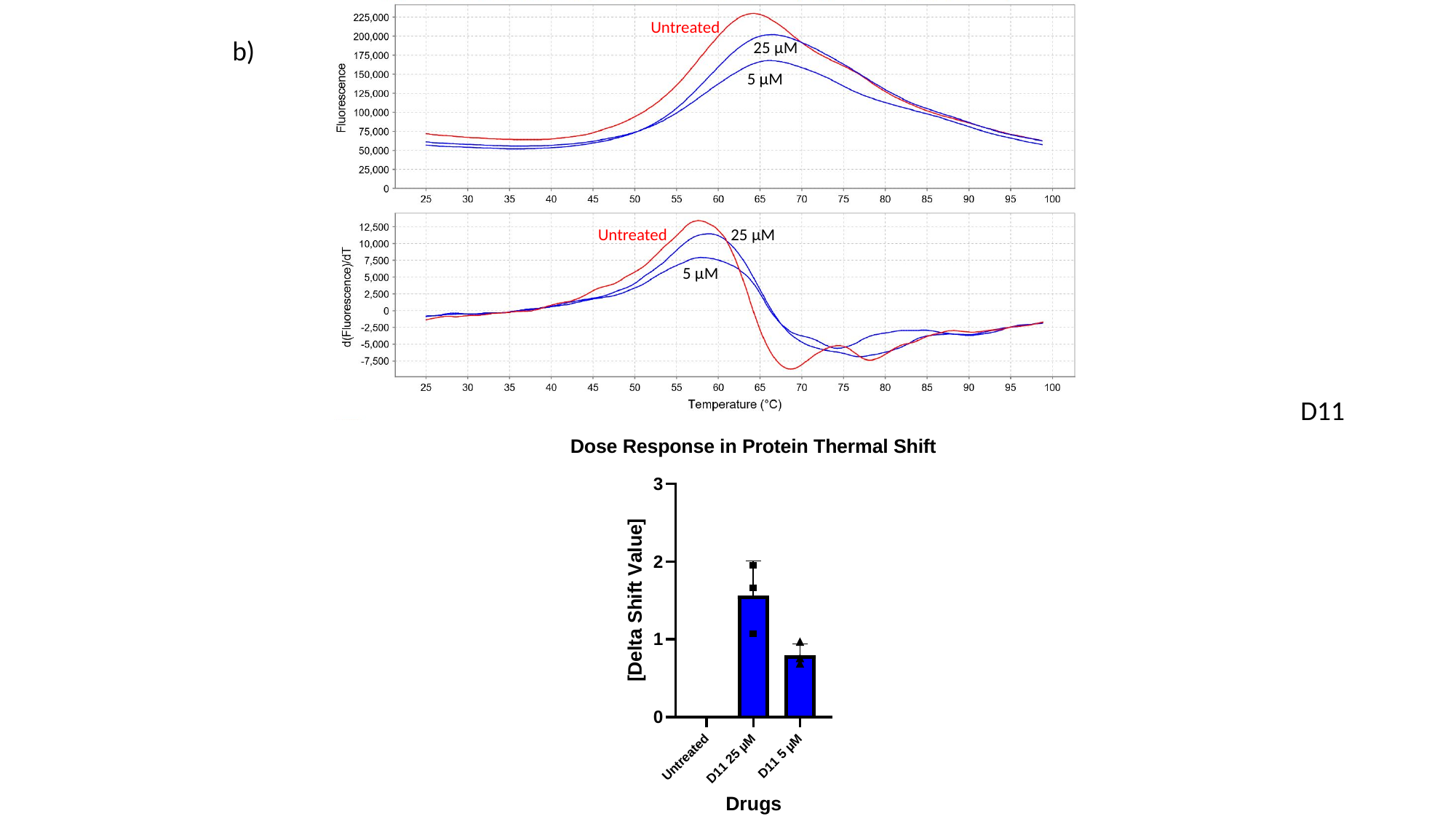

Untreated
b)
25 µM
5 µM
Untreated
25 µM
5 µM
D11

Supplement: Raw Data [file NIHMS1961340-supplement-Raw_Data.zip › RAWData/Figure 1/Figure 1g/Figure 1g ppt.pptx]

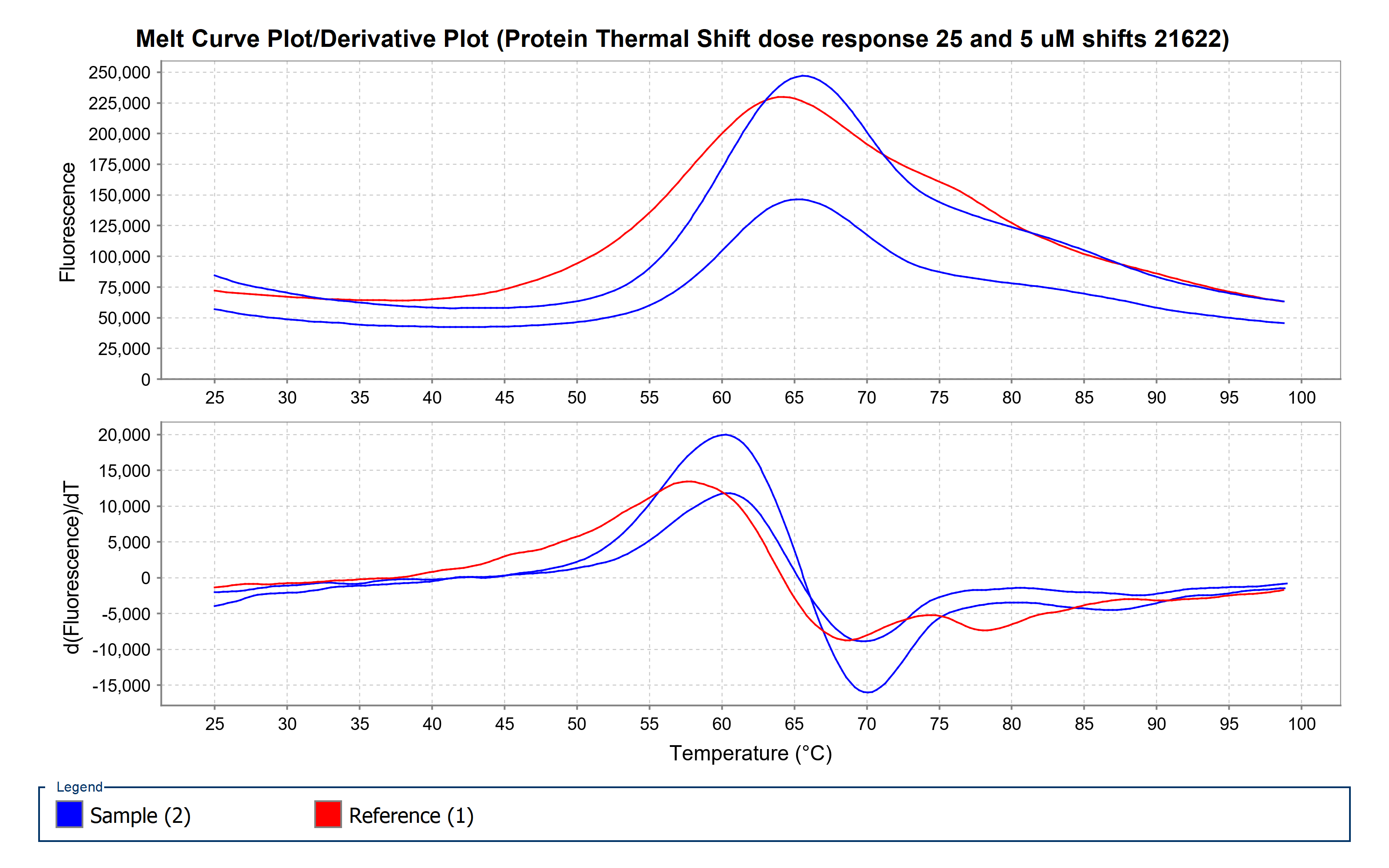

Supplement: Raw Data [file NIHMS1961340-supplement-Raw_Data.zip › RAWData/Figure 1/Figure 1g/Figure 1g.png]

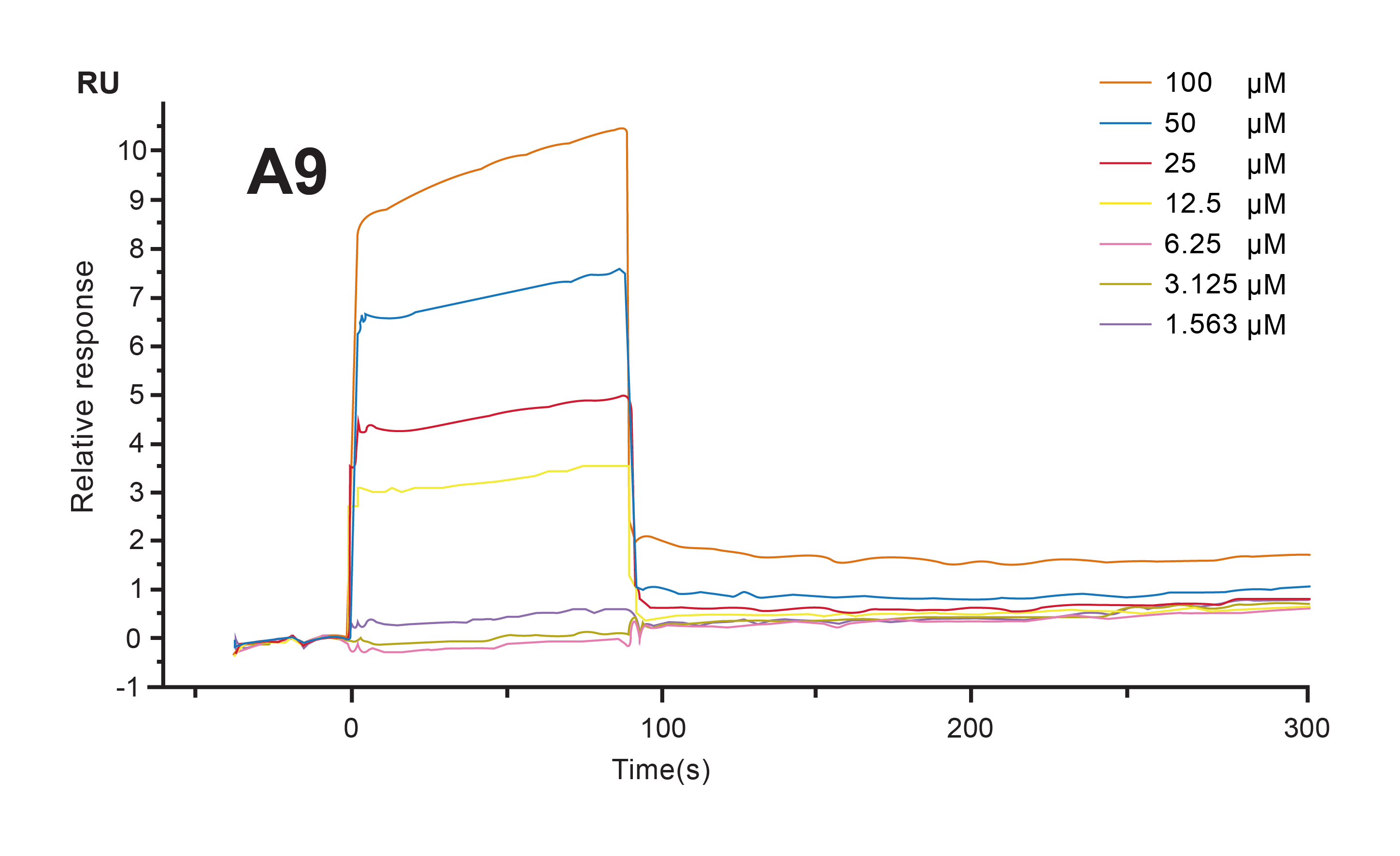

Supplement: Raw Data [file NIHMS1961340-supplement-Raw_Data.zip › RAWData/Figure 1/Figure 1i/Figure 1i A9.png]

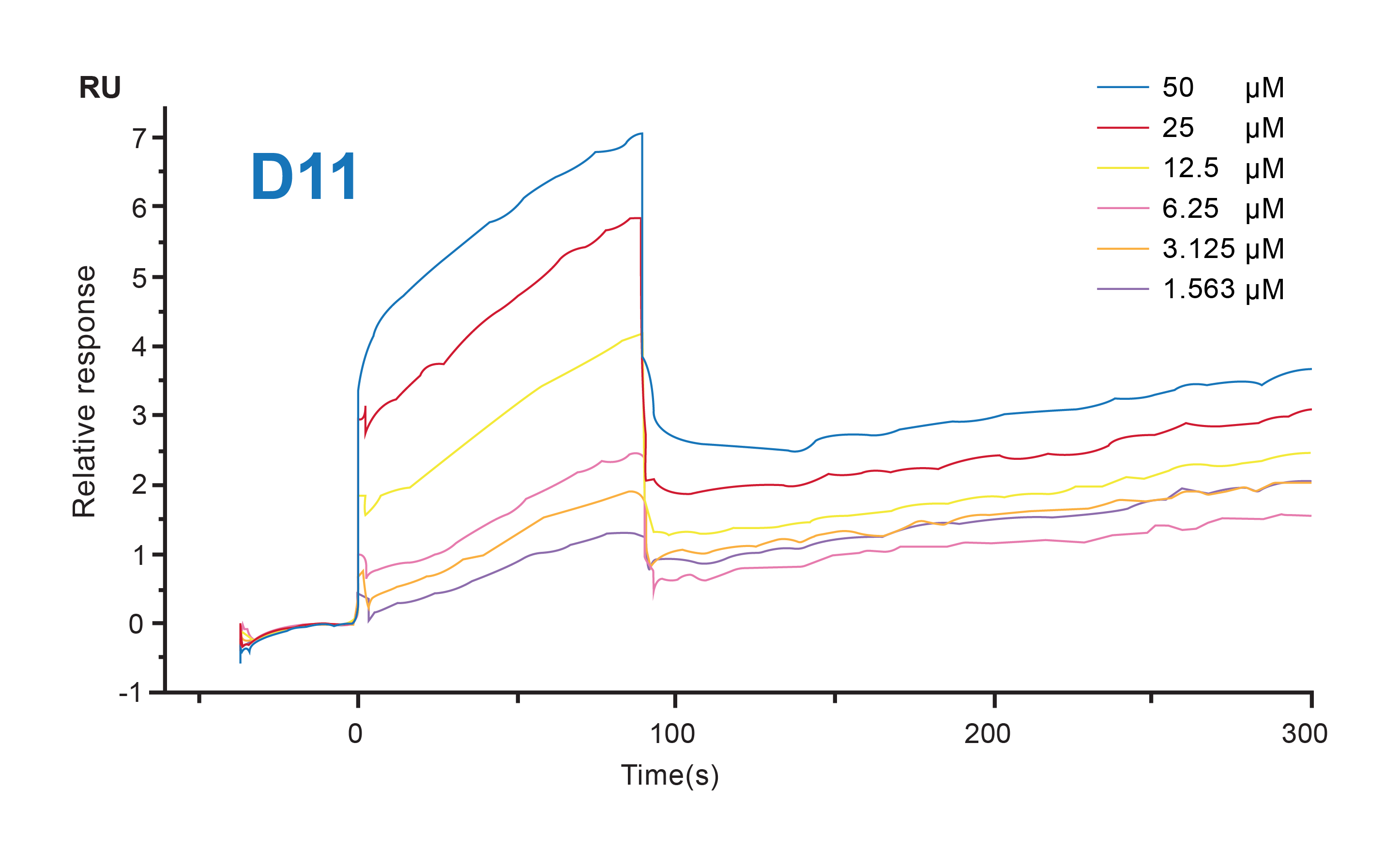

Supplement: Raw Data [file NIHMS1961340-supplement-Raw_Data.zip › RAWData/Figure 1/Figure 1i/Figure 1i D11.png]

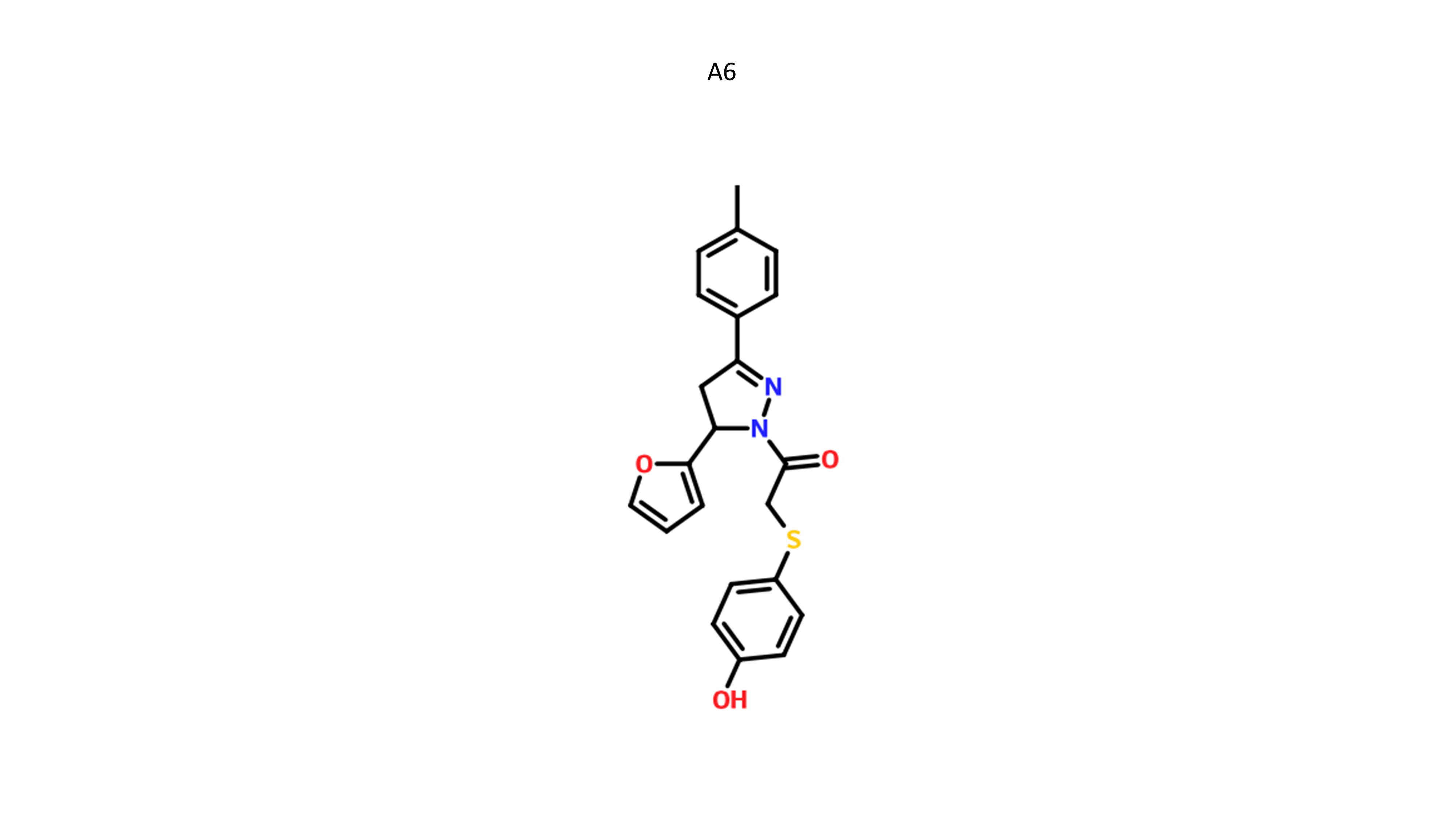

Supplement: Raw Data [file NIHMS1961340-supplement-Raw_Data.zip › RAWData/Figure 1/Figure 1b/Figure 1b chemical structure.TIF]

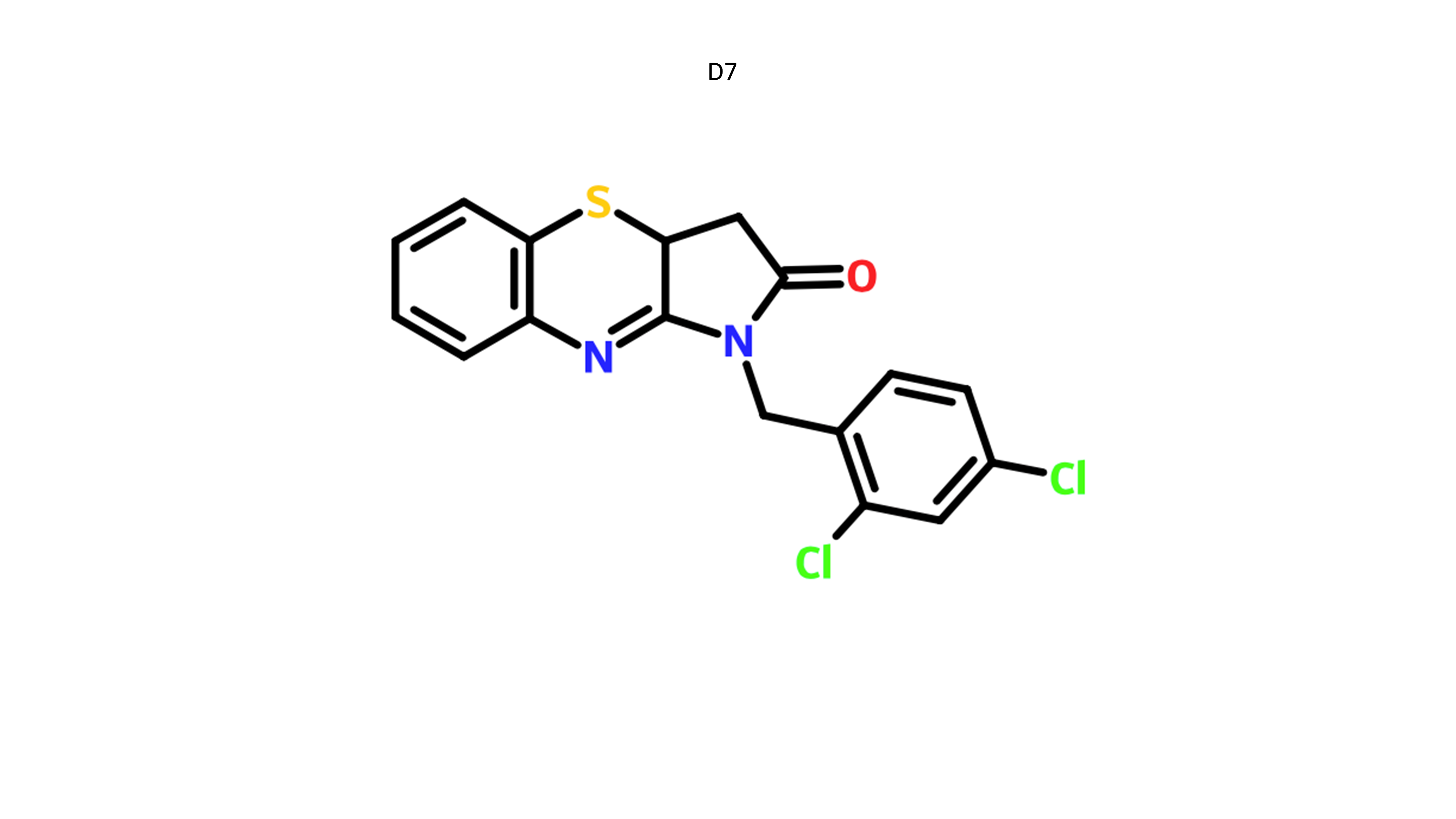

Supplement: Raw Data [file NIHMS1961340-supplement-Raw_Data.zip › RAWData/Figure 1/Figure 1e/Figure 1e chemical structure.TIF]

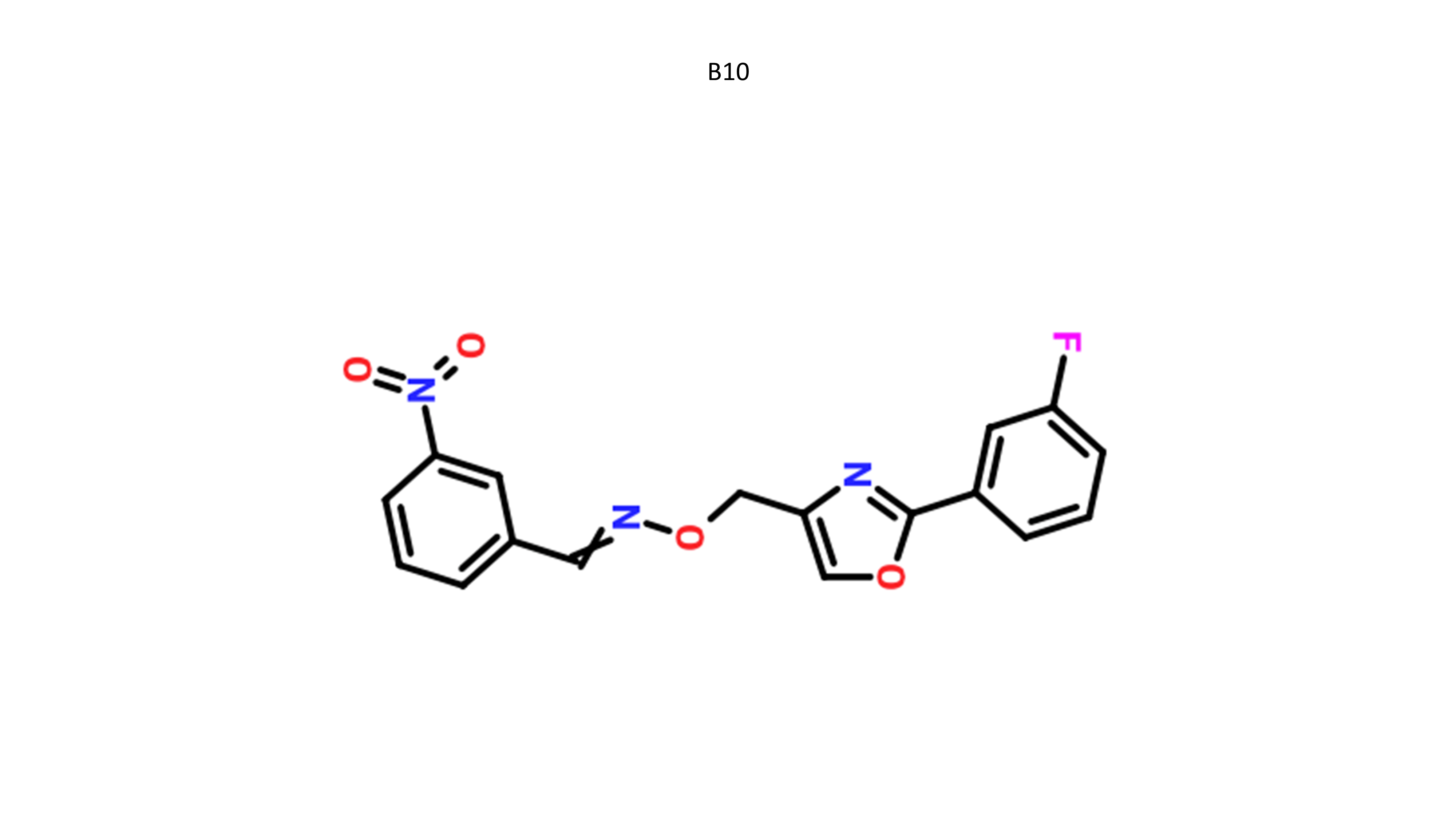

Supplement: Raw Data [file NIHMS1961340-supplement-Raw_Data.zip › RAWData/Figure 1/Figure 1d/Figure 1d chemical structure.TIF]

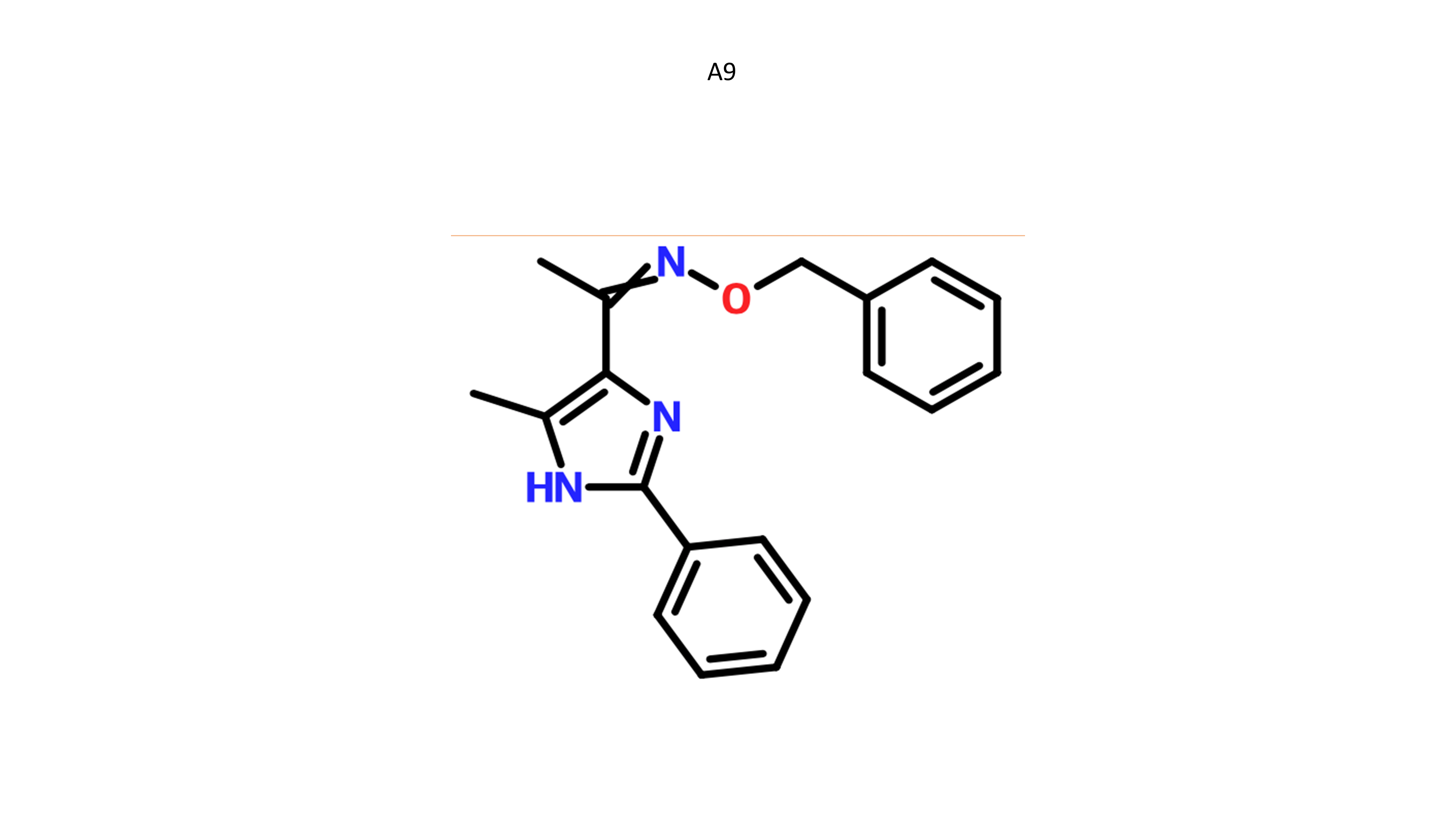

Supplement: Raw Data [file NIHMS1961340-supplement-Raw_Data.zip › RAWData/Figure 1/Figure 1c/Figure 1c chemical structure.TIF]

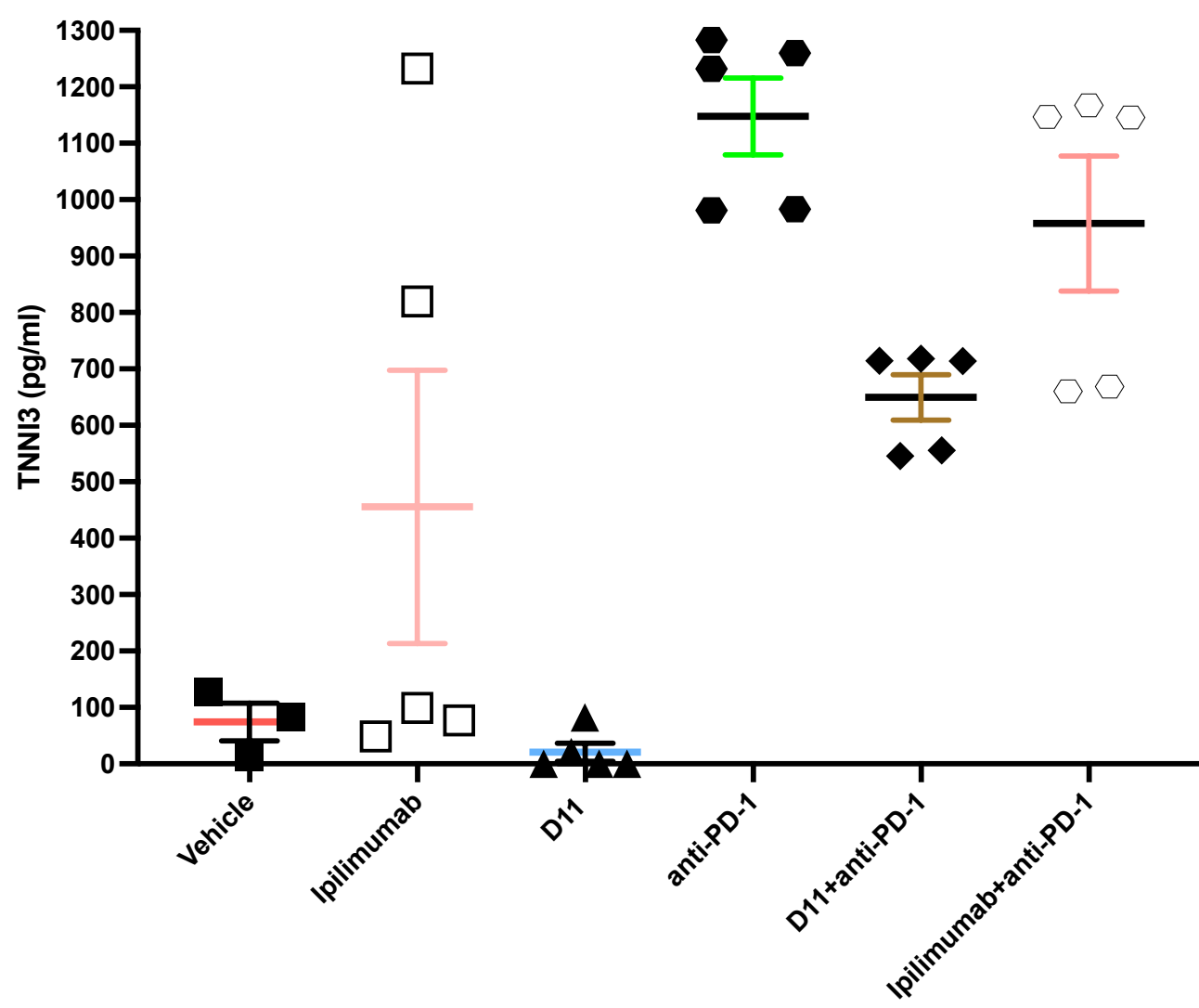

Supplement: Raw Data [file NIHMS1961340-supplement-Raw_Data.zip › RAWData/Figure 4/Figure 4a/TNNI3.pdf]

## Dose-Response IFN- $\gamma$ Stimulation

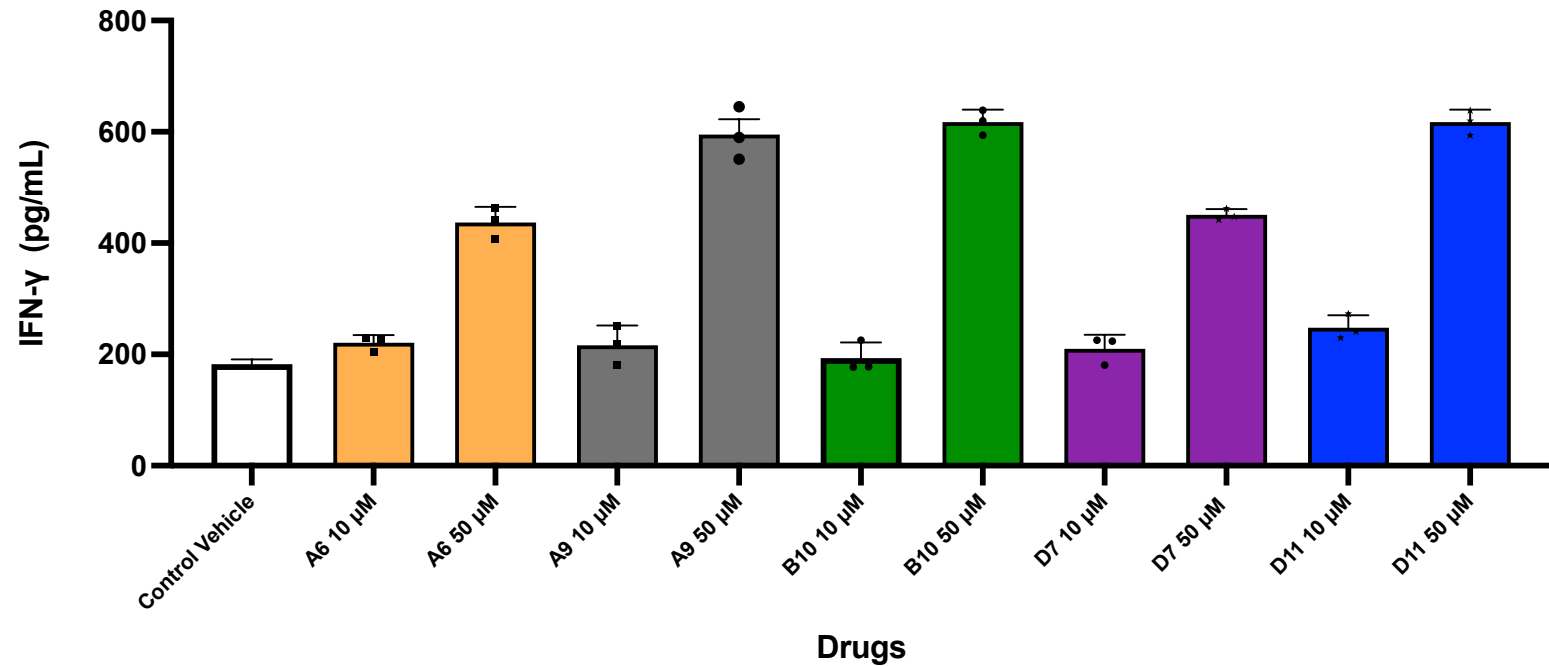

Supplement: Raw Data [file NIHMS1961340-supplement-Raw_Data.zip › RAWData/Figure 2/2a/IFN gamma.pdf]

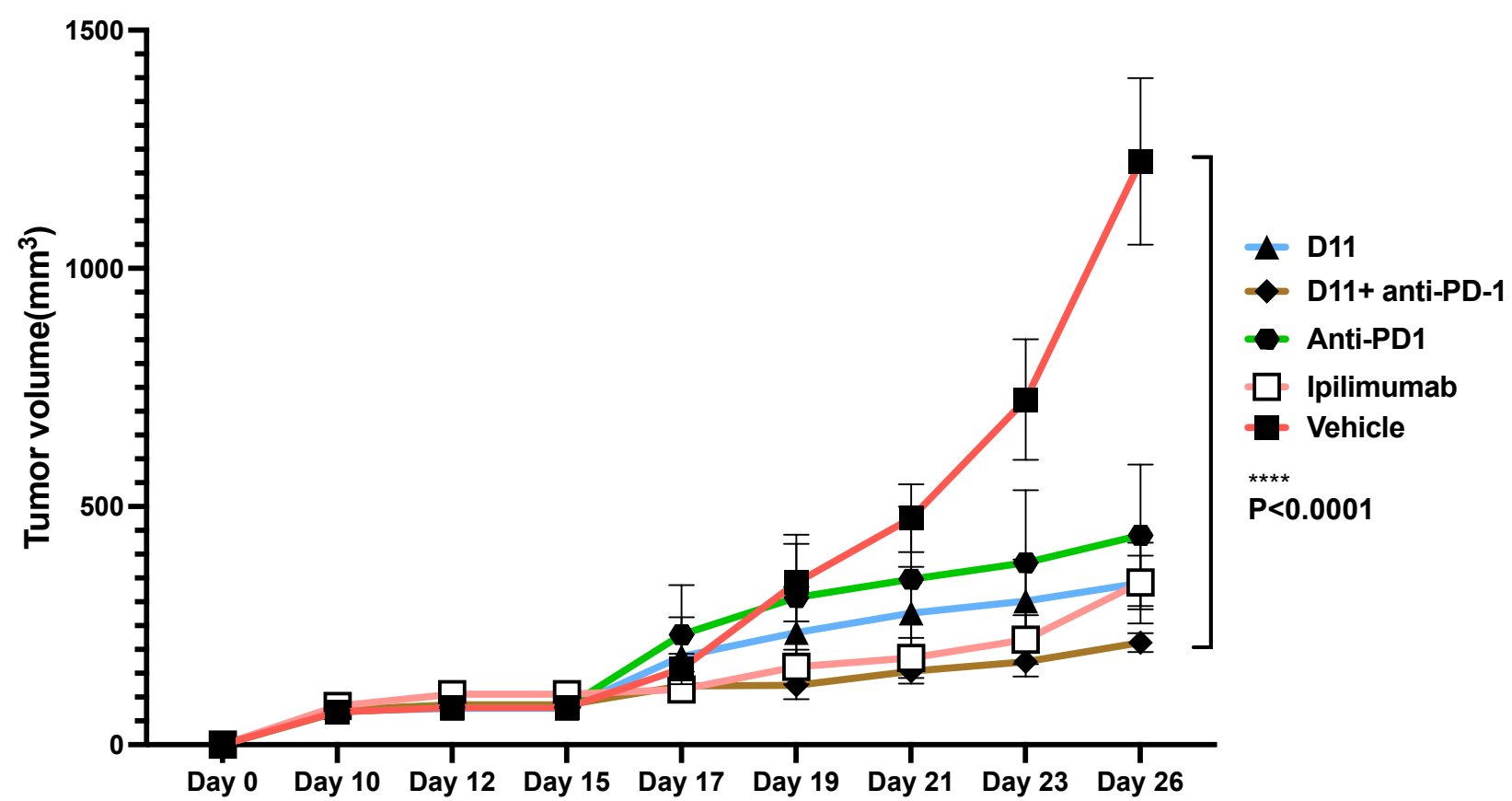

Supplement: Raw Data [file NIHMS1961340-supplement-Raw_Data.zip › RAWData/Figure 5/Figure 5a/Figure 5a.pdf]

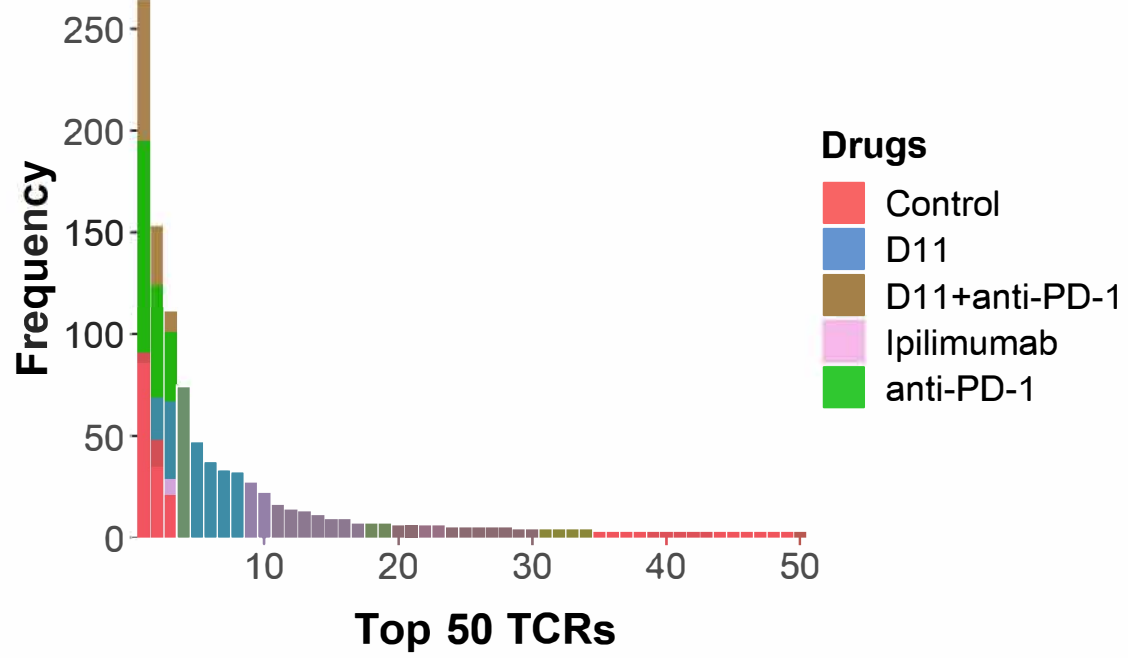

Supplement: Raw Data [file NIHMS1961340-supplement-Raw_Data.zip › RAWData/Figure 5/Figure 5f/Figure 5f.pdf]

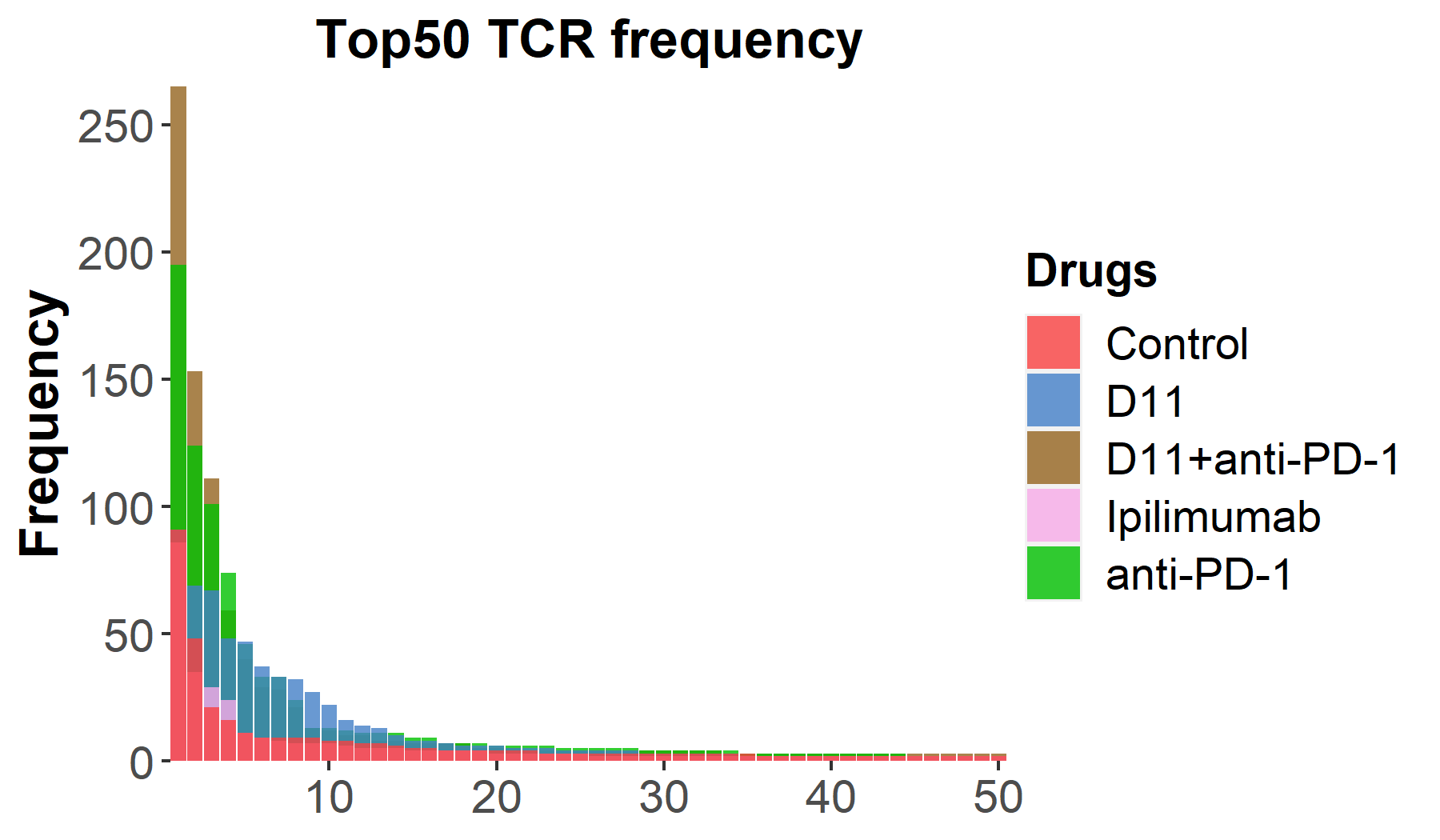

Supplement: Raw Data [file NIHMS1961340-supplement-Raw_Data.zip › RAWData/Figure 5/Figure 5f/Figure 5f.tiff]

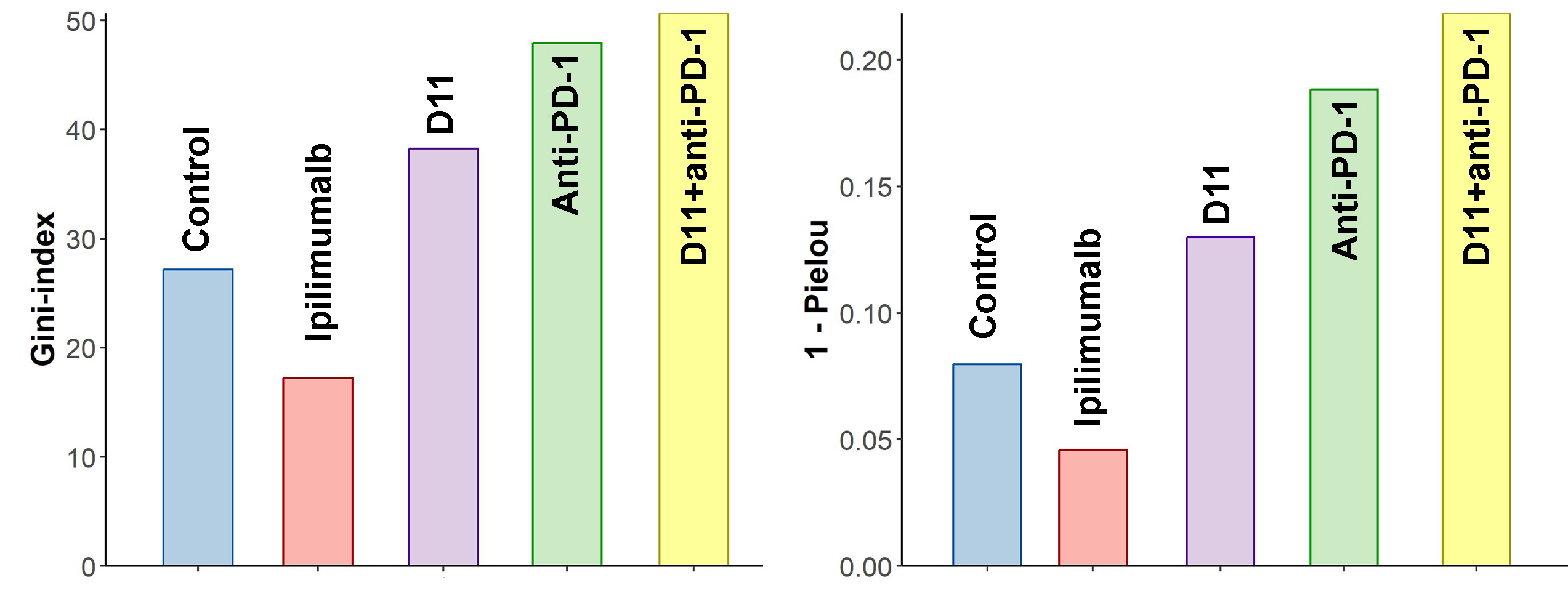

Supplement: Raw Data [file NIHMS1961340-supplement-Raw_Data.zip › RAWData/Figure 5/Figure 5e/Figure 5e.tif]

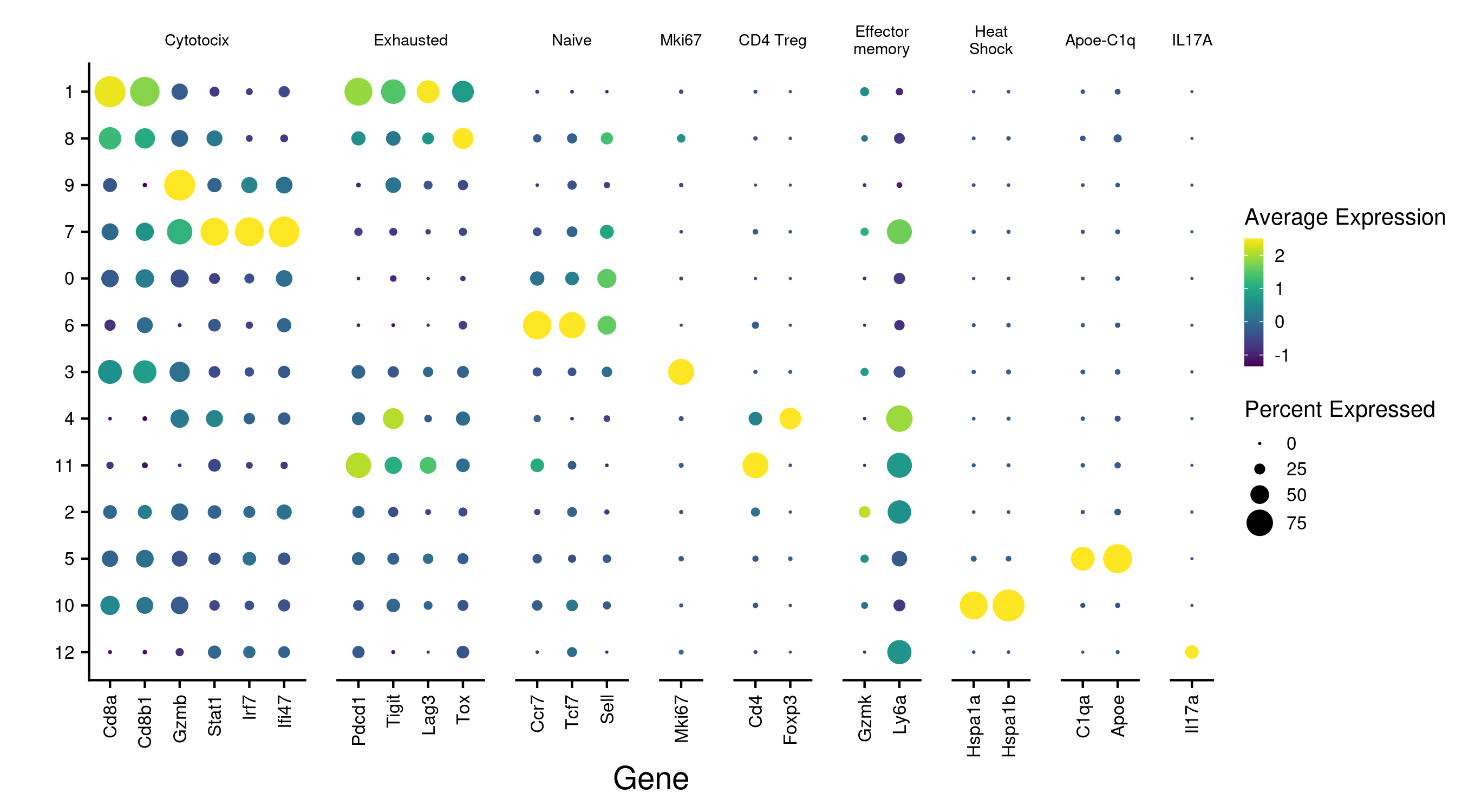

Supplement: Raw Data [file NIHMS1961340-supplement-Raw_Data.zip › RAWData/Figure 5/Figure 5e/Figure 5e subimmune populations from TCR.tif]

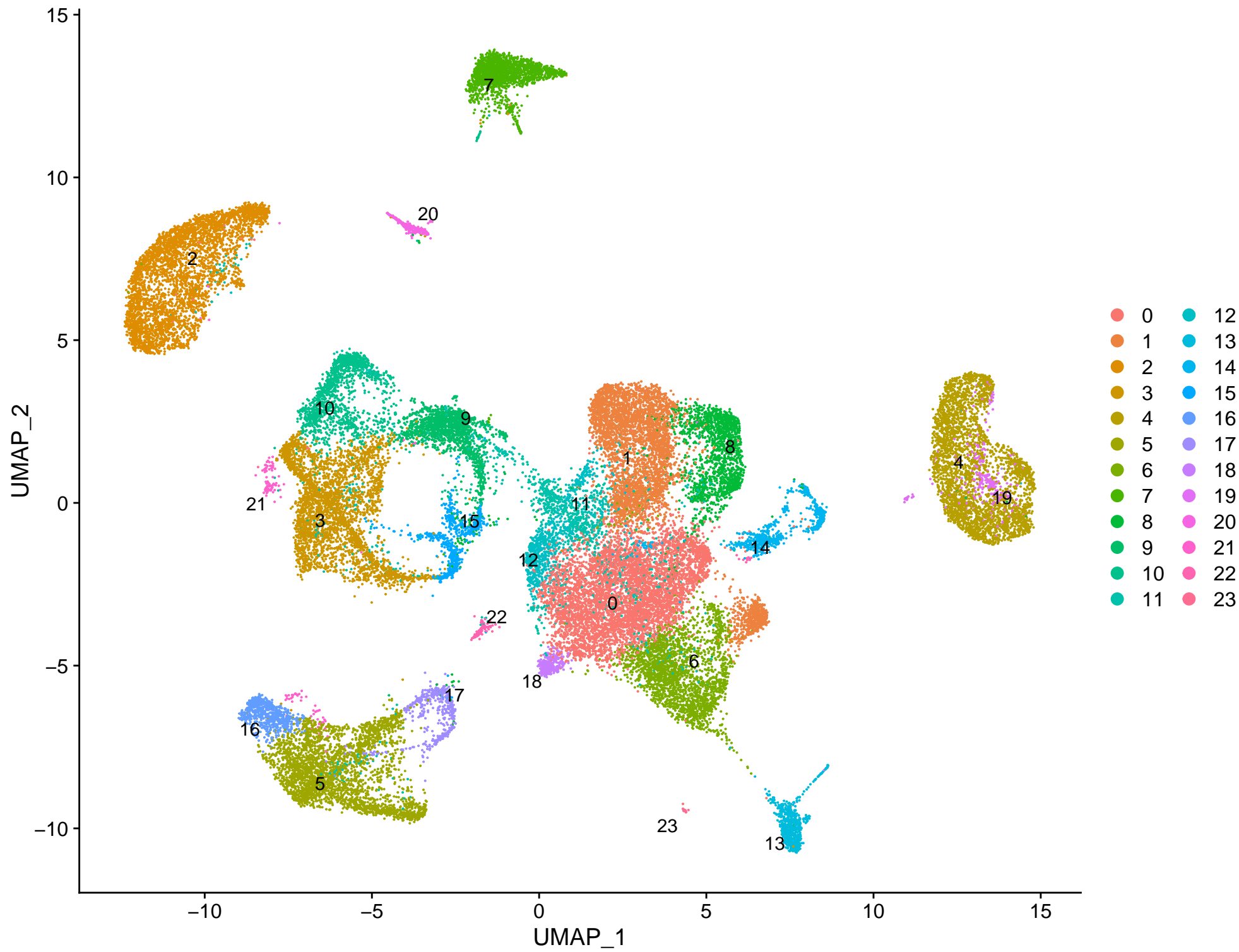

Supplement: Raw Data [file NIHMS1961340-supplement-Raw_Data.zip › RAWData/Figure 5/Figure 5b/Figure 6b supplementary.pdf]

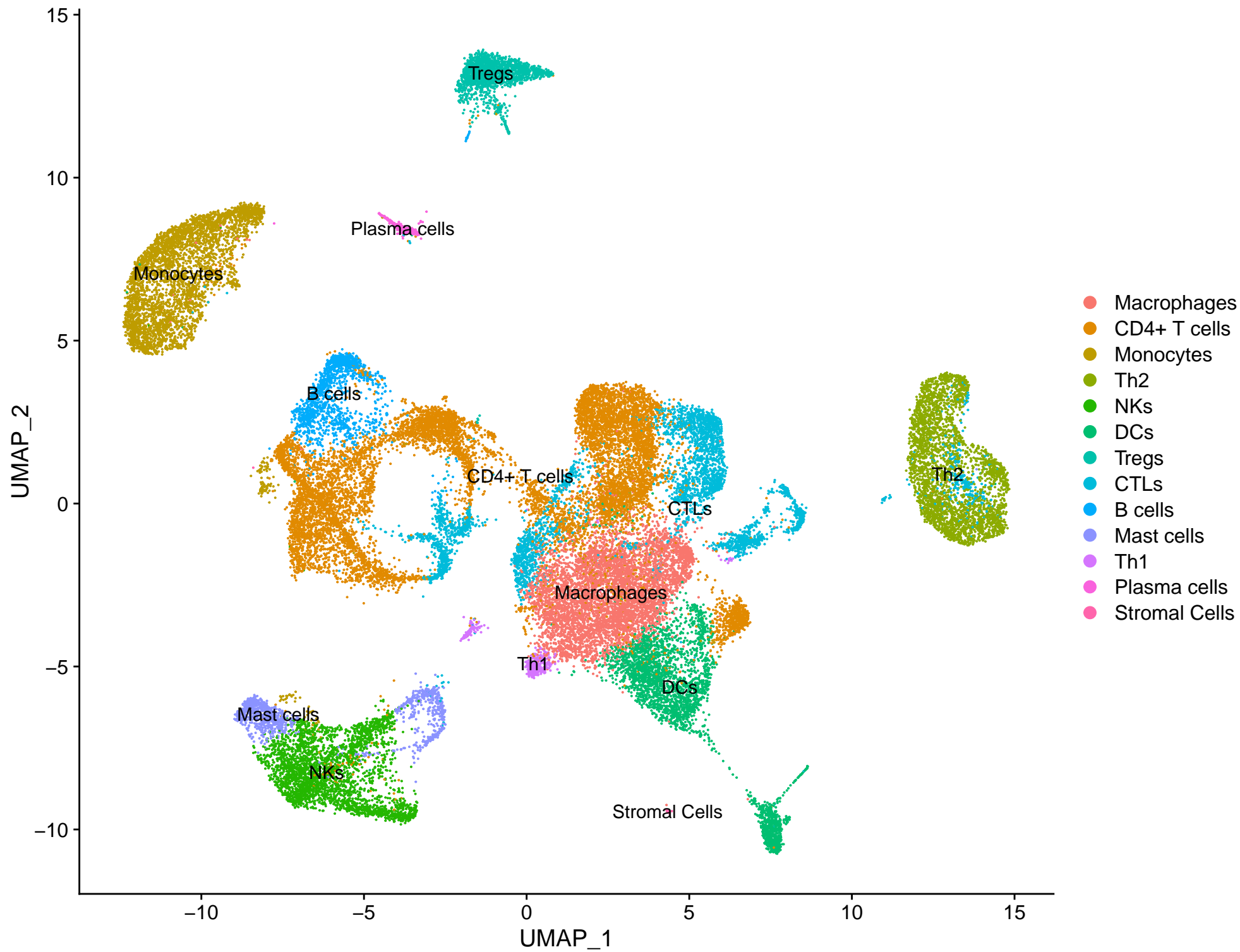

Supplement: Raw Data [file NIHMS1961340-supplement-Raw_Data.zip › RAWData/Figure 5/Figure 5b/Figure 6b reclustering.pdf]

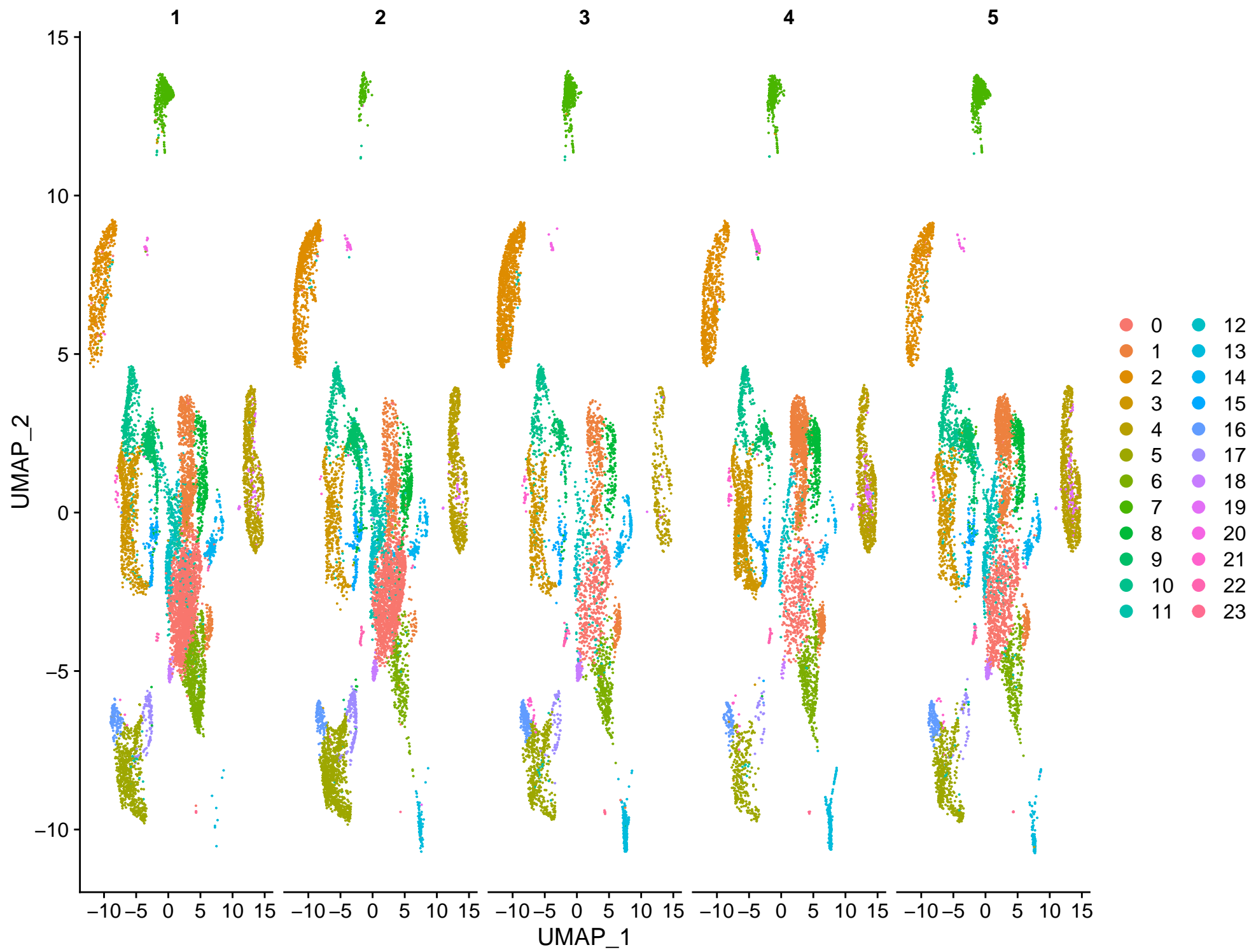

Supplement: Raw Data [file NIHMS1961340-supplement-Raw_Data.zip › RAWData/Figure 5/Figure 5b/Figure 6b supplementary b.pdf]

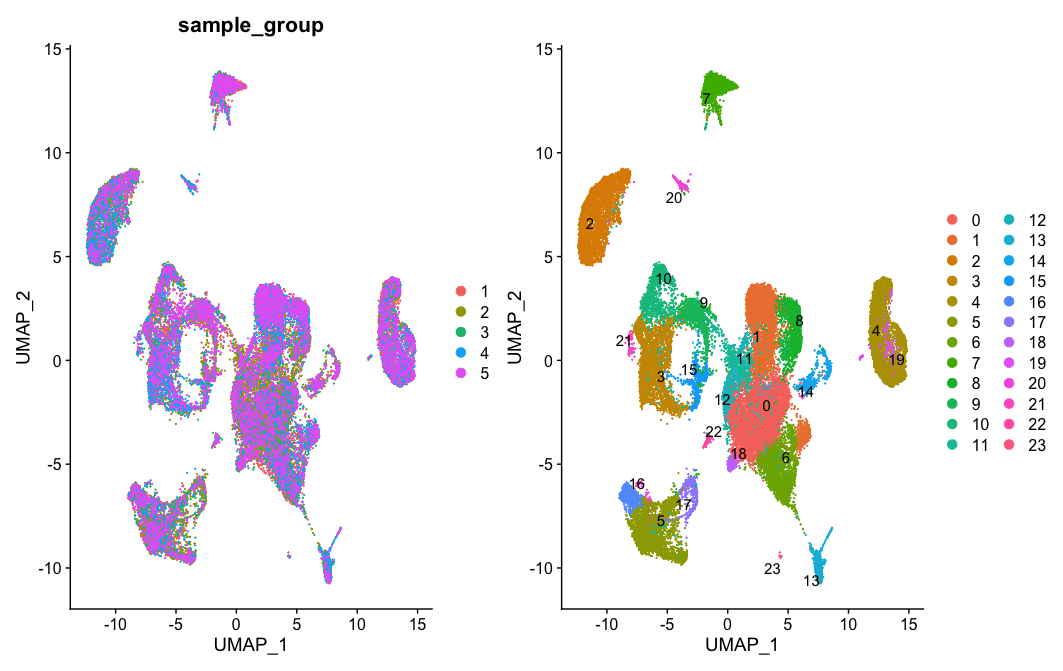

Supplement: Raw Data [file NIHMS1961340-supplement-Raw_Data.zip › RAWData/Figure 5/Figure 5b/Figure 6b 5 colors groups.tiff]

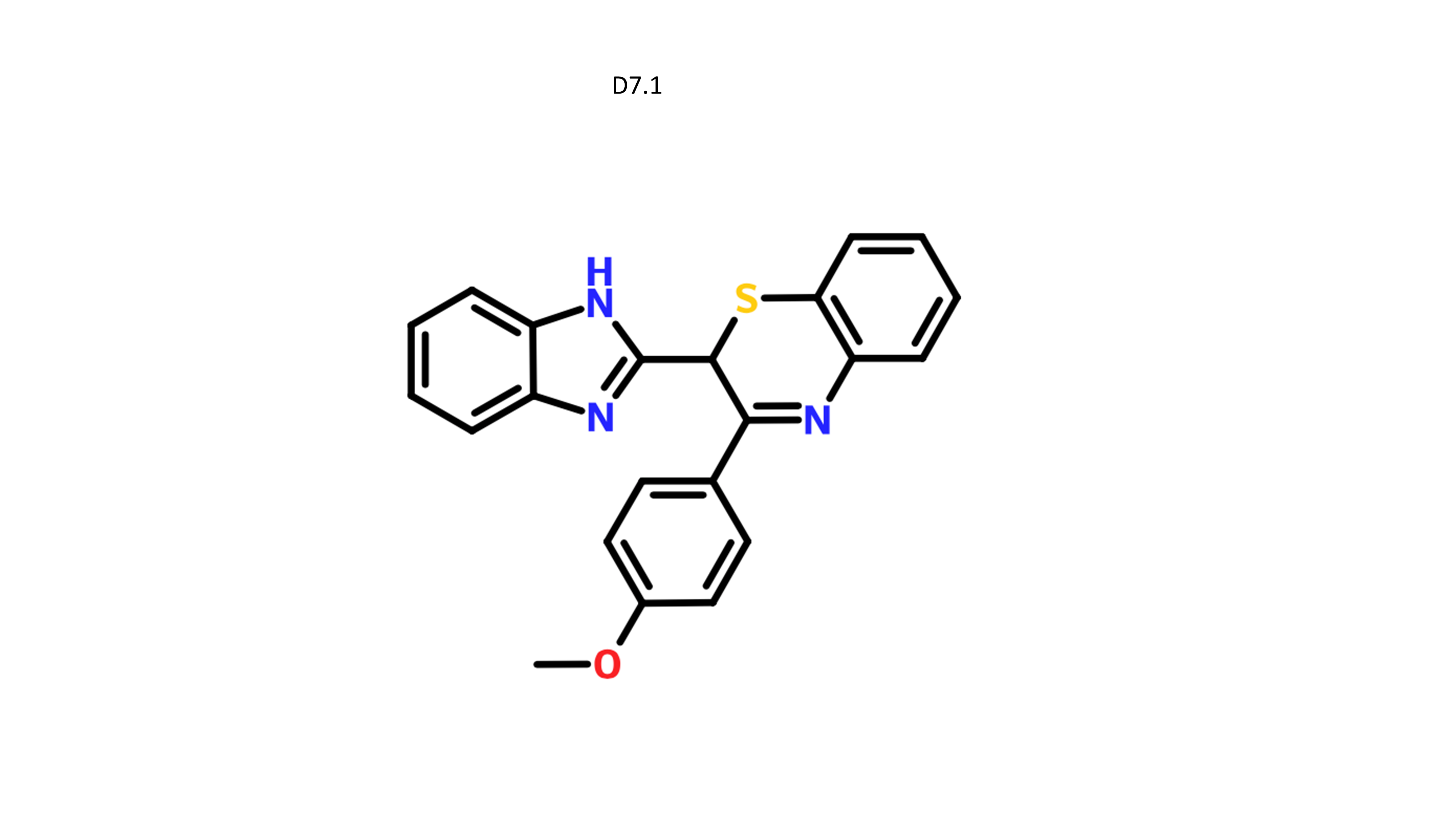

Supplement: Raw Data [file NIHMS1961340-supplement-Raw_Data.zip › RAWData/Figure 6/Figure 6a/Optimized Chemical Structures/D7.1.TIF]

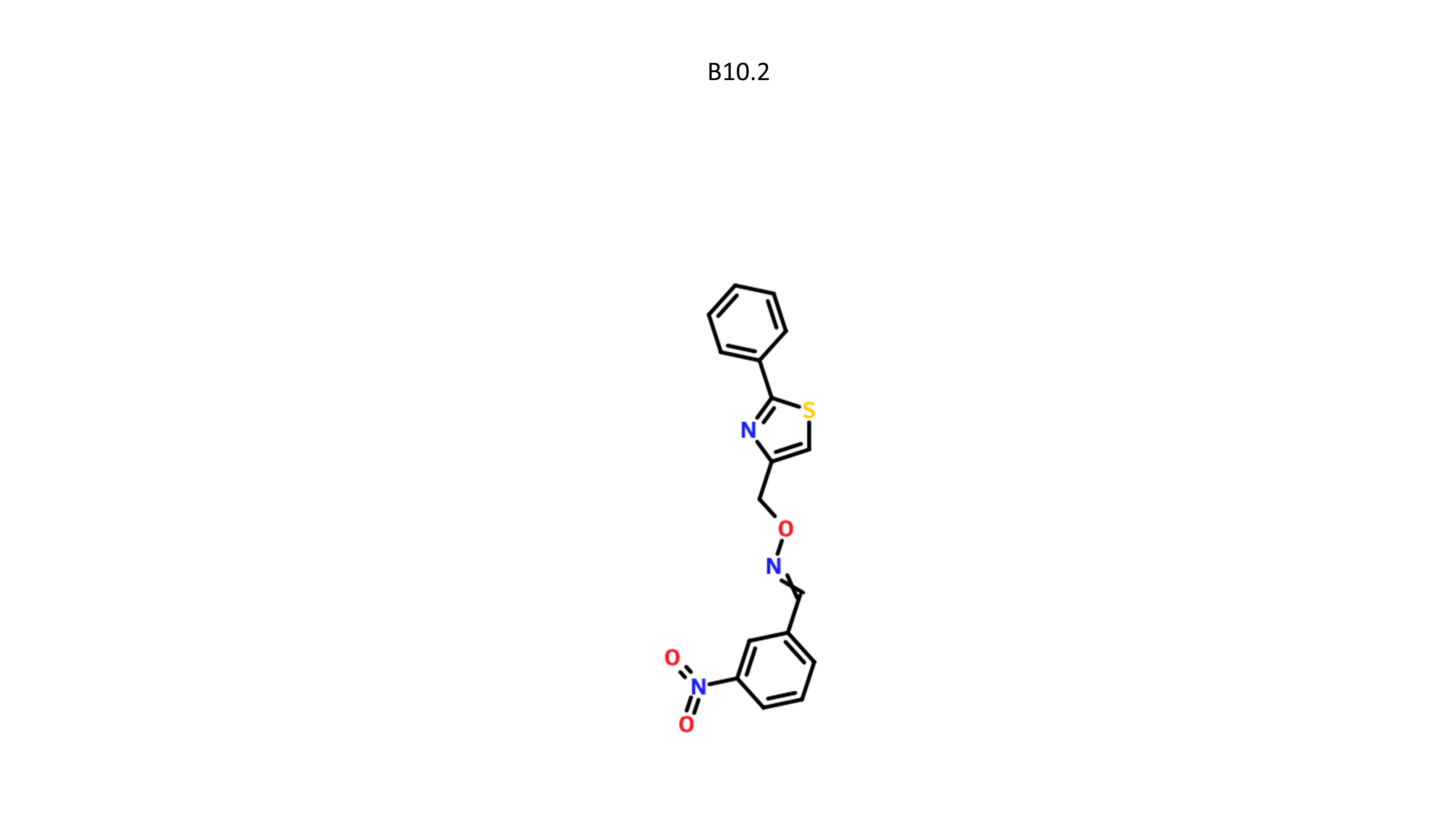

Supplement: Raw Data [file NIHMS1961340-supplement-Raw_Data.zip › RAWData/Figure 6/Figure 6a/Optimized Chemical Structures/B10.2.TIF]

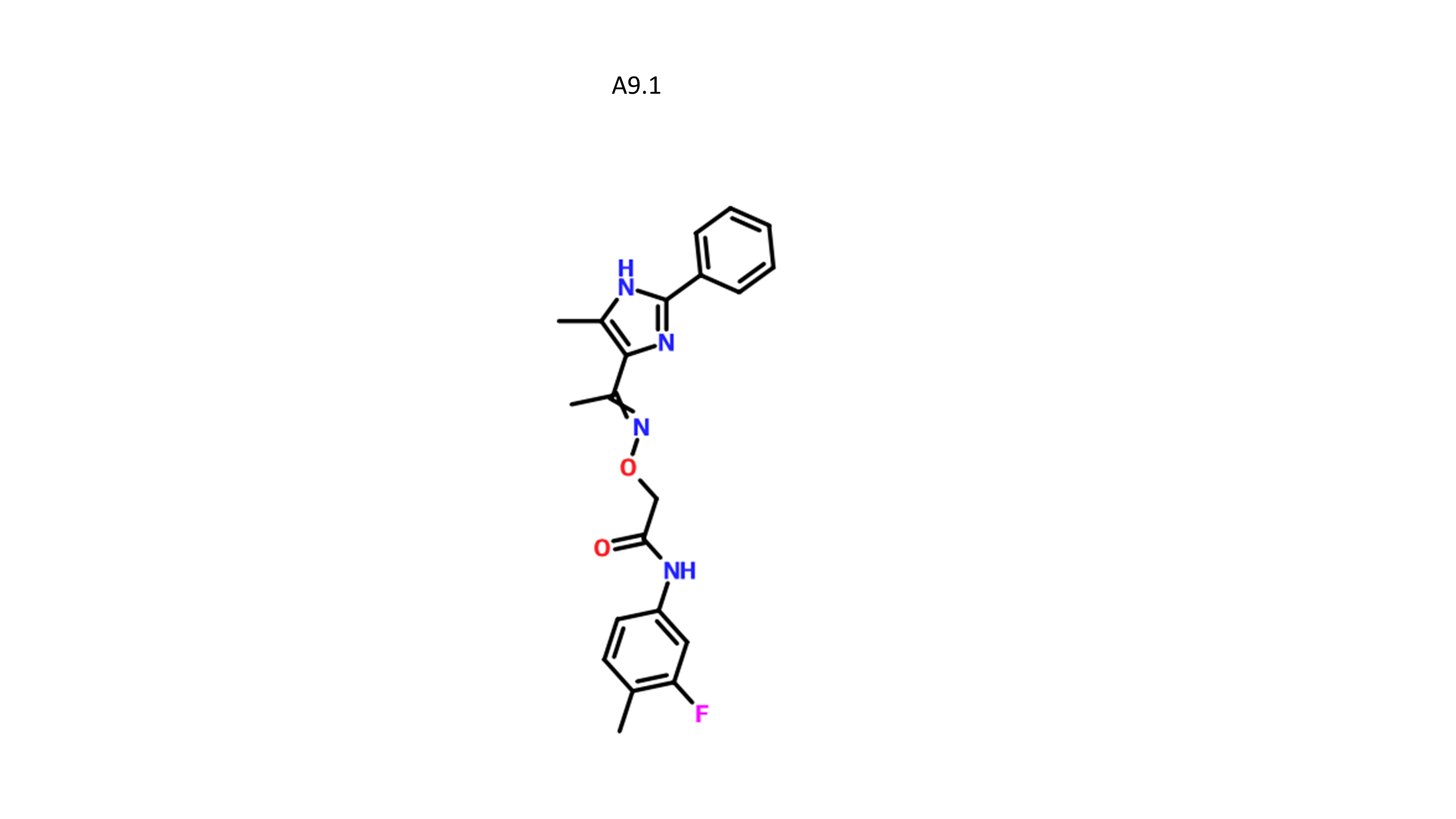

Supplement: Raw Data [file NIHMS1961340-supplement-Raw_Data.zip › RAWData/Figure 6/Figure 6a/Optimized Chemical Structures/A9.1.TIF]

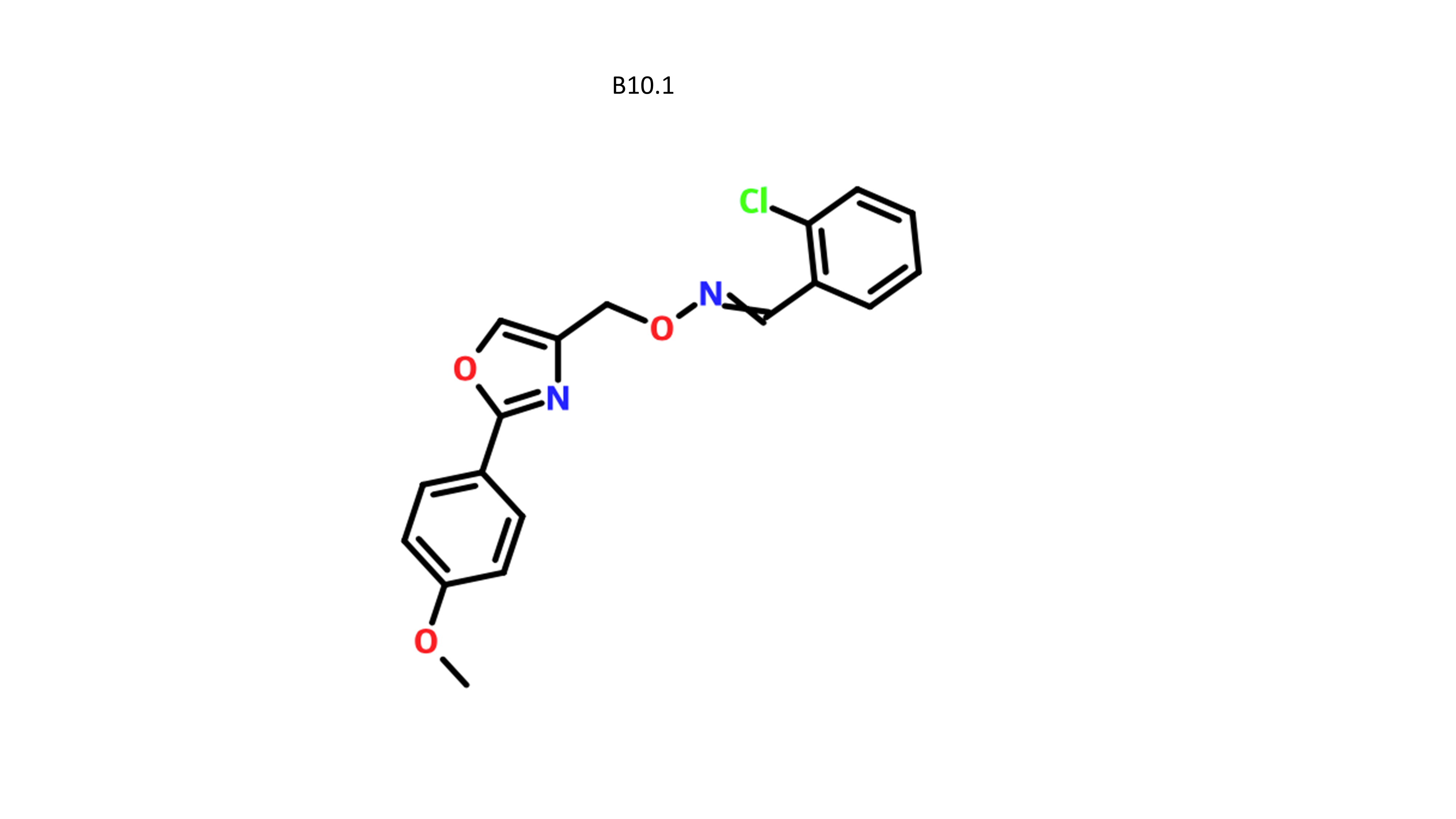

Supplement: Raw Data [file NIHMS1961340-supplement-Raw_Data.zip › RAWData/Figure 6/Figure 6a/Optimized Chemical Structures/B10.1.TIF]

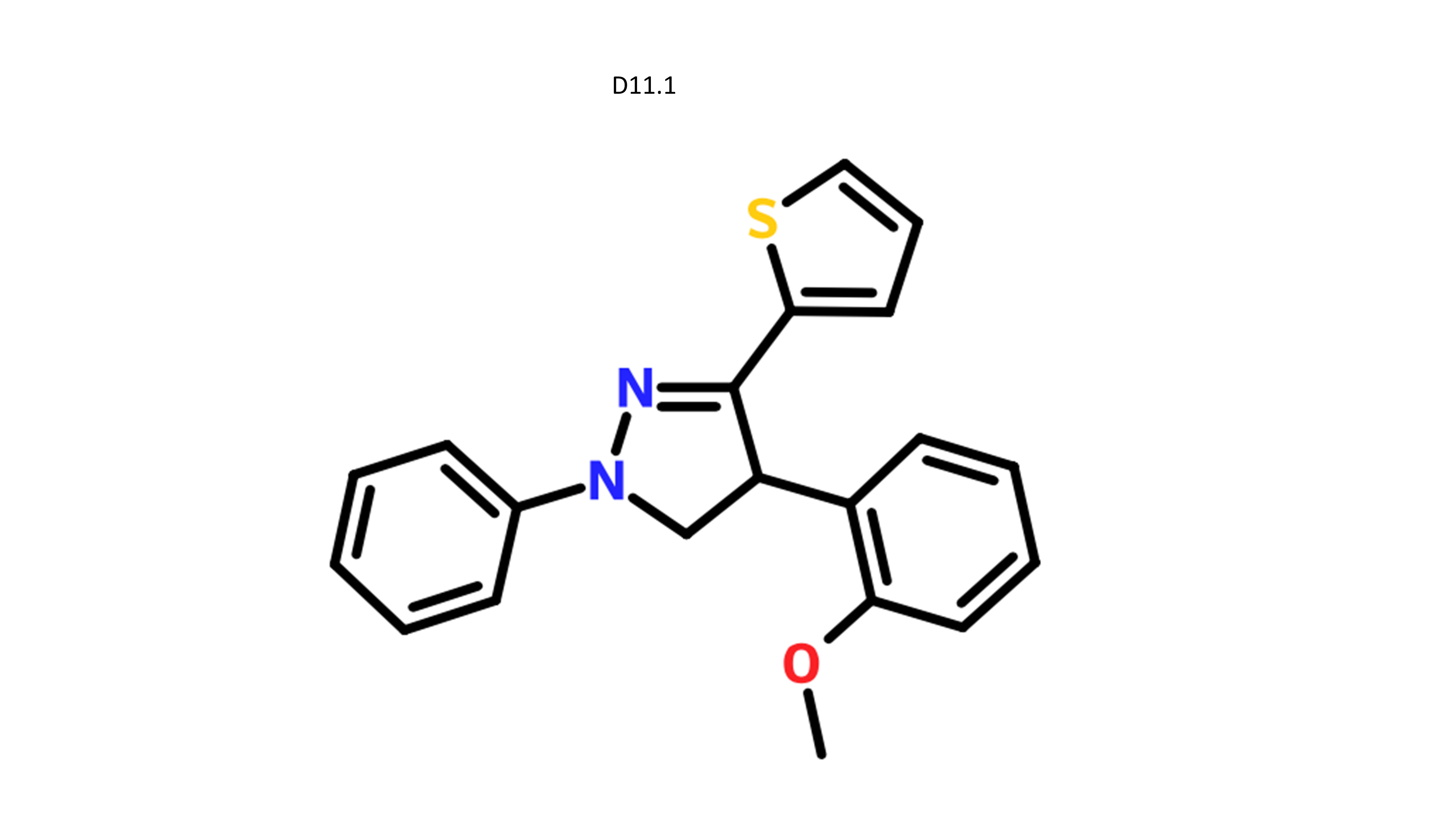

Supplement: Raw Data [file NIHMS1961340-supplement-Raw_Data.zip › RAWData/Figure 6/Figure 6a/Optimized Chemical Structures/D11.1.TIF]

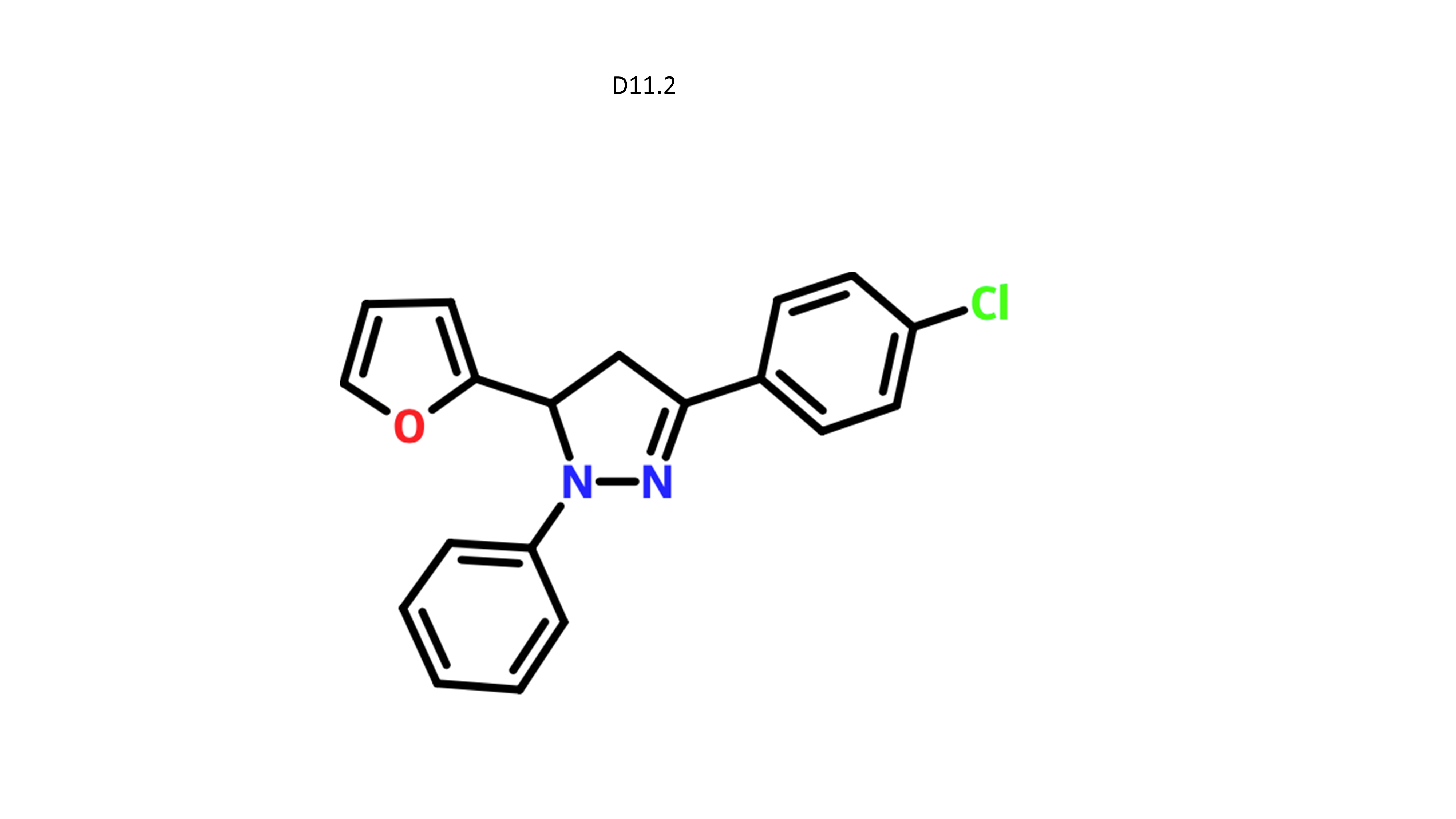

Supplement: Raw Data [file NIHMS1961340-supplement-Raw_Data.zip › RAWData/Figure 6/Figure 6a/Optimized Chemical Structures/D11.2.TIF]

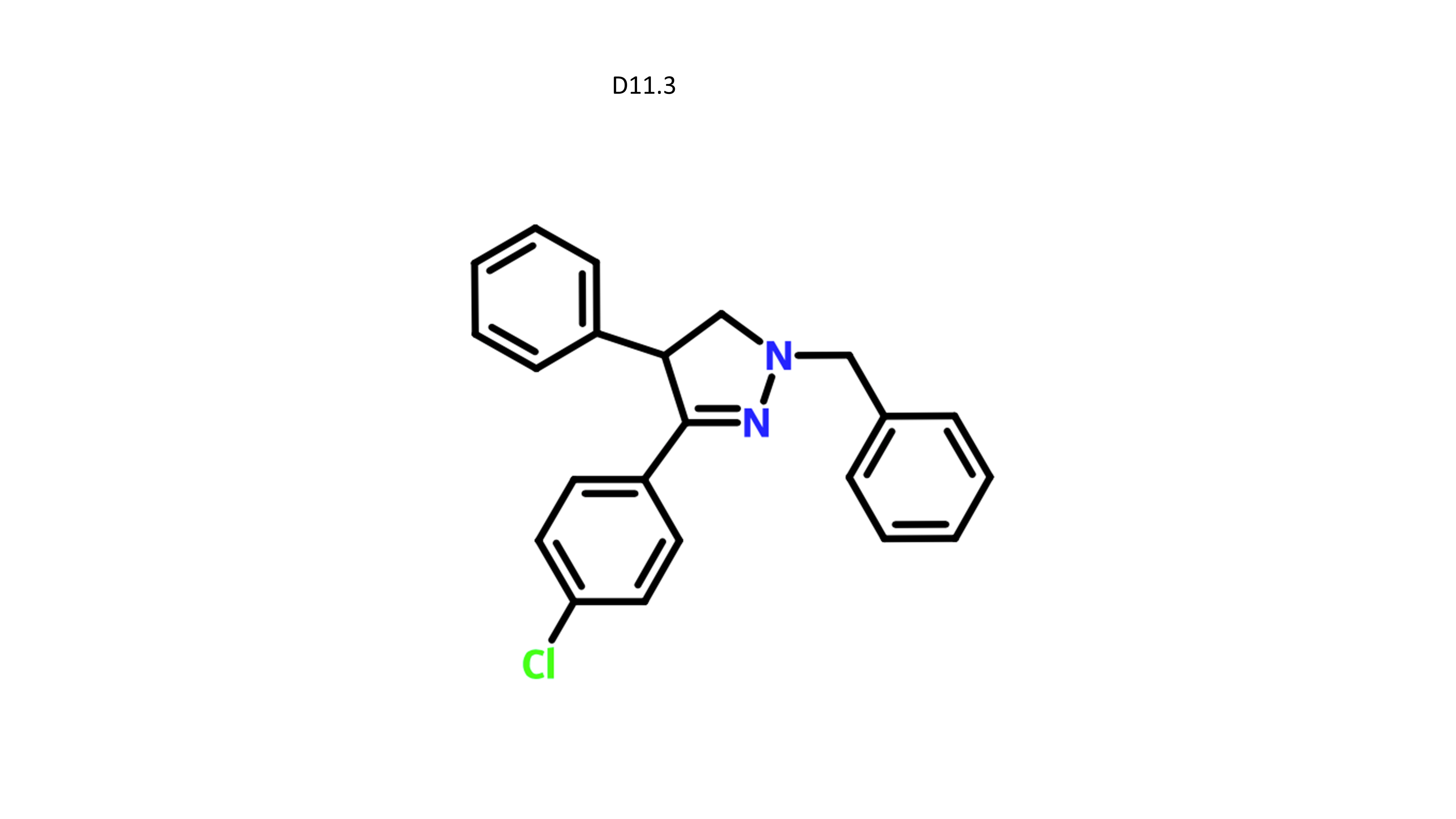

Supplement: Raw Data [file NIHMS1961340-supplement-Raw_Data.zip › RAWData/Figure 6/Figure 6a/Optimized Chemical Structures/D11.3.TIF]

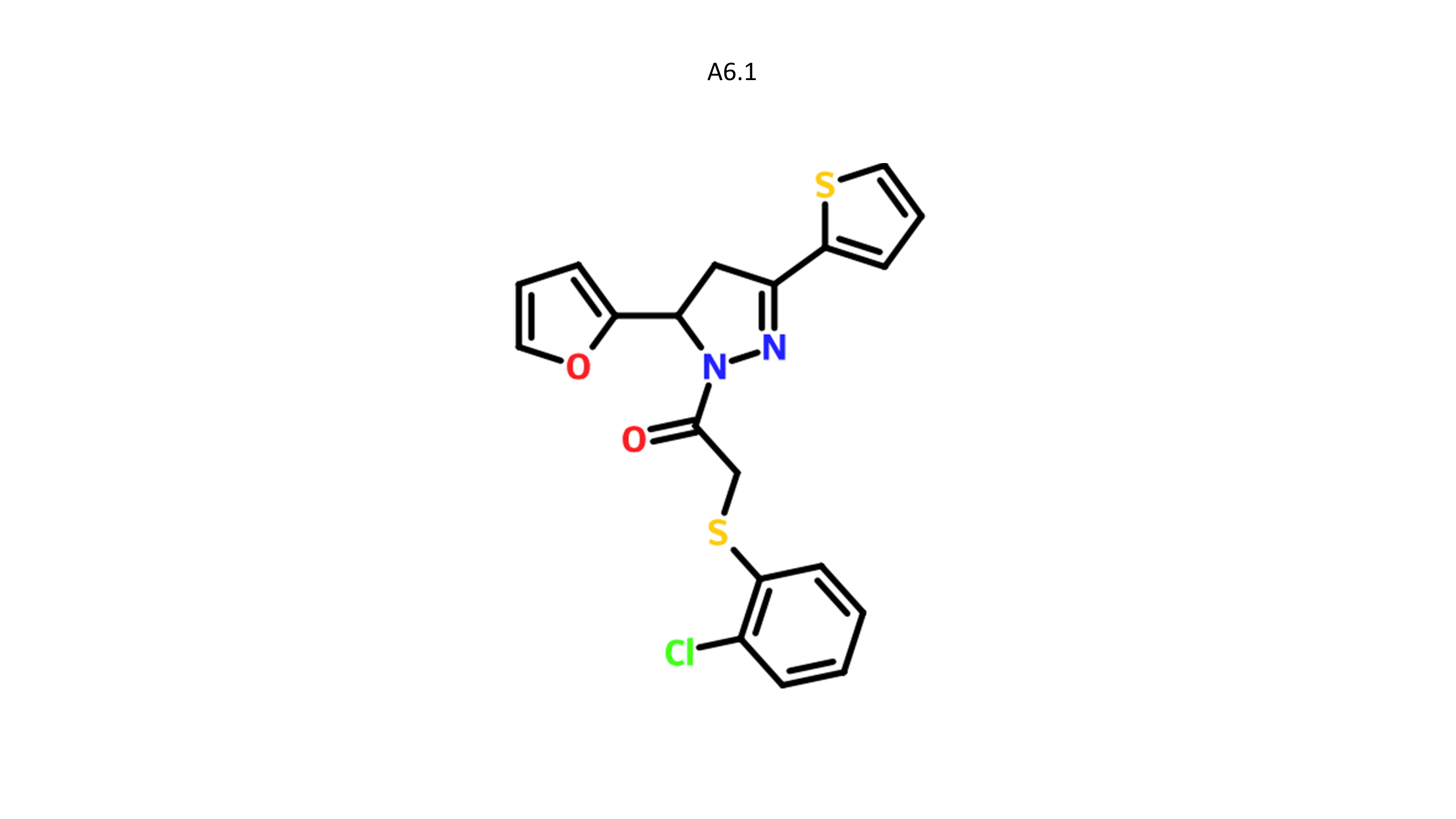

Supplement: Raw Data [file NIHMS1961340-supplement-Raw_Data.zip › RAWData/Figure 6/Figure 6a/Optimized Chemical Structures/A6.1.TIF]
