# Supplementary material for: Artificial intelligence-powered discovery of small molecules inhibiting CTLA-4 in cancer
Source: BJC Rep. 2024 Jan 23;2:4. doi: 10.1038/s44276-023-00035-5 (PMC10838660; doi:10.1038/s44276-023-00035-5)
Supplement: Supplementary file 1 [file 44276_2023_35_MOESM2_ESM.docx]

**Supplementary Figure Legends:**

**Supplementary Figure S1. Cell-surface hCTLA-4 protein displacement and luciferase reporter bioassay for anti-CTLA-4 lead compounds**. **(a, b)** hCTLA-4 protein displacement for lead compounds. Jurkat hCTLA-4–overexpressing cells were treated with lead compounds to investigate hCTLA-4 protein displacement with flow cytometry. The lead compounds were incubated together with the cells for 45 min or 24 h, respectively. Ipilimumab and unstained, untreated cells were used as controls. **(c, d)** hCTLA-4 protein displacement for the optimized compounds. Jurkat hCTLA-4–overexpressing cells were treated with the top optimized compounds to investigate hCTLA-4 protein displacement with flow cytometry. The optimized compounds were incubated together with the cells for 45 min or 24 hours, respectively. Ipilimumab and unstained, untreated cells were used as controls. **(e)** Promega luciferase reporter assay. CTLA-4 targeting compounds inhibited CTLA-4 in human T lymphocytes. The luciferase reporter was driven by a CD28 pathway–dependent promoter. The Raji cells expressed CD80/86 on their surfaces. The effector Jurkat T cells expressed CD28 and CTLA-4 on their surfaces. CTLA-4 competed with the costimulatory molecule CD28 for the CD80/86 ligands, for which it has a higher affinity and avidity. Following inhibition of CTLA-4, the CD80/86 ligands binded to CD28, activating the luciferase promoter. **(f)** The inhibition of interactions between hCTLA-4 and CD80/86 by the top 5 lead compounds in the luciferase reporter assay. APC, antigen-presenting Cell; CTLA-4, cytotoxic T-lymphocyte–associated protein 4; hCTLA-4, humanized CTLA-4; RLU, relative light unity.

**Supplementary Figure S2.** Surface plasmon resonance binding curves for the compounds D7, B10, and A6. 

**Supplementary Figure S3. T conventional and T regulatory cells treated with CTLA-4 inhibitors. (a)** T_conv_ cells. Dendritic cells and allogenic T-cells from human PBMCs were cocultured with the compounds at 2 doses (10 µM and 50 µM) and T-cells were determined with flow cytometry. **(b)** The ratio of human T_conv_ to T_reg_ cells was also determined with flow cytometry (n = 3). **(c)** Mouse T_reg_ cells were measured in the tumors of C57BL/6 mice who had received 25 mg/kg of A9 or D11 treatment. Ipilimumab and phosphate-buffered saline (vehicle) were used as controls. **(d)** Mouse T_reg _comparisons between treatment groups. CTLA-4, cytotoxic T-lymphocyte–associated protein 4; Foxp3, forkhead box p3; FSC, forward scatter; FSC-A, forward scatter A; PBMC, peripheral blood mononuclear cell; SSC, side scatter; SSC-A, side scatter A; T_conv_, T conventional; T_reg_, T regulatory cell.

 

**Supplementary Figure S4. Tumor inhibition in C57BL/6 mice.** **(a, b)** Tumor prevention in individual mice. D11 and A9 prevented MC38 tumor growth. The compounds D11 or A9 or the phosphate-buffered saline (vehicle) control were injected into animals (n = 10 for D11, n = 10 for A9, and n = 5 for the vehicle) intraperitoneally before the tumors were established. The 4 mice that survived the A9 treatment were further challenged with 200,000 MC38 cells. **(c, d)** Tumor inhibition in individual mice. D11 and A9 inhibited MC38 tumors. Treatments (n = 5 for D11, n = 7 for A9, and n = 7 for the vehicle) were started after the tumors were established, and the tumor volumes reached approximately 100 mm^3^. 

**Supplementary Figure S5****. Tumor inhibition in humanized CTLA-4 C57BL/6 mice.** Tumor inhibition in individual mice. D11 and A9 inhibited MC38 tumors similarly to ipilimumab. Treatments (n = 5 for D11, n = 5 for A9, and n = 4 for the phosphate-buffered saline (vehicle)) were started after the tumors were established, and the tumor volumes reached approximately 100 mm^3^. 

**Supplementary Figure S6. Cell viability reduction in the HCT-116 and MDA-MB-231 cell lines.**. Unlike 5-fluorouracil, D11 did not kill cancer cells directly when added to HCT-116 and MDA-MB-231 cells. A phosphate-buffered saline (vehicle) was used as a normalization control.

**Supplementary Figure S7. Tumor inhibition in individual humanized CTLA-4 C57BL/6 mice.** D11 inhibited MC38 tumors similarly to ipilimumab, anti-PD-1, and D11 plus anti-PD-1. Treatments (n = 5 for D11, n = 4 for anti-PD-1, n = 5 for D11 plus anti-PD-1, n = 5 for ipilimumab, and n = 4 for the phosphate-buffered saline (vehicle)) were started after the tumors were established, and the tumor volumes reached approximately 100 mm^3^). The data for the mice with tumors that were used for single-cell sequencing are labeled with stars. PD-1, programmed cell death protein-1.

**Supplementary Figure S8. Cell surface hCTLA-4 protein displacement and luciferase reporter bioassay results for anti-CTLA-4 lead compounds and tumor inhibition**. **(a, b)** hCTLA-4 protein displacement for optimized compounds. Jurkat hCTLA-4–overexpressing cells were treated with the lead compounds to investigate hCTLA-4 protein displacement with flow cytometry. The lead compounds were incubated together with the cells for 45 min or 24 h, respectively. ipilimumab and unstained, untreated cells were used as controls. **(c-d)** Tumor inhibition in individual mice. A9.1 and D11.3 inhibited MC38 tumors. Treatments (n = 11 for A9.1, n = 5 for D11.3, and n = 10 and n = 6 for the phosphate-buffered saline (vehicle), respectively) were started after the tumors were established, and the tumor volumes reached approximately 100 mm^3^. CTLA-4, cytotoxic T-lymphocyte–associated protein 4; hCTLA-4, humanized CTLA-4.
