# Supplementary material for: Artificial intelligence-powered discovery of small molecules inhibiting CTLA-4 in cancer
Source: BJC Rep. 2024 Jan 23;2:4. doi: 10.1038/s44276-023-00035-5 (PMC10838660; doi:10.1038/s44276-023-00035-5)
Supplement: Supplementary file 1 [file 44276_2023_35_MOESM10_ESM.pdf]

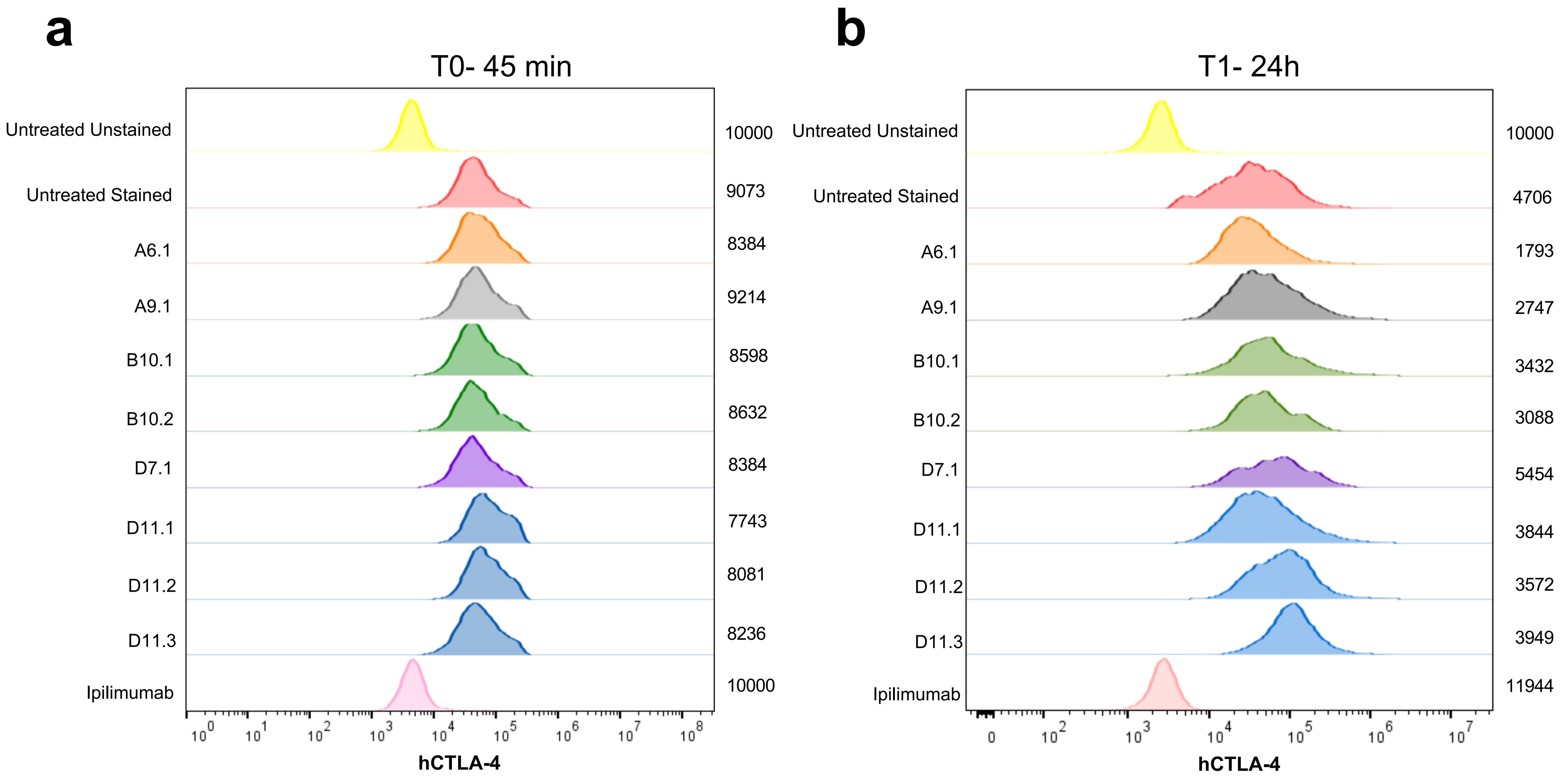
a
T0- 45 min
Untreated Unstained
Untreated Stained
A6.1
A9.1
B10.1
B10.2
D7.1
D11.1
D11.2
D11.3
Ipilimumab
10000
9073
8384
9214
8598
8632
8384
7743
8081
8236
10000
hCTLA-4
b
T1- 24h
Untreated Unstained
Untreated Stained
A6.1
A9.1
B10.1
B10.2
D7.1
D11.1
D11.2
D11.3
Ipilimumab
10000
4706
1793
2747
3432
3088
5454
3844
3572
3949
11944
hCTLA-4

c

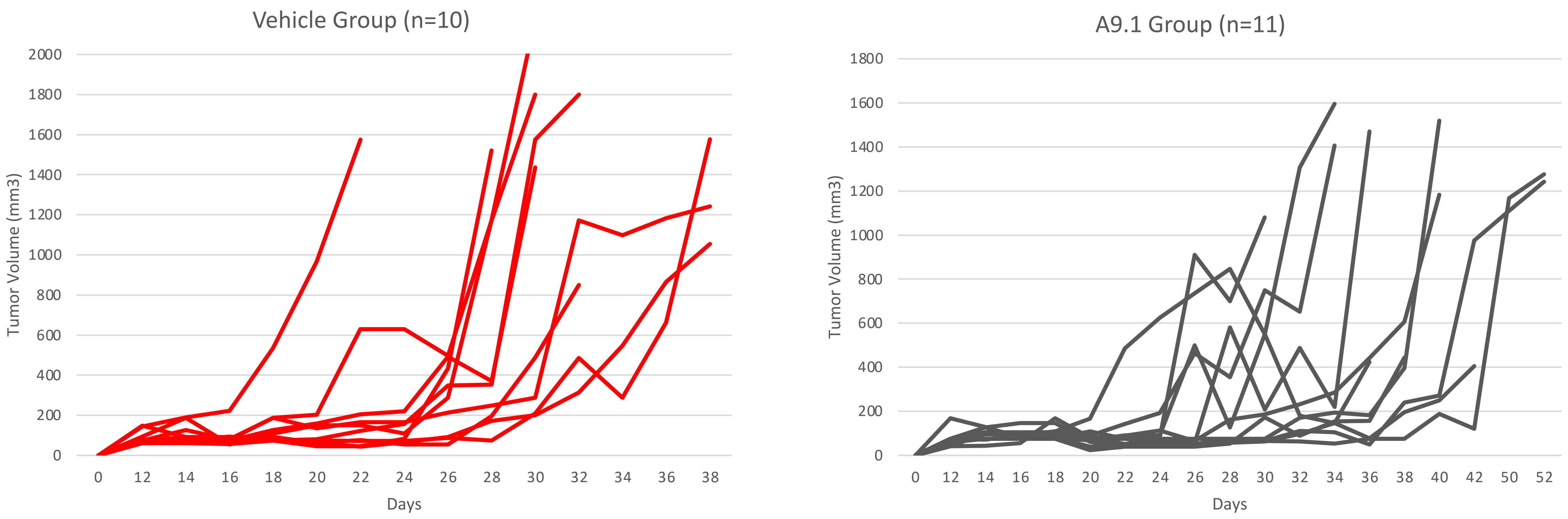
Vehicle Group (n=10)
Tumor Volume (mm3)
2000
1800
1600
1400
1200
1000
800
600
400
200
0
0 12 14 16 18 20 22 24 26 28 30 32 34 36 38
Days
A9.1 Group (n=11)
Tumor Volume (mm3)
1800
1600
1400
1200
1000
800
600
400
200
0
0 12 14 16 18 20 22 24 26 28 30 32 34 36 38 40 42 50 52
Days

d

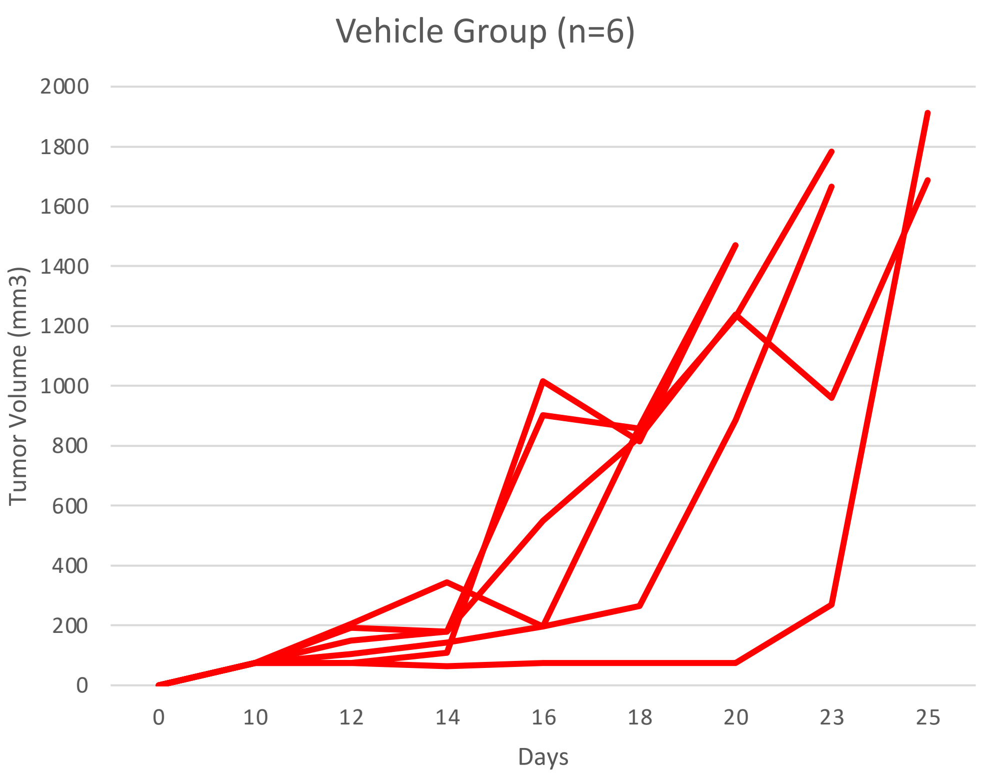
Vehicle Group (n=6)
Tumor Volume (mm3)
2000
1800
1600
1400
1200
1000
800
600
400
200
0
0
10
12
14
16
18
20
23
25
Days
D11.3 Group (n=5)
Tumor Volume (mm3)
2000
1800
1600
1400
1200
1000
800
600
400
200
0
0
10
12
14
16
18
20
23
25
27
29
31
Days
