# Supplementary material for: Artificial intelligence-powered discovery of small molecules inhibiting CTLA-4 in cancer
Source: BJC Rep. 2024 Jan 23;2:4. doi: 10.1038/s44276-023-00035-5 (PMC10838660; doi:10.1038/s44276-023-00035-5)
Supplement: Supplementary file 2 [file 44276_2023_35_MOESM3_ESM.pdf]

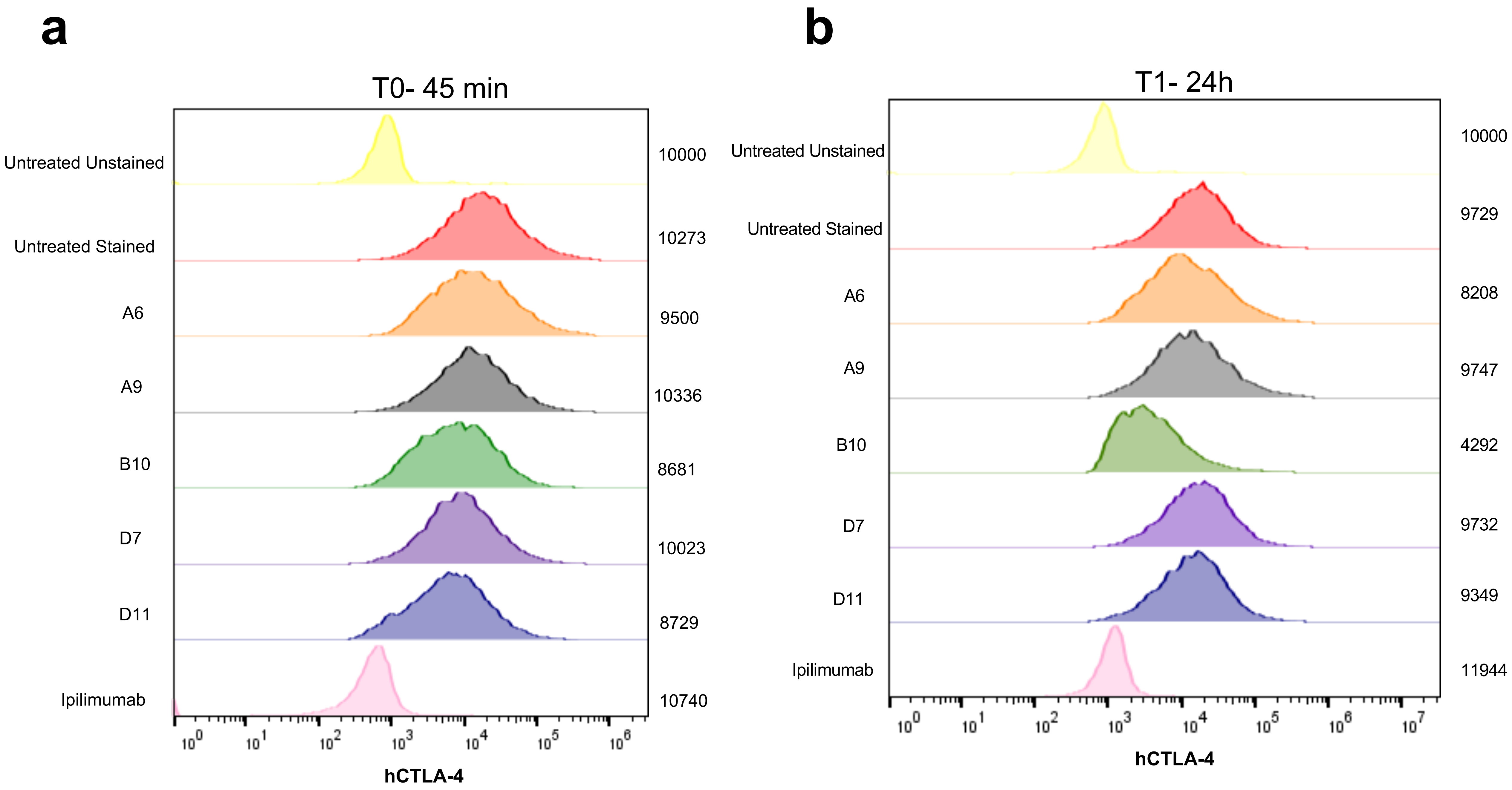
a
T0- 45 min
Untreated Unstained
Untreated Stained
A6
A9
B10
D7
D11
Ipilimumab
10000
10273
9500
10336
8681
10023
8729
10740
10^0 10^1 10^2 10^3 10^4 10^5 10^6
hCTLA-4
b
T1- 24h
Untreated Unstained
Untreated Stained
A6
A9
B10
D7
D11
Ipilimumab
10000
9729
8208
9747
4292
9732
9349
11944
10^0 10^1 10^2 10^3 10^4 10^5 10^6 10^7
hCTLA-4

c

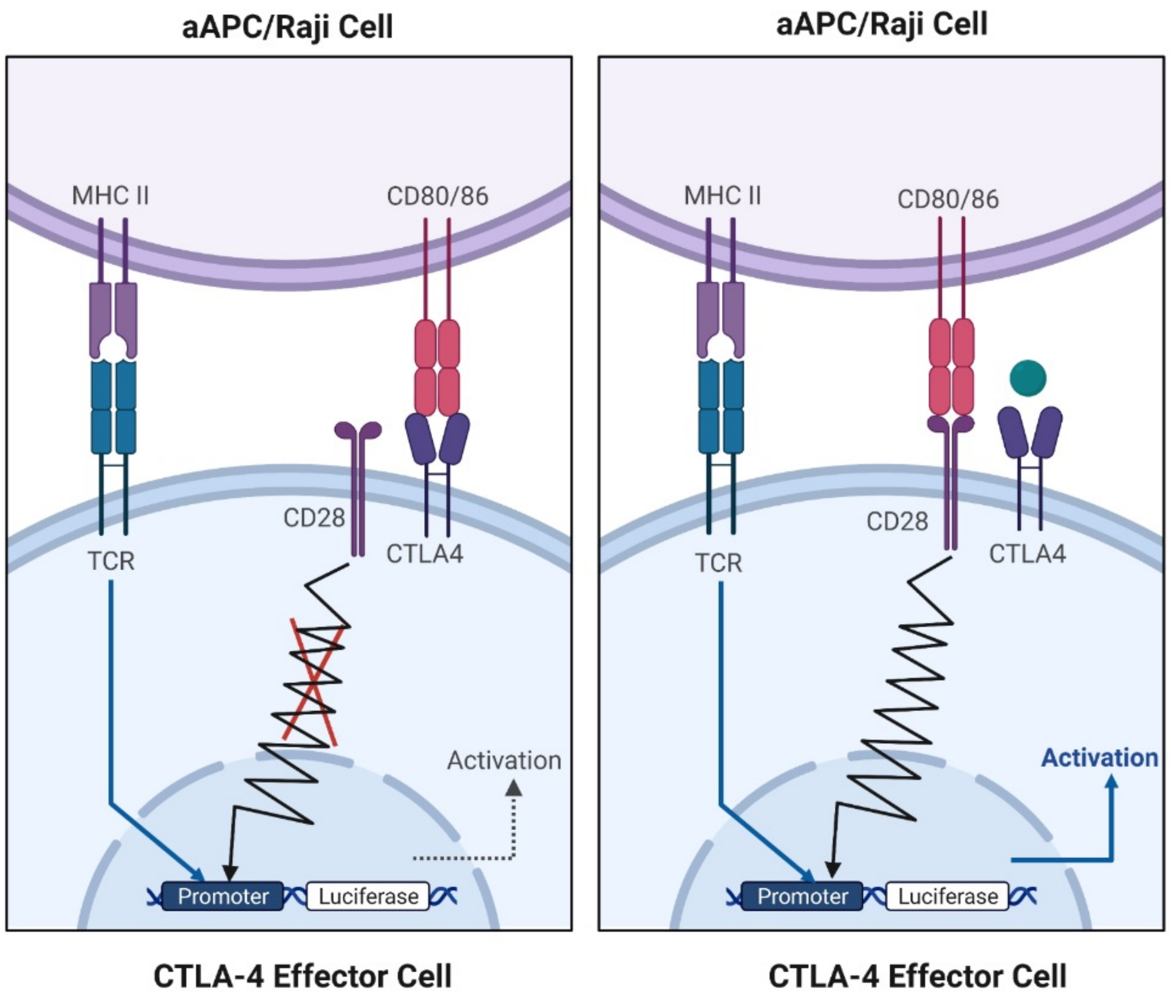
aAPC/Raji Cell
MHC II
CD80/86
CD28
TCR
CTLA4
Activation
Promoter
Luciferase
aAPC/Raji Cell
MHC II
CD80/86
CD28
TCR
CTLA4
Activation
Promoter
Luciferase
CTLA-4 Effector Cell
CTLA-4 Effector Cell

d

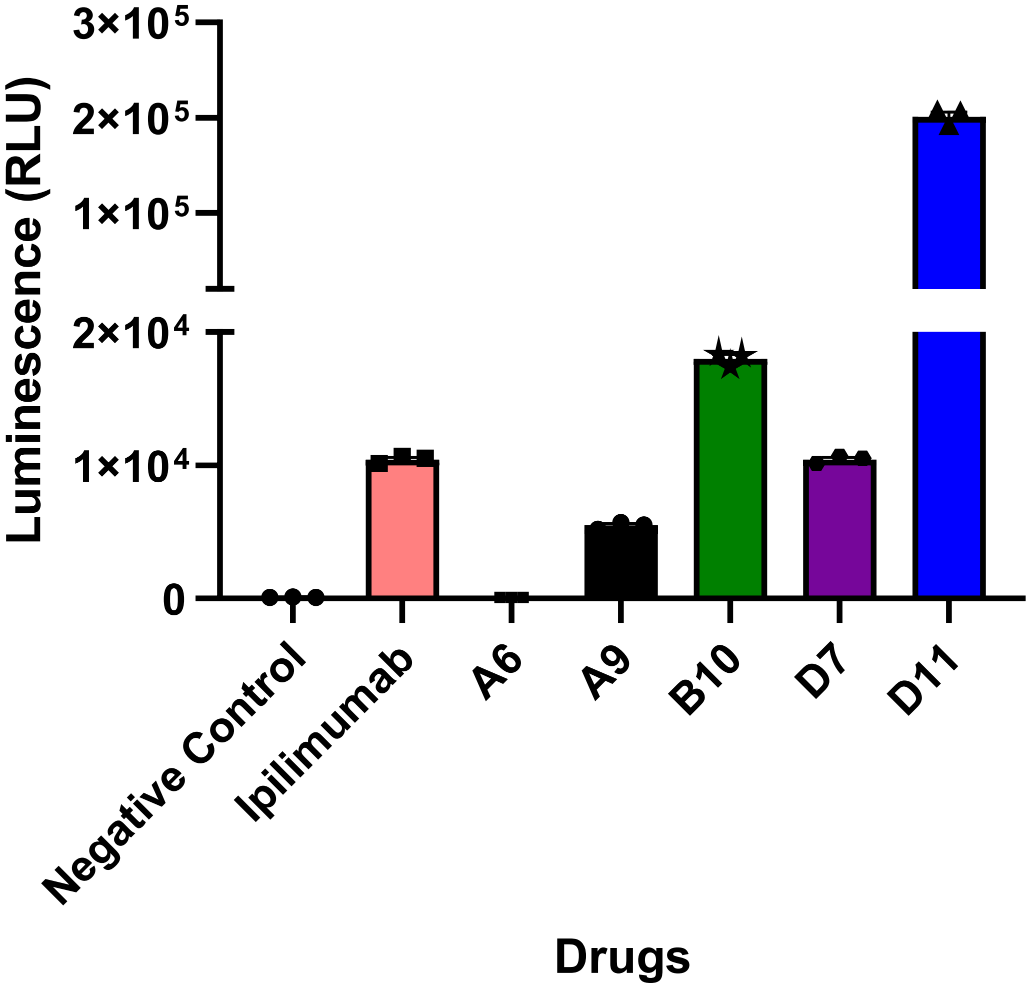
Luminescence (RLU)
3×10^5
2×10^5
1×10^5
2×10^4
1×10^4
0
Negative Control
Ipilimumab
A6
A9
B10
D7
D11
Drugs
