# Supplementary material for: Artificial intelligence-powered discovery of small molecules inhibiting CTLA-4 in cancer
Source: BJC Rep. 2024 Jan 23;2:4. doi: 10.1038/s44276-023-00035-5 (PMC10838660; doi:10.1038/s44276-023-00035-5)
Supplement: Supplementary file 3 [file 44276_2023_35_MOESM4_ESM.pdf]

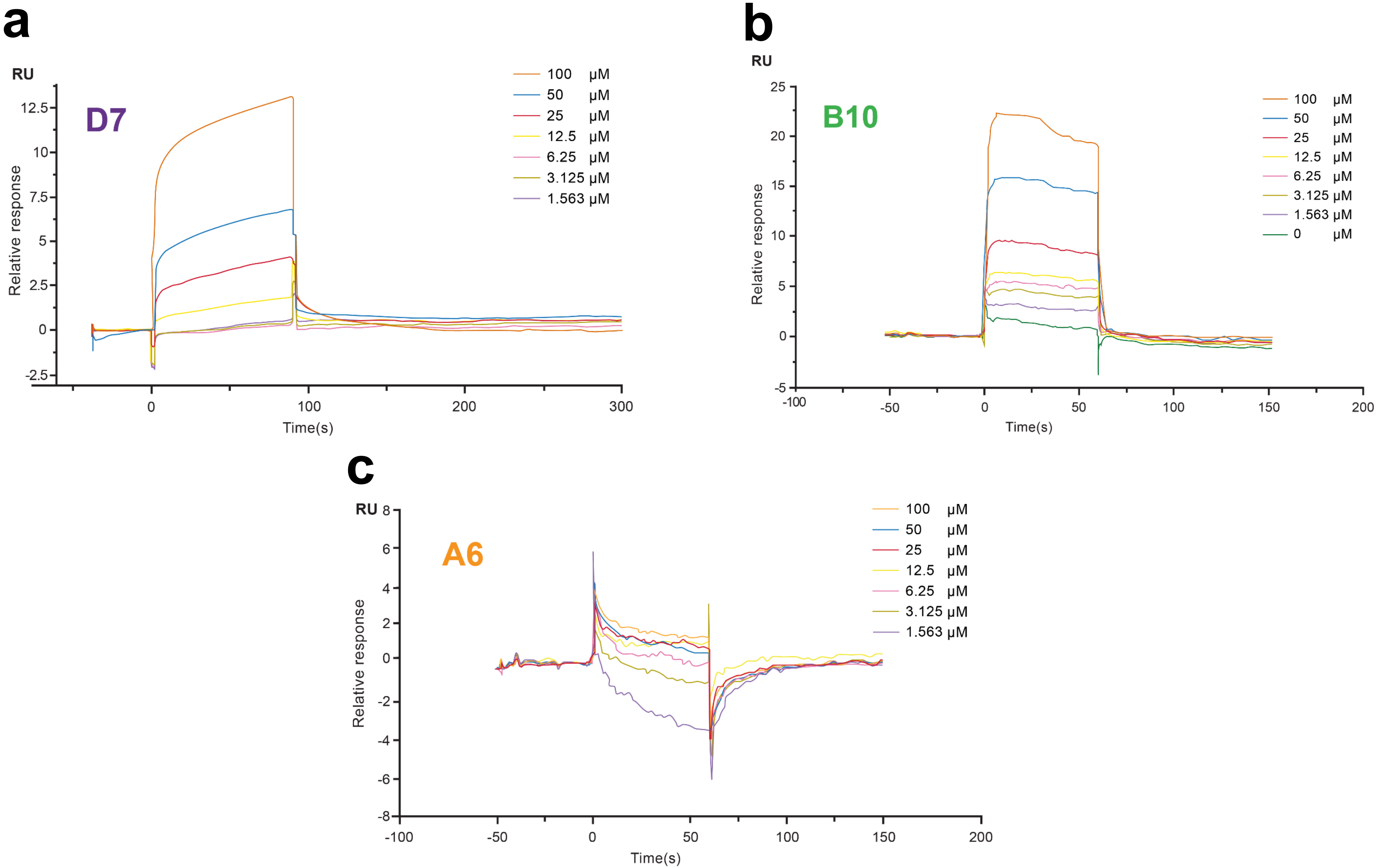
a
RU
D7
Relative response
Time(s)
12.5
10
7.5
5
2.5
0
-2.5
0
100
200
300
100 μM
50 μM
25 μM
12.5 μM
6.25 μM
3.125 μM
1.563 μM
b
RU
B10
Relative response
Time(s)
25
20
15
10
5
0
-5
-100
-50
0
50
100
150
200
100 μM
50 μM
25 μM
12.5 μM
6.25 μM
3.125 μM
1.563 μM
0 μM
c
RU
A6
Relative response
Time(s)
8
6
4
2
0
-2
-4
-6
-8
-100
-50
0
50
100
150
200
100 μM
50 μM
25 μM
12.5 μM
6.25 μM
3.125 μM
1.563 μM
