# Supplementary material for: Artificial intelligence-powered discovery of small molecules inhibiting CTLA-4 in cancer
Source: BJC Rep. 2024 Jan 23;2:4. doi: 10.1038/s44276-023-00035-5 (PMC10838660; doi:10.1038/s44276-023-00035-5)
Supplement: Supplementary file 4 [file 44276_2023_35_MOESM5_ESM.pdf]

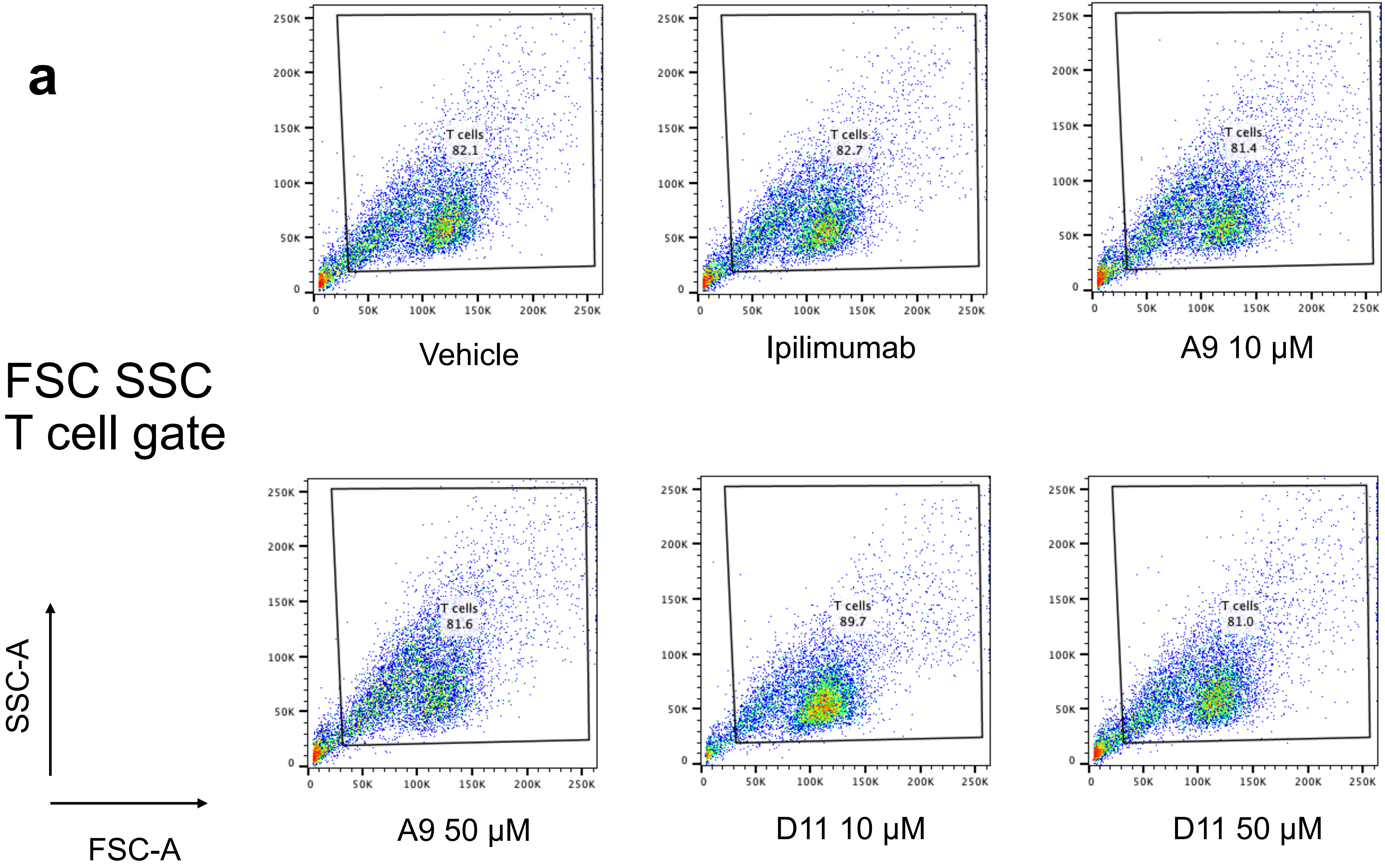
a
FSC SSC
T cell gate
T cells
82.1
Vehicle
T cells
82.7
Ipilimumab
T cells
81.4
A9 10 μM
T cells
81.6
A9 50 μM
T cells
89.7
D11 10 μM
T cells
81.0
D11 50 μM
SSC-A
FSC-A
0
50K
100K
150K
200K
250K

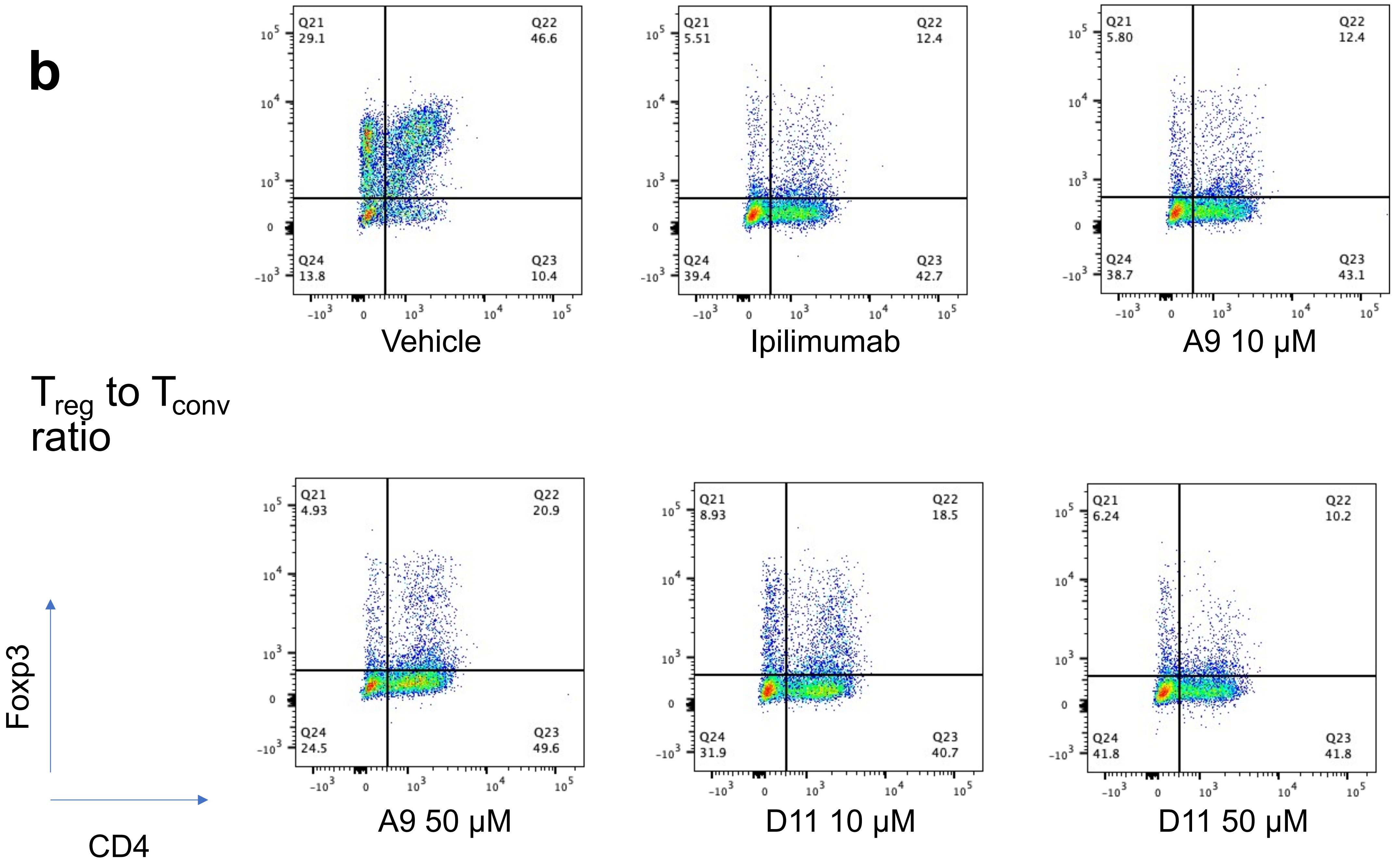
b
Treg to Tconv ratio
Foxp3
CD4
Q21 29.1
Q22 46.6
Q24 13.8
Q23 10.4
Vehicle
Q21 5.51
Q22 12.4
Q24 39.4
Q23 42.7
Ipilimumab
Q21 5.80
Q22 12.4
Q24 38.7
Q23 43.1
A9 10 μM
Q21 4.93
Q22 20.9
Q24 24.5
Q23 49.6
A9 50 μM
Q21 8.93
Q22 18.5
Q24 31.9
Q23 40.7
D11 10 μM
Q21 6.24
Q22 10.2
Q24 41.8
Q23 41.8
D11 50 μM

c

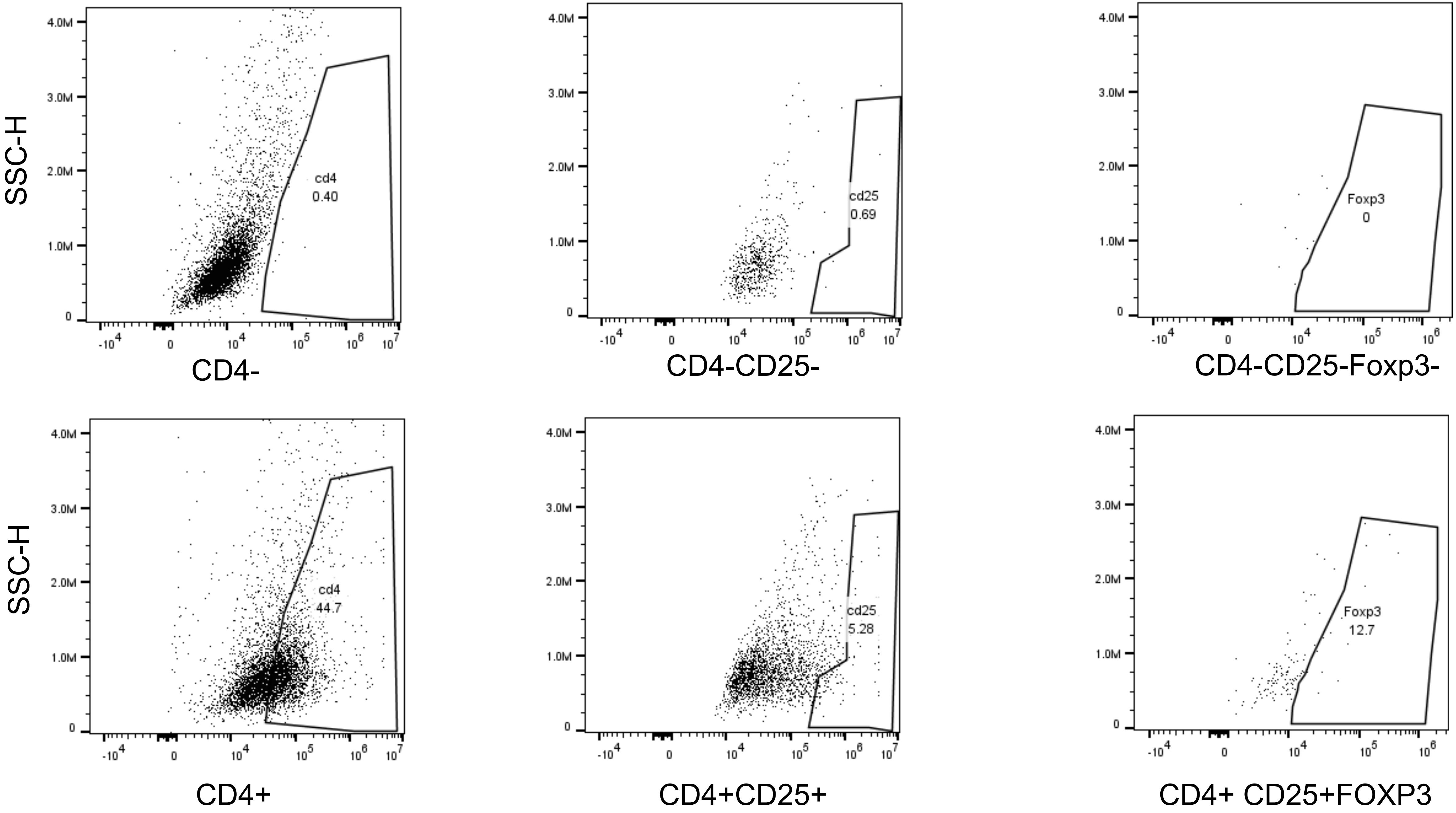

SSC-H
4.0M
3.0M
2.0M
1.0M
0
-10^4
0
10^4
10^5
10^6
10^7
cd4
0.40
CD4-
cd25
0.69
CD4-CD25-
Foxp3
0
CD4-CD25-Foxp3-
cd4
44.7
CD4+
cd25
5.28
CD4+CD25+
Foxp3
12.7
CD4+ CD25+FOXP3

d

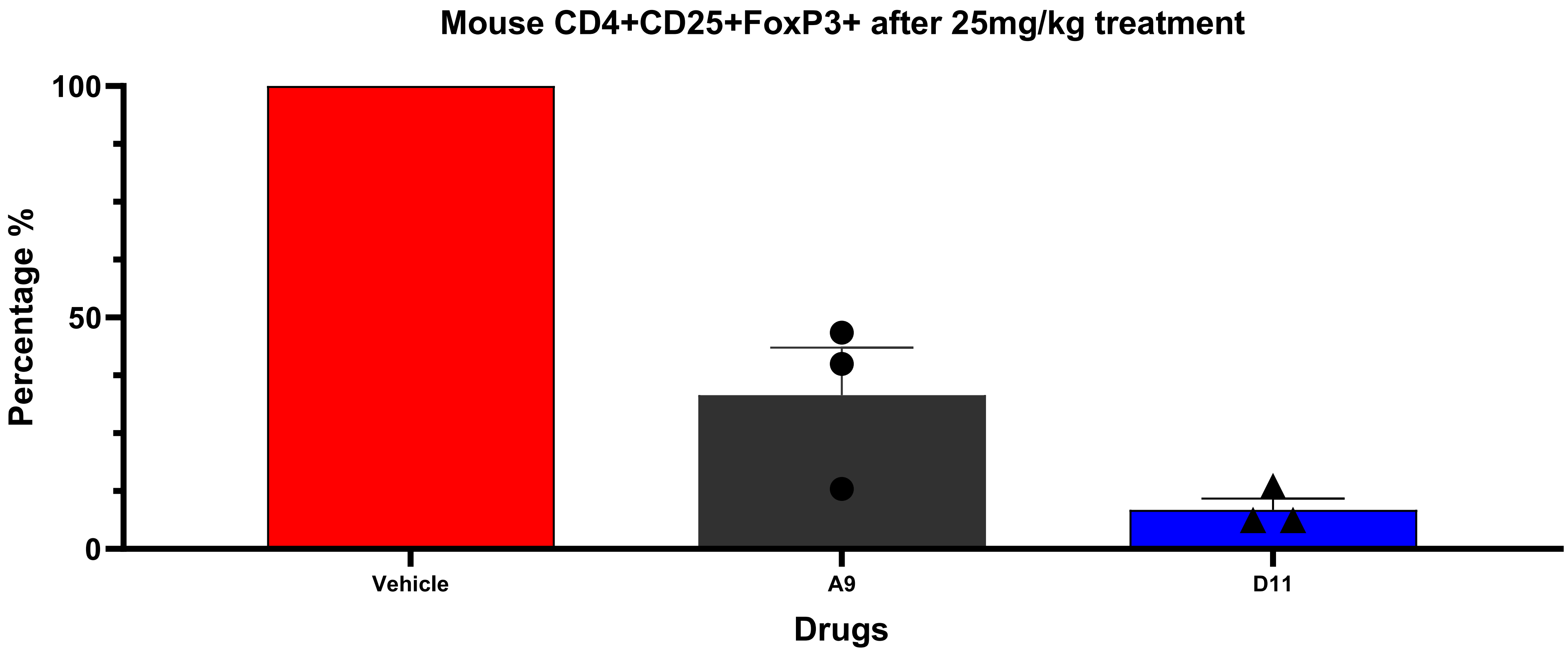
Mouse CD4+CD25+FoxP3+ after 25mg/kg treatment
Percentage %
100
50
0
Vehicle
A9
D11
Drugs
