# Supplementary material for: Artificial intelligence-powered discovery of small molecules inhibiting CTLA-4 in cancer
Source: BJC Rep. 2024 Jan 23;2:4. doi: 10.1038/s44276-023-00035-5 (PMC10838660; doi:10.1038/s44276-023-00035-5)
Supplement: Supplementary file 5 [file 44276_2023_35_MOESM6_ESM.pdf]

a

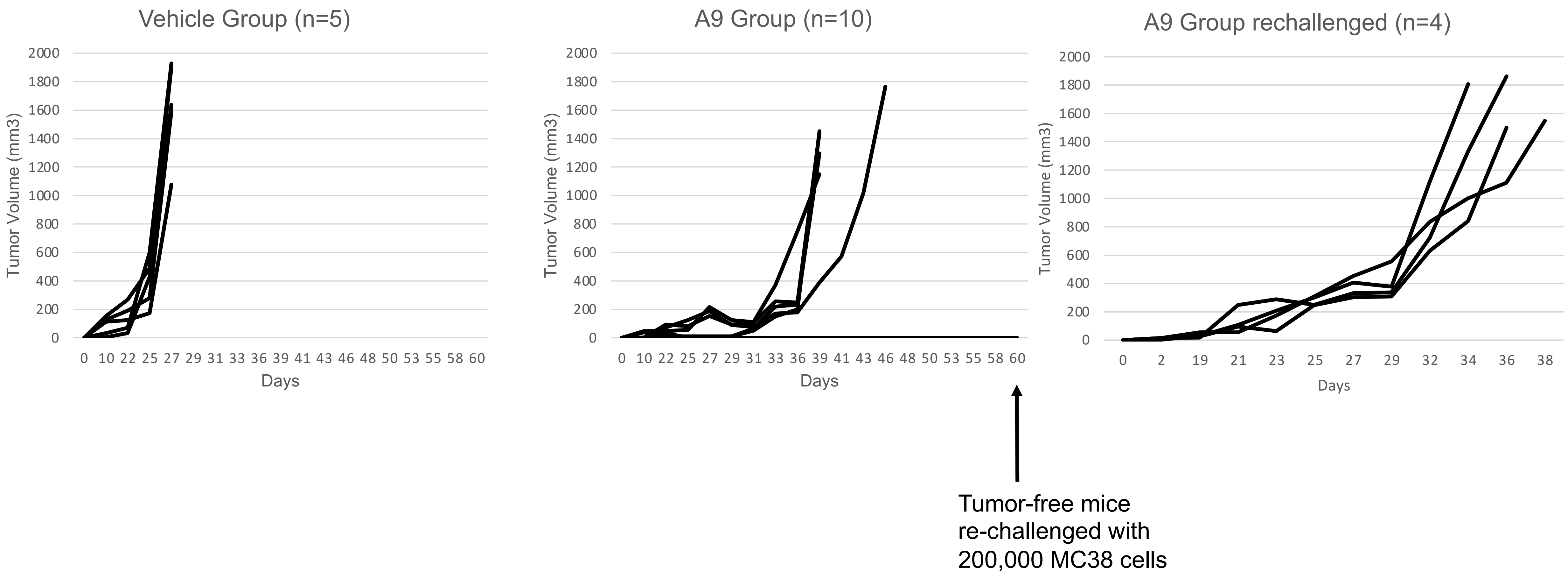
Vehicle Group (n=5)
Tumor Volume (mm3)
2000
1800
1600
1400
1200
1000
800
600
400
200
0
0 10 22 25 27 29 31 33 36 39 41 43 46 48 50 53 55 58 60
Days
A9 Group (n=10)
Tumor Volume (mm3)
2000
1800
1600
1400
1200
1000
800
600
400
200
0
0 10 22 25 27 29 31 33 36 39 41 43 46 48 50 53 55 58 60
Days
A9 Group rechallenged (n=4)
Tumor Volume (mm3)
2000
1800
1600
1400
1200
1000
800
600
400
200
0
0 2 19 21 23 25 27 29 32 34 36 38
Days
Tumor-free mice
re-challenged with
200,000 MC38 cells

b

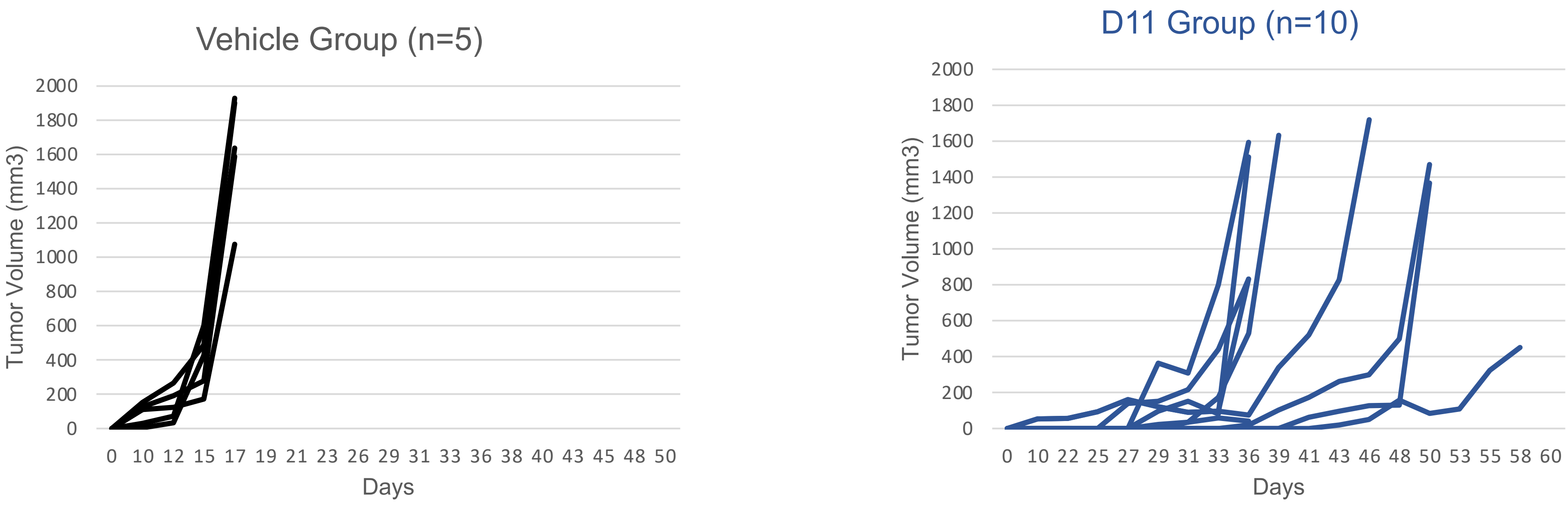
Vehicle Group (n=5)
Tumor Volume (mm3)
2000
1800
1600
1400
1200
1000
800
600
400
200
0
0 10 12 15 17 19 21 23 26 29 31 33 36 38 40 43 45 48 50
Days
D11 Group (n=10)
Tumor Volume (mm3)
2000
1800
1600
1400
1200
1000
800
600
400
200
0
0 10 22 25 27 29 31 33 36 39 41 43 46 48 50 53 55 58 60
Days

c

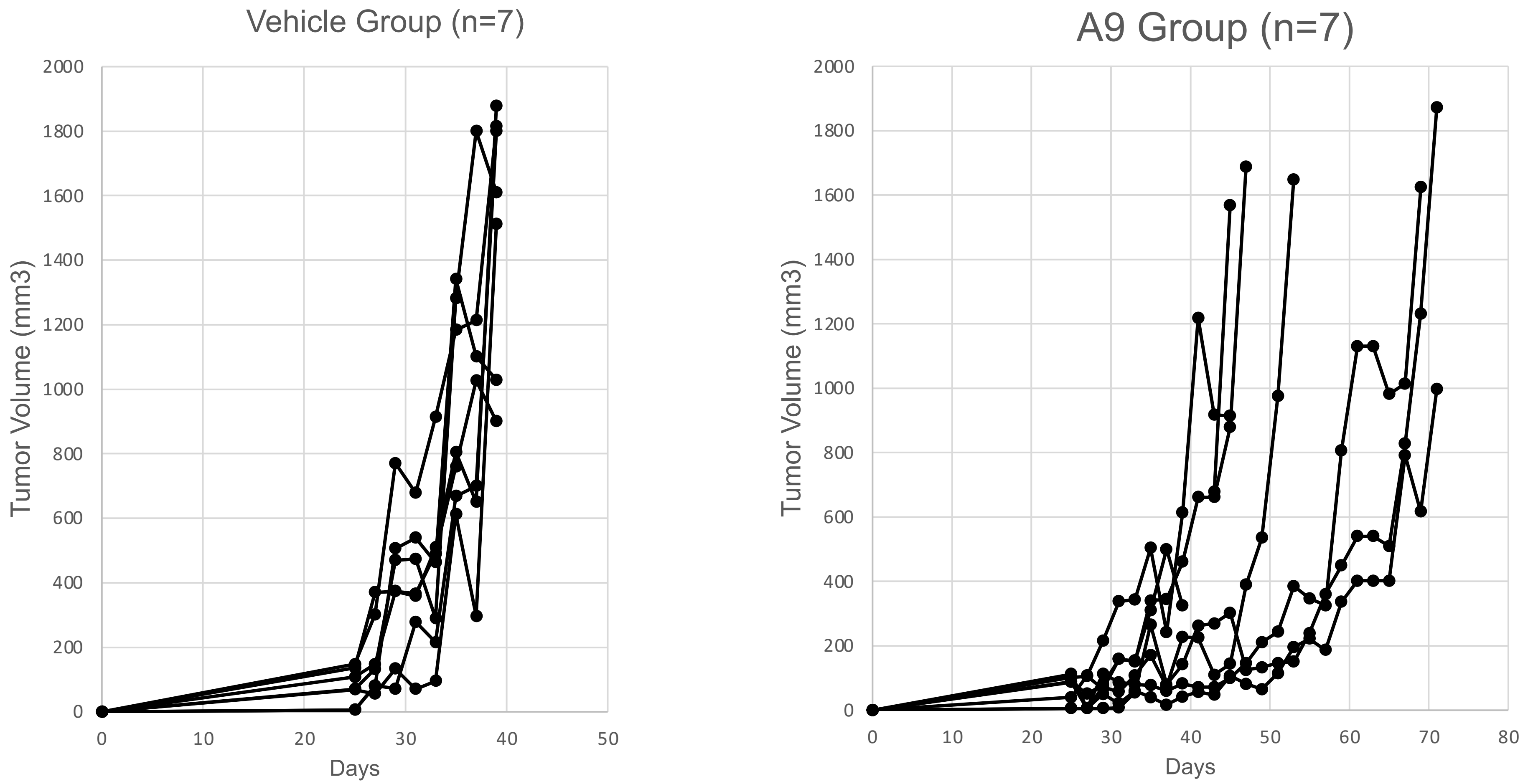

Vehicle Group (n=7)
Tumor Volume (mm3)
Days
0
200
400
600
800
1000
1200
1400
1600
1800
2000
0
10
20
30
40
50
A9 Group (n=7)
Tumor Volume (mm3)
Days
0
200
400
600
800
1000
1200
1400
1600
1800
2000
0
10
20
30
40
50
60
70
80

d

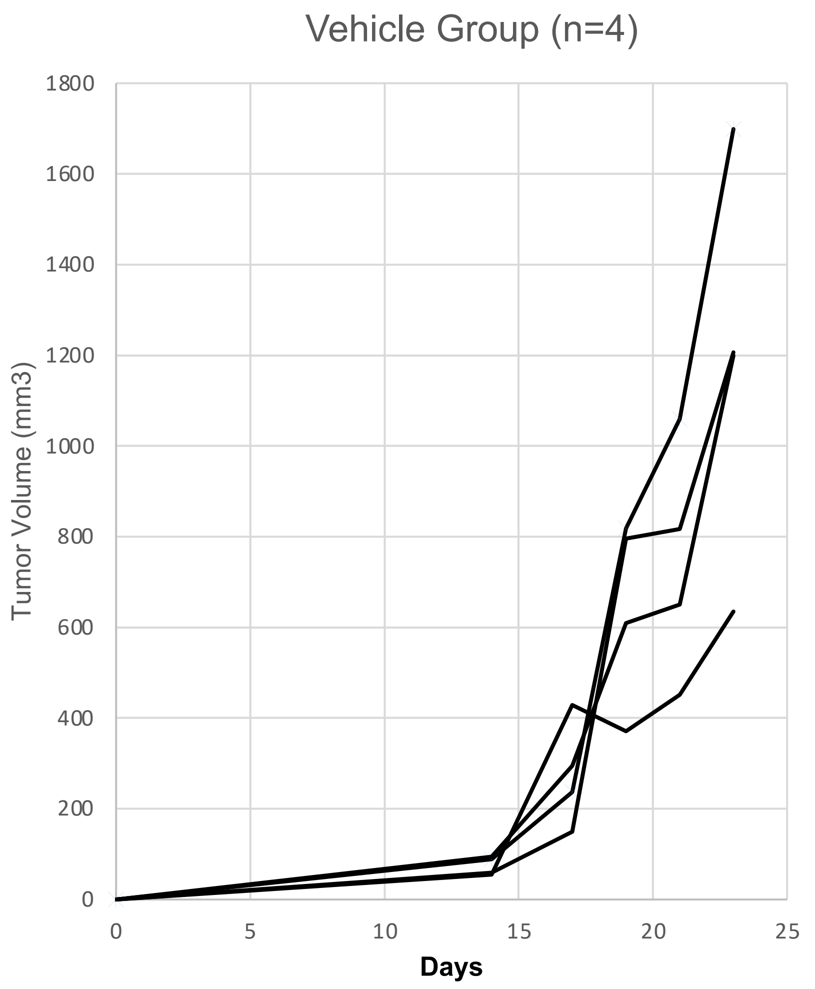
Vehicle Group (n=4)
Tumor Volume (mm3)
Days
0
200
400
600
800
1000
1200
1400
1600
1800
0
5
10
15
20
25

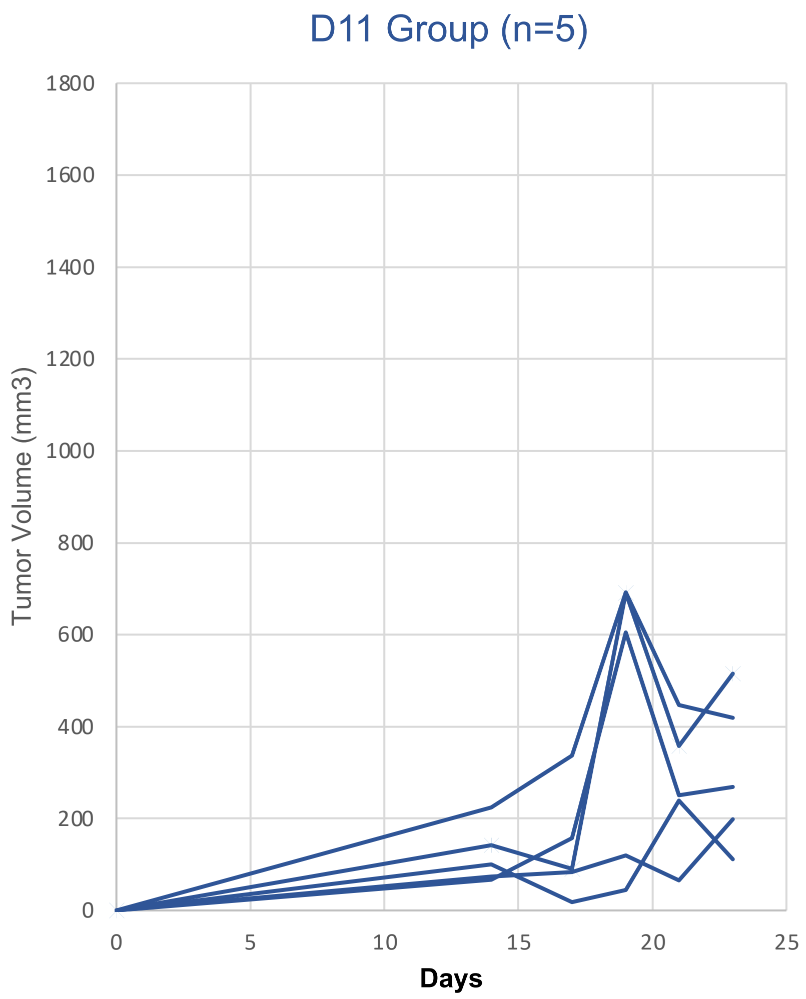
D11 Group (n=5)
Tumor Volume (mm3)
Days
0
200
400
600
800
1000
1200
1400
1600
1800
0
5
10
15
20
25
