# Supplementary material for: Artificial intelligence-powered discovery of small molecules inhibiting CTLA-4 in cancer
Source: BJC Rep. 2024 Jan 23;2:4. doi: 10.1038/s44276-023-00035-5 (PMC10838660; doi:10.1038/s44276-023-00035-5)
Supplement: Supplementary file 6 [file 44276_2023_35_MOESM7_ESM.pdf]

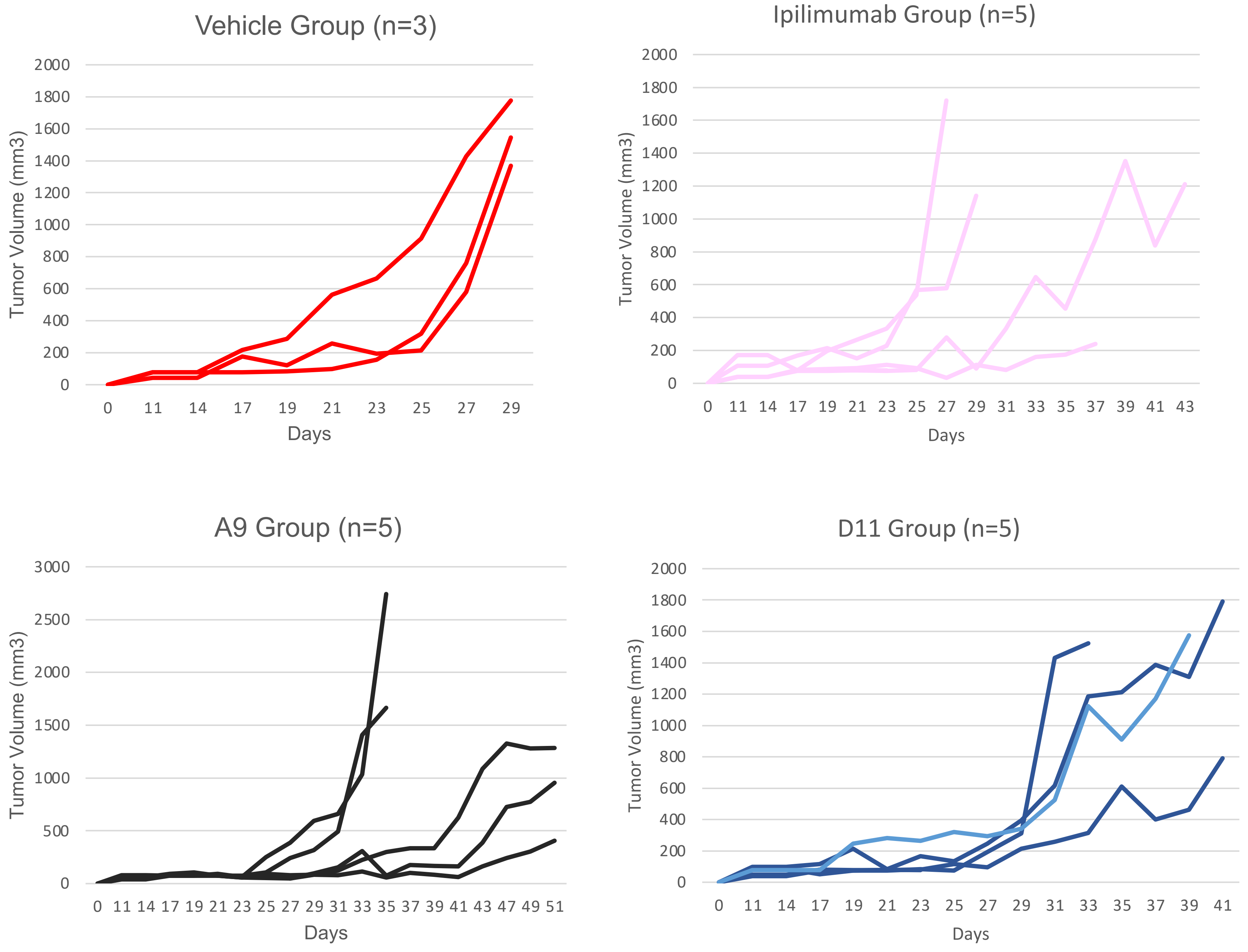
Vehicle Group (n=3)
Tumor Volume (mm3)
2000 1800 1600 1400 1200 1000 800 600 400 200 0
0 11 14 17 19 21 23 25 27 29
Days
Ipilimumab Group (n=5)
Tumor Volume (mm3)
2000 1800 1600 1400 1200 1000 800 600 400 200 0
0 11 14 17 19 21 23 25 27 29 31 33 35 37 39 41 43
Days
A9 Group (n=5)
Tumor Volume (mm3)
3000 2500 2000 1500 1000 500 0
0 11 14 17 19 21 23 25 27 29 31 33 35 37 39 41 43 47 49 51
Days
D11 Group (n=5)
Tumor Volume (mm3)
2000 1800 1600 1400 1200 1000 800 600 400 200 0
0 11 14 17 19 21 23 25 27 29 31 33 35 37 39 41
Days
