# Supplementary material for: Artificial intelligence-powered discovery of small molecules inhibiting CTLA-4 in cancer
Source: BJC Rep. 2024 Jan 23;2:4. doi: 10.1038/s44276-023-00035-5 (PMC10838660; doi:10.1038/s44276-023-00035-5)
Supplement: Supplementary file 7 [file 44276_2023_35_MOESM8_ESM.pdf]

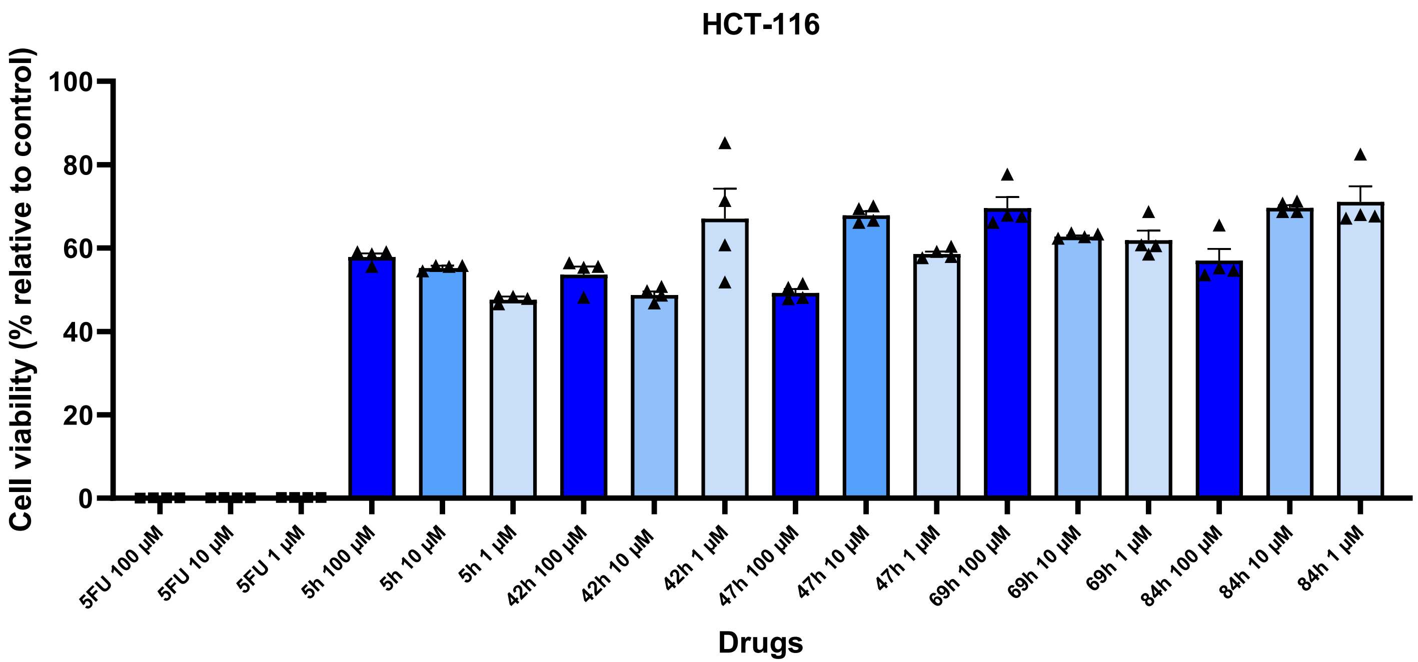

HCT-116
Cell viability (% relative to control)
100
80
60
40
20
0
5FU 100 μM
5FU 10 μM
5FU 1 μM
5h 100 μM
5h 10 μM
5h 1 μM
42h 100 μM
42h 10 μM
42h 1 μM
47h 100 μM
47h 10 μM
47h 1 μM
69h 100 μM
69h 10 μM
69h 1 μM
84h 100 μM
84h 10 μM
84h 1 μM
Drugs

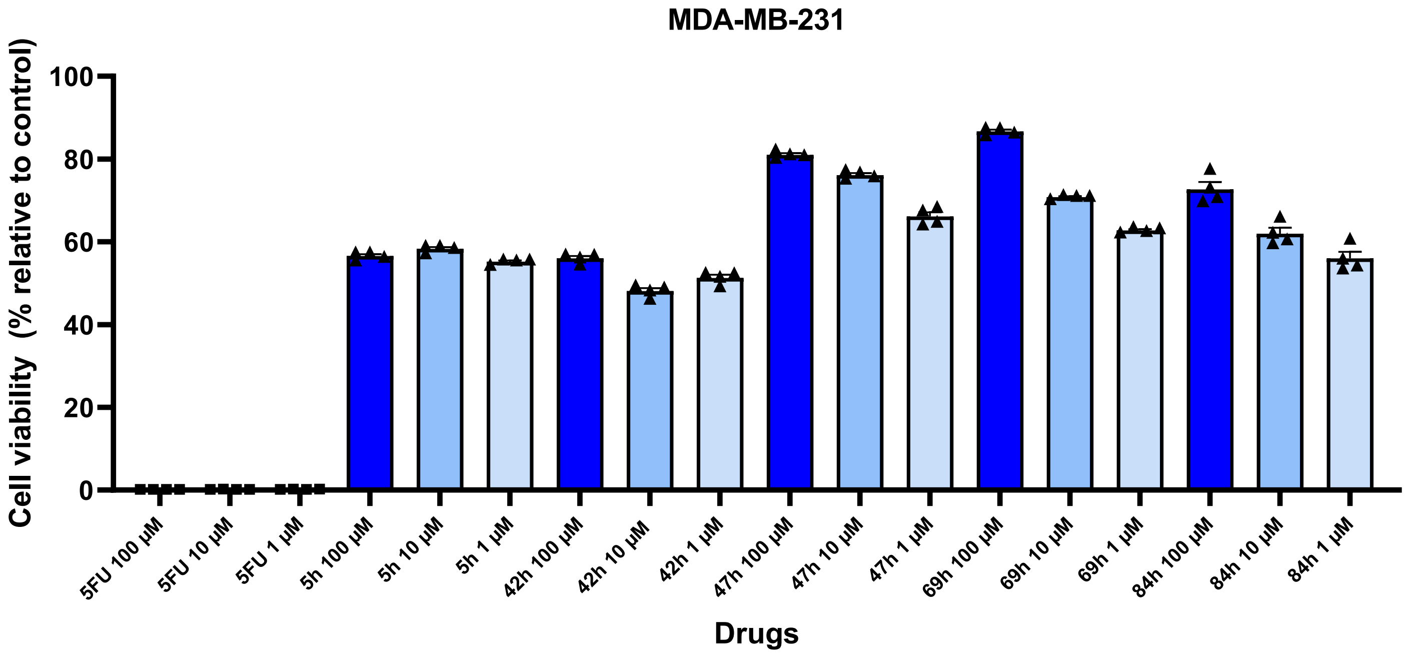

MDA-MB-231
Cell viability (% relative to control)
100
80
60
40
20
0
5FU 100 μM
5FU 10 μM
5FU 1 μM
5h 100 μM
5h 10 μM
5h 1 μM
42h 100 μM
42h 10 μM
42h 1 μM
47h 100 μM
47h 10 μM
47h 1 μM
69h 100 μM
69h 10 μM
69h 1 μM
84h 100 μM
84h 10 μM
84h 1 μM
Drugs
