# Supplementary material for: Artificial intelligence-powered discovery of small molecules inhibiting CTLA-4 in cancer
Source: BJC Rep. 2024 Jan 23;2:4. doi: 10.1038/s44276-023-00035-5 (PMC10838660; doi:10.1038/s44276-023-00035-5)
Supplement: Supplementary file 8 [file 44276_2023_35_MOESM9_ESM.pdf]

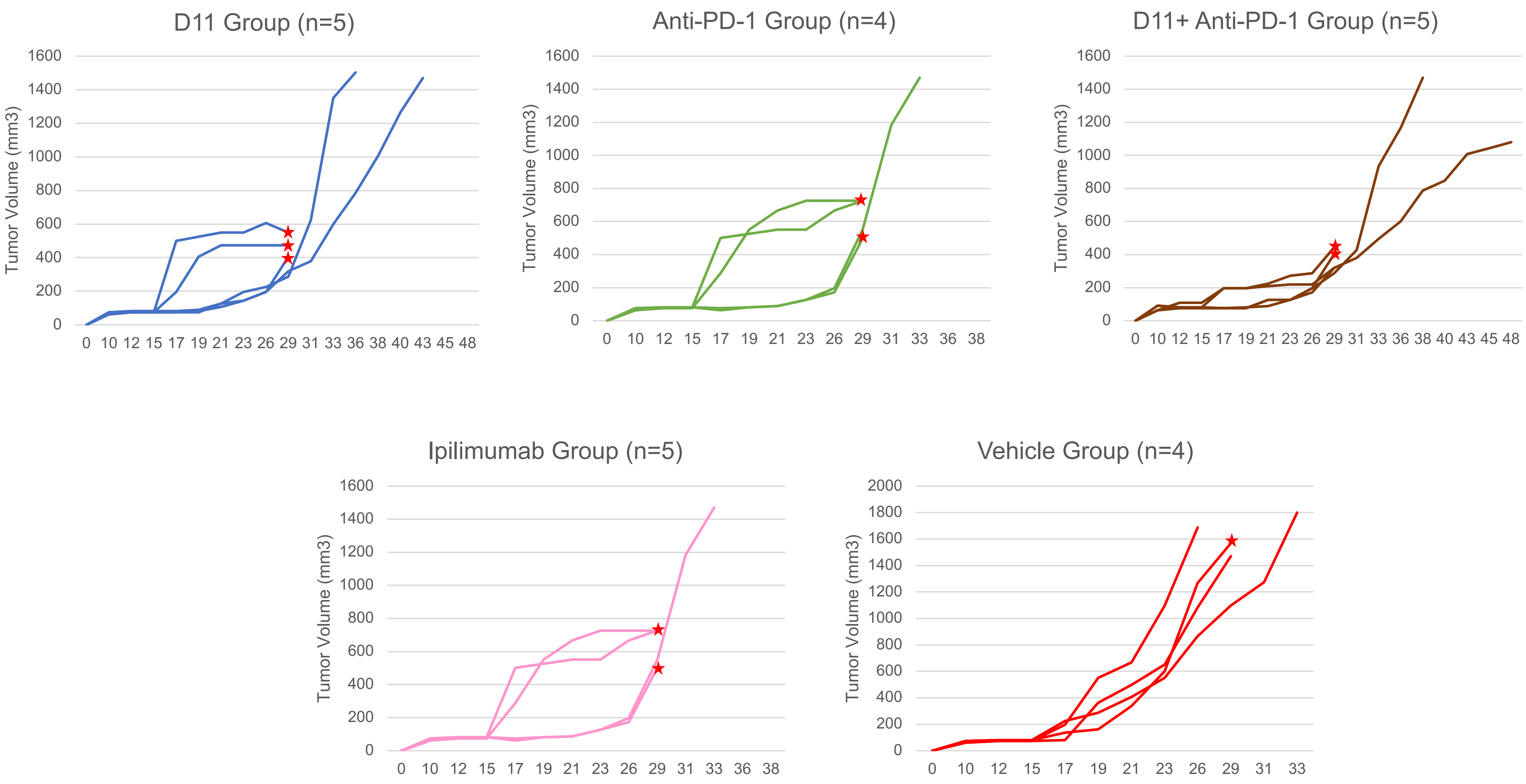
D11 Group (n=5)
Tumor Volume (mm3)
0 200 400 600 800 1000 1200 1400 1600
0 10 12 15 17 19 21 23 26 29 31 33 36 38 40 43 45 48
Anti-PD-1 Group (n=4)
Tumor Volume (mm3)
0 200 400 600 800 1000 1200 1400 1600
0 10 12 15 17 19 21 23 26 29 31 33 36 38
D11+ Anti-PD-1 Group (n=5)
Tumor Volume (mm3)
0 200 400 600 800 1000 1200 1400 1600
0 10 12 15 17 19 21 23 26 29 31 33 36 38 40 43 45 48
Ipilimumab Group (n=5)
Tumor Volume (mm3)
0 200 400 600 800 1000 1200 1400 1600
0 10 12 15 17 19 21 23 26 29 31 33 36 38
Vehicle Group (n=4)
Tumor Volume (mm3)
0 200 400 600 800 1000 1200 1400 1600 1800 2000
0 10 12 15 17 19 21 23 26 29 31 33
